# Pairwise running of automated crystallographic model-building pipelines

**DOI:** 10.1107/S2059798320010542

**Published:** 2020-08-19

**Authors:** Emad Alharbi, Radu Calinescu, Kevin Cowtan

**Affiliations:** a Department of Computer Science, University of York, Heslington, York YO10 5GH, United Kingdom; b Department of Information Technology, University of Tabuk, Tabuk, Saudi Arabia; c Department of Chemistry, University of York, Heslington, York YO10 5DD, United Kingdom

**Keywords:** structure solution, model building, software

## Abstract

For the last two decades, researchers have worked independently to automate protein model building, and four widely used software pipelines have been developed for this purpose: *ARP*/*wARP*, *Buccaneer*, *Phenix AutoBuild* and *SHELXE*. Here, the usefulness of combining these pipelines to improve the built protein structures by running them in pairwise combinations is examined. The results show that integrating these pipelines can lead to significant improvements in structure completeness and *R*_free_. In particular, running *Phenix AutoBuild* after *Buccaneer* improved structure completeness for 29% and 75% of the data sets that were examined at the original resolution and at a simulated lower resolution, respectively, compared with running *Phenix AutoBuild* on its own. In contrast, *Phenix AutoBuild* alone produced better structure completeness than the two pipelines combined for only 7% and 3% of these data sets.

## Abstract 2

This study shows the usefulness of integrating automated crystallographic model-building pipelines. We ran the four most used pipelines (*ARP*/*wARP*, *Buccaneer*, *Phenix AutoBuild* and *SHELXE*) alone and in pairwise combinations, and compared the structures that they produced based on structure completeness and *R*_free_.

## 1. Introduction   

X-ray crystallography has been used for several decades for the determination of structures of proteins and RNA/DNA, including 90% of the protein structures deposited in the Protein Data Bank as of 2020 (Berman *et al.*, 2000 ▸; RCSB PDB, 2020 ▸). Multiple steps are required to obtain a protein structure, starting with the crystallization process, obtaining an electron-density map from the diffraction pattern and building the protein structure. Researchers have investigated ways to automate the building step, and four widely used pipelines have been developed: *ARP*/*wARP* (Perrakis *et al.*, 1999 ▸; Lamzin & Wilson, 1993 ▸; Morris *et al.*, 2003 ▸; Langer *et al.*, 2008 ▸, 2013 ▸), *Buccaneer* (Cowtan, 2006 ▸, 2008 ▸), *Phenix AutoBuild* (Terwilliger *et al.*, 2008 ▸; Liebschner *et al.*, 2019 ▸) and *SHELXE* (Sheldrick, 2008 ▸, 2010 ▸; Thorn & Sheldrick, 2013 ▸; Usón & Sheldrick, 2018 ▸). RNA/DNA can also be built automatically by *Phenix AutoBuild* and other tools. The performance of these pipelines varies depending on electron-density map-quality indicators such as resolution and phases. In recent work, we conducted a comparison of these pipelines, and we found that the performance of the pipelines differs from one structure to another, which suggests that there is no best pipeline for all protein structures, although there is often a best pipeline for each protein structure (Alharbi *et al.*, 2019 ▸).

Researchers have focused on different aspects of the protein-building problem and have developed appropriate methods depending on the coverage of their test data sets. As a result, pipelines tend to perform well when they are run using data sets with similar features to those that were used in developing the pipeline. Having data sets with different features generally makes the pipelines perform poorly. We addressed this matter here by running the pipelines in pairwise combinations, in which the first pipeline in the combination built a protein structure as an initial structure for the second pipeline. Using these pairwise pipeline combinations often improved the final protein structure compared with using only one pipeline.

## 2. Data sets   

We used the original data sets from van den Bedem *et al.* (2011 ▸), which have resolutions of between 1.9 and 3.2 Å, and synthetic data sets obtained by truncating the original data sets to 3.2, 3.4, 3.6, 3.8 and 4.0 Å (synthetic resolutions) as described in our recent crystallographic model-building pipeline-comparison paper (Alharbi *et al.*, 2019 ▸). As in our comparison paper, 52 original data sets that were used in the development of *Buccaneer* and their truncated resolutions were omitted from the main results (and are only presented in the supporting information). This gave us 202 original and 1009 synthetic resolution data sets initially, and 150 original and 750 synthetic resolution data sets after omitting the *Buccaneer* development data sets.

Similarly large data sets of over 1000 structures have recently been used to improve *ARP*/*wARP* (Chojnowski *et al.*, 2020 ▸). However, we were unable to use these data sets because this paper builds on our recent crystallographic model-building pipeline-comparison work (Alharbi *et al.*, 2019 ▸), which used the original and synthetic data sets described above.

Density modification was performed by *Parrot* (Cowtan, 2010 ▸). Phase improvement was performed on the experimental phasing data, but NCS averaging was not used for those structures where NCS was present, with the aim of providing starting data with poorer phases both to test the limits of the model-building algorithms and to better simulate the poorer phases that are typically associated with lower resolution data sets.

## 3. Method for pairwise running   

We ran the same versions of the pipelines as in our previous comparison paper (Alharbi *et al.*, 2019 ▸) to compare individual pipelines with combined pipelines. The versions were *Phenix AutoBuild* version 1.14, *Buccaneer* in *CCP*4*i*, *ARP*/*wARP* version 8 and *SHELXE* version 2019/1. We used a 173-node high-performance cluster with 7024 Intel Xeon Gold/Platinum cores and a total memory of 42 TB. We allowed a maximum of 48 h for the building of each structure because this was the highest time limit that the majority of our cluster nodes allowed.

Unlike in our previous comparison paper, here we tried to achieve the best performance of the pipelines, and to do this we changed the default parameters as necessary. ‘Rebuild in place’ is a feature of *Phenix AutoBuild* that improves the input structure without adding or removing residues, and it is used by default when the input structure is close to the correct structure (Terwilliger *et al.*, 2008 ▸). *Phenix AutoBuild* is unable to use ‘rebuild in place’ when the initial structure contains unknown residues that cause a mismatch between the input model chains and the model sequence. This occurred in 13.7% and 3.5% of the structures built by *Buccaneer* and *ARP*/*wARP*, respectively. We forced *Phenix AutoBuild* not to use this feature if it failed in the first attempt. An alternative workaround for this scenario is to remove the unknown residues before using the initial structure in *Phenix AutoBuild*.

*SHELXE* was not run after other pipelines because it only builds the main chain, while the other pipelines build complete structures. However, *SHELXE* structures were used as the initial structure for input to other pipelines. Additionally, *SHELXE* structures were only built for the original resolution data sets, as the synthetic structures fall outside the resolution range recommended for *SHELXE*.

The evaluation measures that we considered were structure completeness calculated from the deposited model and *R*_free_. Structure completeness represents the percentage of atoms from the built structure with coordinates within 1.0 Å of the corresponding atoms from the deposited structure with the same residue type. *R*_free_ was obtained by running ‘zero-cycle’ *REFMAC *(Murshudov *et al.*, 2011 ▸) to avoid the effect of the different parameterizations used by different refinement programs. The different model parameterizations used by different model-building programs lead to overfitting and the underestimation of *R*_work_ in some cases, so we focus on *R*_free_ in this comparison. While the use of a free set is not normally recommended for *ARP*/*wARP*, in this paper we are not primarily interested in individual pipeline performance, so we used a free set for analysis purposes (Chojnowski *et al.*, 2020 ▸). *ARP*/*wARP* does not necessarily set aside the same free reflections as the other pipelines, so the *REFMAC* evaluation step was changed to use the same free set as that chosen by *ARP*/*wARP* when run immediately after *ARP*/*wARP*. Dummy atoms were not removed unless *ARP*/*wARP* removed them, as they did not significantly affect *R*_free_.

In the next section, we deemed one pipeline or pipeline combination to be better than another when it produced an improvement of at least 5% in the relevant measure (completeness or *R*_free_); other improvement thresholds are reported in the supporting information. Execution time was not considered here, as this has been compared previously for the individual pipelines (Alharbi *et al.*, 2019 ▸).

## 4. Results   

### 4.1. Overview   

We present the results of our comparison using the pipeline and pipeline-combination identifiers defined in Table 1 ▸. Table 2 ▸ shows the number of ‘complete’, ‘intermediate’ and ‘failed’ data sets for each of the pipeline variants (*i.e.* pipelines and pipeline combinations) that we used in our experiments. The data sets were marked as ‘intermediate’ either when the 48-hour time limit was reached while the pipeline was still executing or when the pipeline stopped/crashed before building the final structure. Data sets for which no structure was built were marked as ‘failed’, and this occurred when the time limit was reached before the pipeline built an intermediate model.

As shown in Table 2 ▸, structures were successfully built for most of the data sets; the pipelines only failed to build six data sets (original and synthetic data sets) out of a total of 1211. After omitting the 52 data sets used in *Buccaneer* development (see Section 2) and the failed data sets, 148 (original) and 746 (synthetic) data sets were used in the analysis, representing 74% of the original and synthetic data sets.

Table 3 ▸ shows the mean and standard deviation (SD) of the structure completeness and *R*_free_ achieved for these data sets by each pipeline variant. The pipelines built structures with high completeness from the original data sets, the majority of which are at better than 2.5 Å resolution. The highest mean completeness was 94% with 11% SD (for *Phenix AutoBuild* followed by *Buccaneer*), compared with a lowest mean completeness of 78% with 33% SD (for *SHELXE* followed by *ARP*/*wARP*). The highest mean completeness decreased to 50% with 30% SD for the synthetic data sets, the resolutions of which range from 3.2 to 4.0 Å. From the original data sets, the pipelines built the structures with a mean *R*_free_ of between 0.26 and 0.33 and an SD of between 0.04 and 0.10. When building the structures from synthetic data sets, the mean *R*_free_ increased to between 0.38 and 0.52 with an SD of between 0.05 and 0.09.

### 4.2. Structure completeness   

**Figure 1:**
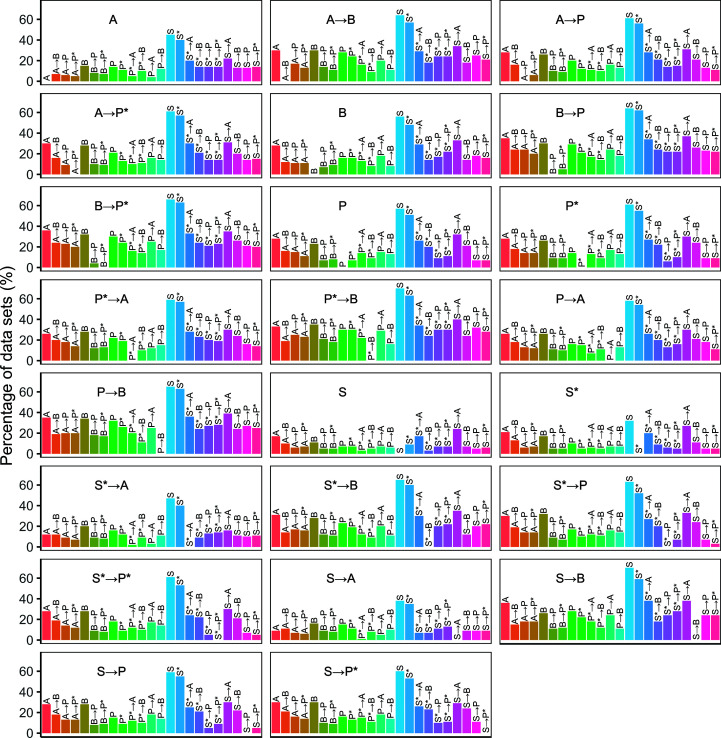
Structure-completeness comparison for the models generated from the original data sets. Each plot corresponds to a pipeline variant, and shows the percentage (rounded to the nearest integer) of structures that the pipeline variant built with at least 5% higher structure completeness than each of the other pipeline variants.

Fig. 1 ▸ shows the structure-completeness results for the original resolution data sets. Running the pipelines in pairwise combinations shows significant improvements compared with running a single pipeline. For example, both *Phenix AutoBuild* post-*ARP*/*wARP* and *Buccaneer* post-*ARP*/*wARP* achieved at least a 5% higher structure completeness than *ARP*/*wARP* alone for 28% or more of the data sets; in contrast, *ARP*/*wARP* on its own was better than the two-pipeline combinations for only 6% and 7% of the data sets, respectively. Similarly, running *Phenix AutoBuild* after *Buccaneer* increased the completeness for 30% of the data sets compared with running *Buccaneer* on its own, while *Buccaneer* alone was only better than this pipeline combination for 7% of the data sets.

Running *Phenix AutoBuild* in combination with *Buccaneer* led to higher completeness than using *ARP*/*wARP* after or before *Phenix AutoBuild*. Using *Buccaneer* to build an initial structure for *Phenix AutoBuild* resulted in completeness improvements (of at least 5%) for 24% of the data sets, compared with only 10% when *ARP*/*wARP* was used to build an initial model. These results decreased slightly to 20% and 9%, respectively, when *Parrot* was used before *Phenix AutoBuild*.

It is interesting to consider the extent to which the pairwise combination of pipelines produces a better model compared with running both of the component pipelines and picking the best result; this allows us to distinguish between the case where the second pipeline simply conserves the good features of the first and that where the pipelines have complementary features which can augment one another. Table 4 ▸ shows the percentage of the original and synthetic data sets that are built with least 5% higher structure completeness by the combined pipelines or either of the two pipelines alone. Running *Phenix AutoBuild* alone built the structures with higher completeness compared with when *ARP*/*wARP* had been run before it: 11% and 49% of the original and synthetic data sets, respectively, were built with higher completeness by *Phenix AutoBuild* alone, compared with 8% and 10% of the original and synthetic data sets, respectively, when *ARP*/*wARP* was run in combination with *Phenix AutoBuild*. However, *Buccaneer* with *Phenix AutoBuild* showed greater benefits; only 2% and 11% of *Buccaneer* models built from the original and synthetic data sets, respectively, are better in terms of structure completeness, compared with 14% and 41% of both data sets built with higher completeness when *Phenix AutoBuild* ran after *Buccaneer*.

**Figure 2:**
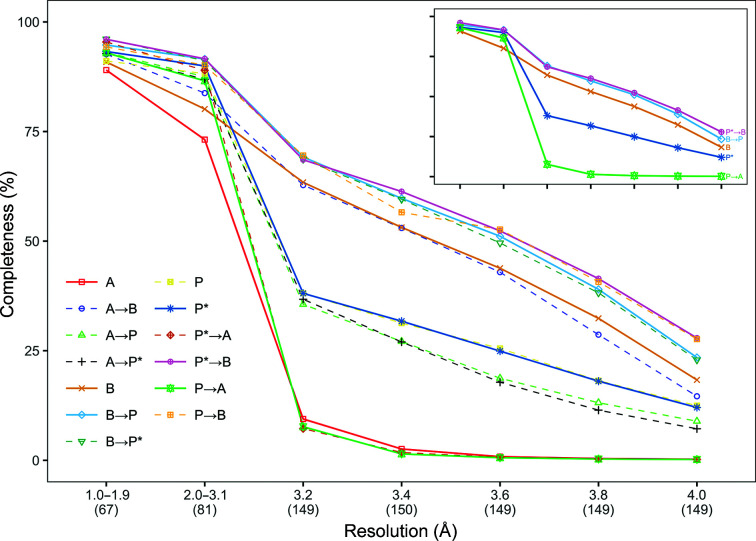
Mean completeness for the protein models built for all data sets. The data sets are grouped into bins based on their resolution, with the number of data sets in each bin shown in parentheses under the graph.

Fig. 2 ▸ shows the mean completeness for both the original and synthetic data sets. Combined pipelines outperformed individual pipelines at resolutions of 1.0–1.9 Å, and *Buccaneer* post-*Phenix AutoBuild* with *Parrot* outperformed the other pipeline variants at resolutions worse than 3.1 Å. *Phenix AutoBuild* after *Buccaneer* obtained close results at resolutions worse than 3.1 Å and *ARP*/*wARP* combined with *Phenix AutoBuild* performed poorly at these resolutions.

**Figure 3:**
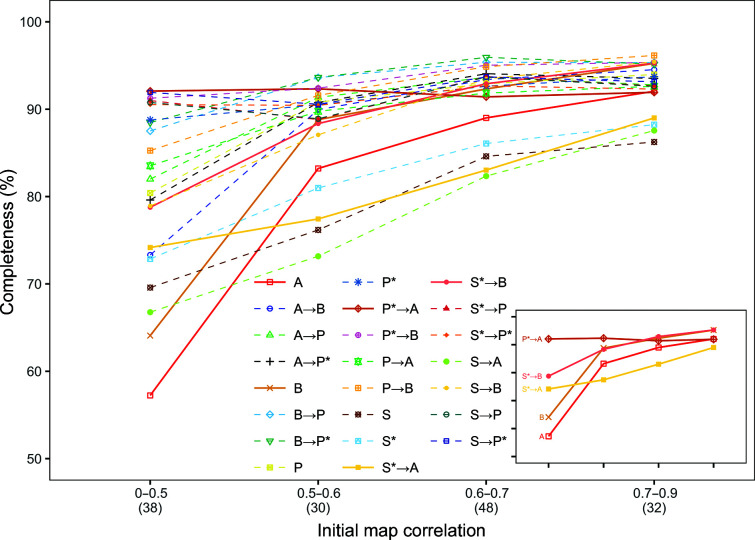
Mean completeness for the models built for the original data sets, grouped into bins based on their initial map correlation (*F*-map correlation); the number of data sets in each bin is reported in parentheses under the graph.

Fig. 3 ▸ shows how the mean completeness varied with the mean initial map correlation (*F*-map) for the original data sets. *ARP*/*wARP* running after *Phenix AutoBuild* with *Parrot* at an initial map correlation lower than 0.5 led to greater than 90% completeness, compared with running *ARP*/*wARP* on its own, which achieved less than 60% completeness. When the initial phases are better, the majority of the pipeline results reach greater than 90% completeness at initial map correlations of between 0.7 and 0.9.

**Figure 4:**
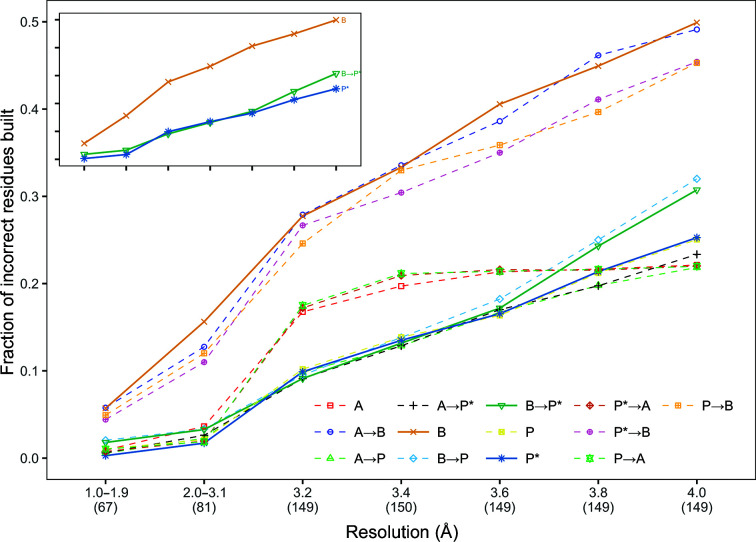
Mean fraction of residues incorrectly built in the protein models built for all data sets. The data sets are grouped into bins based on their resolution, with the number of data sets in each bin shown in parentheses under the graph. The number of residues incorrectly built was normalized by dividing it by the number of residues in the deposited model.

Fig. 4 ▸ shows the fraction of incorrect residues that were built for both the original and synthetic data sets. Compared with other pipelines, a known problem of using *Buccaneer* is that it may build a large number of incorrect residues, which can be 50% of the structure at 4.0 Å resolution. *Phenix AutoBuild* outperformed *Buccaneer* in decreasing the number of incorrect residues, and using *Phenix AutoBuild* post-*Buccaneer* reduced junk residues to around 30% of the structure at 4.0 Å resolution.

**Figure 5:**
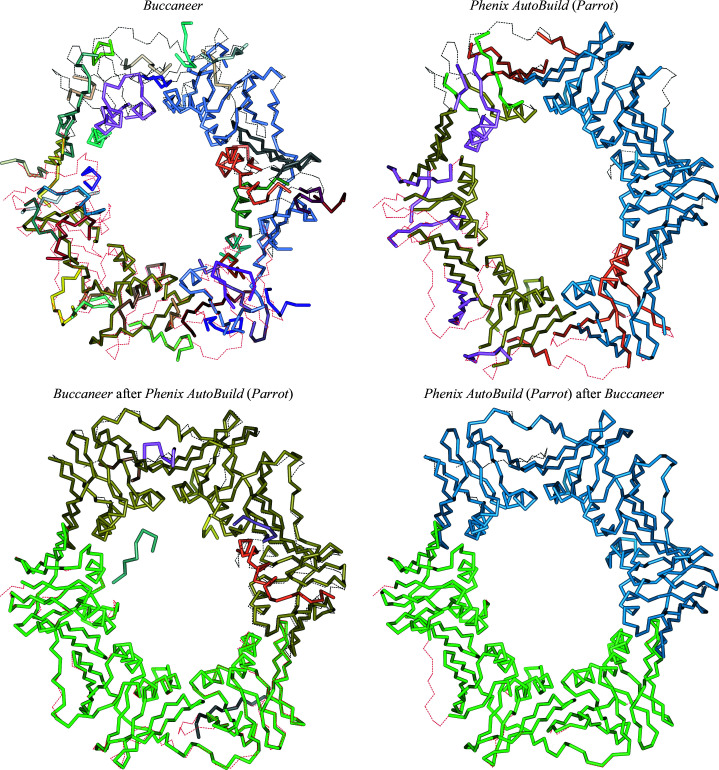
Four structures built by *Buccaneer*, *Phenix AutoBuild* (*Parrot*) and their combinations, and comparison with the deposited structures. The chains of deposited structures are coloured with red and black bonds. The PDB code is 2awa and its resolution is 2.7 Å.

Fig. 5 ▸ provides an illustration of a case in which pairwise running of two pipelines gave substantially better results than either pipeline alone, in this case PDB entry 2awa. The *Buccaneer* model is substantially incomplete, with some correctly traced fragments but with only 8% of the sequence correctly docked. The *Phenix AutoBuild* model is more complete, but still only 59% of the sequence is correctly docked. When both pipelines are used, a largely complete model is obtained and correctly sequenced. Running *Phenix AutoBuild* with *Parrot* after *Buccaneer* built a structure with a higher complete­ness of 91%.

### 4.3. *R*_free_   

**Figure 6:**
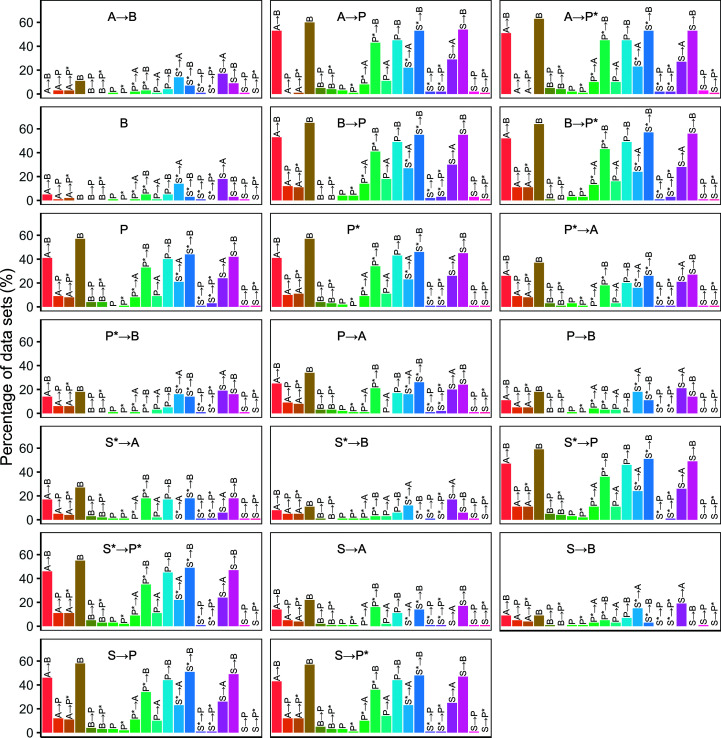
Comparison of *R*_free_ (rounded to two decimal places) for the structures generated from the original data sets. Each plot shows the percentage of models that a pipeline variant built with an *R*_free_ at least 5% lower than each other pipeline variant.

Fig. 6 ▸ shows the *R*_free_ results for the original resolution data sets. Similar to the completeness comparison in Section 4.2, the individual pipelines performed worse than when we used them in combination with other pipelines. Comparing *Buccaneer* on its own with the combination in which it was followed by *Phenix AutoBuild* shows significant improvement on including *Phenix AutoBuild*, as the structures produced for 65% of the data sets decreased (by at least 5%) in *R*_free_ when *Phenix AutoBuild* ran after *Buccaneer*. None of the structures built by *Buccaneer* on its own was better in *R*_free_ than those built by *Phenix AutoBuild* after *Buccaneer*.

Finalizing the structures using *Buccaneer* as the second pipeline of a pipeline combination caused high *R*_free_, while starting with a *Buccaneer* structure as an initial model for other pipelines was more effective. As shown in Table 4 ▸, using *Buccaneer* after *Phenix AutoBuild* did not improve *R*_free_ compared with *Phenix AutoBuild* alone, as 36% of the original data sets have a lower *R*_free_. Running *Phenix AutoBuild* after *Buccaneer* improved 4% of the original data sets in terms of *R*_free_, and no *Buccaneer* models had a lower *R*_free_ than the combination. Following *Phenix AutoBuild* by *ARP*/*wARP* generated better results than using *Buccaneer* after *Phenix AutoBuild*. *ARP*/*wARP* built 17% of the data sets with a better *R*_free_ than *Buccaneer*, while only 3% were better built by *Buccaneer* compared with *ARP*/*wARP*.

**Figure 7:**
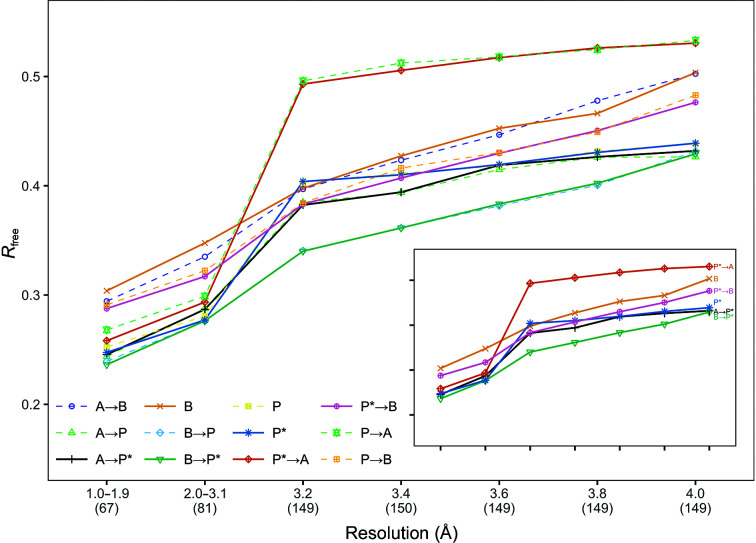
Mean protein model *R*_free_ for the data sets partitioned into classes based on their resolution. The number of data sets in each class is indicated in parentheses under the graph.

Fig. 7 ▸ shows the mean *R*_free_ for the data sets grouped into classes based on their resolution. Running *Phenix AutoBuild* with *Parrot* after *ARP*/*wARP* or *Buccaneer* led to a lower *R*_free_ at resolutions better than 1.9 Å compared with *Buccaneer* or *ARP*/*wARP* run after *Phenix AutoBuild*. The combination of *Buccaneer* and *Phenix AutoBuild* achieved the lowest *R*_free_ across all pipeline combinations at resolutions worse than 3.1 Å, while *ARP*/*wARP* after *Phenix AutoBuild* achieved the highest *R*_free_ for the same resolution range.

## 5. Discussion   

We have presented the pairwise running of widely used model-building pipelines using the original and simulated lower resolution data sets and have focused on the successful combinations. We have focused on the results of running pipelines in sequence with at most minor adjustments to the pipeline options; however, in the future it may be possible to produce further improvements by the deeper integration of methods from different pipelines.

Combining the pipelines improved the structure built by the first pipeline in most of the data sets. The significance of the improvement depended on the limitations of the first pipeline and the ability of the second pipeline to address these limitations. Running *Buccaneer* after *Phenix AutoBuild* improved the structure completeness at resolutions worse than 3.1 Å, as it is known that *Phenix AutoBuild* is more effective at resolutions better than 3.0 Å. Running the same two pipelines in the reverse order yielded better results than either pipeline because *Phenix AutoBuild* was able to address poor finalization of the model by *Buccaneer*, leading to improved *R* factors.

When we compared the structure completeness on the basis of the initial map correlation, few pipeline combinations performed well when the initial phases were poor. *ARP*/*wARP* after *Phenix AutoBuild* obtained the best results when *Phenix AutoBuild* ran after *Parrot*. Also, *Phenix AutoBuild* after *SHELXE* and *Buccaneer* after *Phenix AutoBuild* with *Parrot* obtained close results. We notice from these combinations that the pipelines that perform density modification internally during model building produced a good structure for others to use as an initial structure. For example, *Buccaneer* after *SHELXE* showed better results than *Buccaneer* alone, as *SHELXE* contributes substantially to phase quality and the performance of *Buccaneer* is affected by the quality of the phases.

When comparing *R*_free_, most of the pipeline variants achieved a close *R*_free_ at resolutions better than 3.1 Å, and *Phenix AutoBuild* run after *Buccaneer* outperformed the others at resolutions worse than 3.1 Å. *ARP*/*wARP* run after *Phenix AutoBuild* and *Buccaneer* run after *ARP*/*wARP* were the worst combinations at resolutions worse than 3.1 Å, as they produced structures with the highest mean *R*_free_ values.

The results of our comparison show the usefulness of pipeline combinations instead of running them individually. Pairwise pipeline combinations have the ability to fix errors caused by the first pipeline in the combination. For instance, *Buccaneer* alone often produced a highly complete structure but with a large number of incorrect residues owing to its building method. In contrast, when *Buccaneer* was followed by *Phenix AutoBuild*, the number of incorrect residues significantly decreased because of the ability of *Phenix AutoBuild* to fix the structure without adding new residues. The pipelines that do not perform density modification as part of model building (for example *ARP*/*wARP* and *Buccaneer*) showed the worst results against the initial map correlation (correlation of <0.5). Therefore, combining *ARP*/*wARP* and *Buccaneer* with *Phenix AutoBuild* produced a more complete structure than that generated by either *ARP*/*wARP* or *Buccaneer* alone, both when *Phenix AutoBuild* was used on its own or with *Parrot*. The performance of the pipelines might be biased owing to our approach in truncating the data sets to lower resolution, as explained in detail in our recent work (Alharbi *et al.*, 2019 ▸); however, this was necessary owing to the difficulty of obtaining large real data sets.

The decision on which pipeline to start with depends on the quality of the electron-density map. When the initial phases are not good, starting with a pipeline that includes density modification is the most effective approach. However, the decision can change from one structure to another, even if the structural features are very similar. Running all of these pipeline variants can be time-consuming, and there is not one individual or combined pipeline that is the best across all resolution ranges. Developers are inevitably influenced by their own interests and by the coverage of their test data sets. Combining features from different model-building pipelines improves the model-building results because in many cases the complementary features of models from different pipelines are preserved. Further efforts to understand the strengths and weaknesses of different tools may allow further improvements through a more systematic approach to combining components from different pipeline. Moreover, further research is required to provide users with clear guidelines as to which individual pipeline or combined pipeline is the best depending on their model features.

## 6. Data and methods   

The structures built by the pipeline combinations and the log files are available at https://doi.org/10.15124/4b7c880a-d6b0-471a-a379-d52c4ee947fe.

## Supplementary Material

Appendices A–E. DOI: 10.1107/S2059798320010542/qj5002sup1.pdf

Pairwise running of automated crystallographic model-building pipelines.: https://doi.org/10.15124/4b7c880a-d6b0-471a-a379-d52c4ee947fe

## Figures and Tables

**Table 1:** The pipeline and pipeline-combination identifiers (IDs) used to present the results

ID	Description
A	*ARP*/*wARP*
B	*Buccaneer* in *CCP*4*i* using five iterations
P	*Phenix AutoBuild*
P*	*Phenix AutoBuild* with *Parrot*
S	*SHELXE*
S*	*SHELXE* with *Parrot*
*x*→*y*	Pairwise pipeline combination, with pipeline *y* executed after pipeline *x*; for example, A→P* denotes a pairwise combination in which *Phenix AutoBuild* with *Parrot* is run after *ARP*/*wARP*

**Table 2:** Complete and intermediate models produced by the 23 pipeline variants for the original and synthetic resolution data sets, where ‘(T)’ and ‘(C)’ denote intermediate models produced by pipeline executions that timed out and crashed, respectively Models used in the comparison: 148 original and 746 synthetic.

	Original			Synthetic		
Pipeline variant	Complete	Intermediate	Failed	Complete	Intermediate	Failed
A	202	0(T) 0(C)	0	1008	1(T) 0(C)	0
A→P*	201	1(T) 0(C)	0	1007	2(T) 0(C)	0
A→B	202	0(T) 0(C)	0	1009	0(T) 0(C)	0
B	202	0(T) 0(C)	0	1009	0(T) 0(C)	0
B→P*	197	4(T) 0(C)	1	1005	0(T) 0(C)	4
P*	199	1(T) 1(C)	1	1001	8(T) 0(C)	0
P*→A	200	1(T) 0(C)	1	1008	1(T) 0(C)	0
P*→B	201	0(T) 0(C)	1	1009	0(T) 0(C)	0
S*	200	2(T) 0(C)	0	—	—	—
S*→A	202	0(T) 0(C)	0	—	—	—
S*→B	202	0(T) 0(C)	0	—	—	—
S*→P*	196	4(T) 0(C)	2	—	—	—
A→P	199	2(T) 0(C)	1	1009	0(T) 0(C)	0
B→P	200	0(T) 0(C)	2	1003	2(T) 0(C)	4
P	199	1(T) 0(C)	2	1001	7(T) 0(C)	1
P→A	200	0(T) 0(C)	2	1002	6(T) 0(C)	1
P→B	200	0(T) 0(C)	2	1008	0(T) 0(C)	1
S	200	2(T) 0(C)	0	—	—	—
S→A	202	0(T) 0(C)	0	—	—	—
S→B	202	0(T) 0(C)	0	—	—	—
S*→P	197	3(T) 0(C)	2	—	—	—
S→P*	198	2(T) 0(C)	2	—	—	—
S→P	197	3(T) 0(C)	2	—	—	—

**Table:** Original data sets.

	Completeness (%)		*R*_free_	
Pipeline variant	Mean	SD	Mean	SD
P*→B	94	11	0.30	0.04
B→P*	93	8	0.26	0.04
B→P	93	10	0.26	0.04
S→P*	92	7	0.26	0.04
S*→P*	92	9	0.26	0.04
S*→P	92	9	0.26	0.04
S→P	92	9	0.26	0.04
P*→A	92	11	0.28	0.04
P→B	92	14	0.31	0.05
P*	91	10	0.26	0.04
P	90	15	0.27	0.05
A→P	90	16	0.27	0.06
A→P*	90	17	0.27	0.06
P→A	89	17	0.28	0.06
S→B	89	18	0.32	0.06
S*→B	89	18	0.32	0.06
A→B	88	22	0.32	0.06
B	85	23	0.33	0.07
S*	82	18	—	—
S*→A	81	31	0.30	0.09
A	80	30	—	—
S	79	21	—	—
S→A	78	33	0.31	0.10

**Table:** Synthetic data sets.

	Completeness (%)		*R*_free_	
Pipeline variant	Mean	SD	Mean	SD
P*→B	50	30	0.43	0.08
B→P	49	29	0.38	0.07
P→B	49	30	0.43	0.08
B→P*	48	29	0.38	0.07
B	42	31	0.45	0.08
A→B	40	32	0.45	0.09
P*	25	16	0.42	0.05
P	25	16	0.42	0.05
A→P	21	18	0.41	0.08
A→P*	20	18	0.41	0.08
A	3	9	—	—
P*→A	2	8	0.51	0.06
P→A	2	8	0.52	0.06

**Table 4:** Structure completeness and *R*_free_ comparison for the original and synthetic data sets, indicating how often pairwise running outperforms either of the component pipelines Each row corresponds to a pipeline variant and shows the percentage (rounded to the nearest integer) of the models where either the combined pipeline (*x*→*y*) or the individual pipelines alone (*x* or *y*) built structures with at least 5% higher completeness and lower *R*_free_.

	Original						Synthetic					
	Completeness			*R*_free_			Completeness			*R*_free_		
Pipeline variant	*x*→*y*	*x*	*y*	*x*→*y*	*x*	*y*	*x*→*y*	*x*	*y*	*x*→*y*	*x*	*y*
A→B	14	3	8	—	—	—	27	0	33	—	—	—
A→P*	6	3	11	—	—	—	12	1	50	—	—	—
A→P	8	4	11	—	—	—	10	0	49	—	—	—
B→P*	9	3	5	3	0	2	40	14	4	30	1	4
B→P	14	2	2	4	0	3	41	11	2	29	1	4
P*→A	6	11	1	—	—	—	1	91	1	—	—	—
P*→B	14	3	2	0	29	0	47	7	17	9	23	4
P→A	6	12	3	—	—	—	0	91	1	—	—	—
P→B	17	7	3	0	36	0	42	7	18	8	24	5
S→A	6	11	16	—	—	—	—	—	—	—	—	—
S→B	22	4	11	—	—	—	—	—	—	—	—	—
S→P*	9	4	8	—	—	—	—	—	—	—	—	—
S→P	13	4	7	—	—	—	—	—	—	—	—	—
S*→A	7	13	9	—	—	—	—	—	—	—	—	—
S*→B	21	6	11	—	—	—	—	—	—	—	—	—
S*→P*	5	3	7	—	—	—	—	—	—	—	—	—
S*→P	12	5	7	—	—	—	—	—	—	—	—	—
