# Supplementary material for: Pairwise running of automated crystallographic model-building pipelines
Source: Acta Crystallogr D Struct Biol. 2020 Aug 19;76(Pt 9):814–23. doi: 10.1107/S2059798320010542 (PMC7466752; doi:10.1107/S2059798320010542)
Supplement: Supplementary file 1 [file d-76-00814-sup1.pdf]

Appendix A
The Results of the Original data sets without the Buccaneer Development Data sets

Table 1. *Complete and intermediate models produced by the 23 pipeline variants for the original data sets, where '(T)' and '(C)' denote intermediate models produced by pipeline executions that timed out and crashed, respectively.*

Pipeline variant	HA-NCS			MR-NCS			NO-NCS		
	Complete	Intermediate	Failed	Complete	Intermediate	Failed	Complete	Intermediate	Failed
A	201	1(T) 0(C)	0	202	0(T) 0(C)	0	202	0(T) 0(C)	0
A→P^*	196	3(T) 0(C)	3	197	2(T) 0(C)	3	201	1(T) 0(C)	0
A→B	202	0(T) 0(C)	0	202	0(T) 0(C)	0	202	0(T) 0(C)	0
B	202	0(T) 0(C)	0	202	0(T) 0(C)	0	202	0(T) 0(C)	0
B→P^*	200	0(T) 0(C)	2	197	3(T) 0(C)	2	197	4(T) 0(C)	1
P^*	198	2(T) 1(C)	1	200	0(T) 1(C)	1	199	1(T) 1(C)	1
P^*⟶A	201	0(T) 0(C)	1	201	0(T) 0(C)	1	200	1(T) 0(C)	1
P^*⟶B	201	0(T) 0(C)	1	201	0(T) 0(C)	1	201	0(T) 0(C)	1
S^*	202	0(T) 0(C)	0	201	1(T) 0(C)	0	200	2(T) 0(C)	0
S^*⟶A	202	0(T) 0(C)	0	202	0(T) 0(C)	0	202	0(T) 0(C)	0
S^*⟶B	202	0(T) 0(C)	0	202	0(T) 0(C)	0	202	0(T) 0(C)	0
S^*⟶P^*	198	2(T) 0(C)	2	197	3(T) 0(C)	2	196	4(T) 0(C)	2
A→P	-	-	-	-	-	-	199	2(T) 0(C)	1
B→P	-	-	-	-	-	-	200	0(T) 0(C)	2
P	-	-	-	-	-	-	199	1(T) 0(C)	2
P→A	-	-	-	-	-	-	200	0(T) 0(C)	2
P→B	-	-	-	-	-	-	200	0(T) 0(C)	2
S	-	-	-	-	-	-	200	2(T) 0(C)	0
S→A	-	-	-	-	-	-	202	0(T) 0(C)	0
S→B	-	-	-	-	-	-	202	0(T) 0(C)	0
S^*⟶P	-	-	-	-	-	-	197	3(T) 0(C)	2
S⟶P^*	-	-	-	-	-	-	198	2(T) 0(C)	2
S→P	-	-	-	-	-	-	197	3(T) 0(C)	2

Models used in the comparison: 147 HA-NCS, 147 MR-NCS and 148 NO-NCS.

Table 2. *Structure completeness comparison for the models generated from the original HA-NCS data sets. Each row corresponds to a pipeline variant, and shows the percentage (rounded to the nearest integer) of models that the pipeline variant built with higher structure completeness than each of the other pipeline variants.*

Pipeline variant	A	A$\to P^*$	A$\to B$	B	B$\to P^*$	P^*	$P^*\longrightarrow A$	$P^*\longrightarrow B$	S^*	$S^*\longrightarrow A$	$S^*\longrightarrow B$	$S^*\longrightarrow P^*$
A	0	25	15	29	18	41	28	13	65	43	20	38
A$\to P^*$	53	0	22	45	22	45	41	19	76	52	31	44
A$\to B$	78	63	0	61	41	66	68	33	81	77	53	65
B	63	44	17	0	16	50	49	18	73	62	34	49
B$\to P^*$	71	61	35	57	0	65	67	35	87	69	48	67
P^*	48	39	27	41	21	0	39	16	75	51	31	38
$P^*\longrightarrow A$	46	34	24	40	24	49	0	18	77	47	30	45
$P^*\longrightarrow B$	78	73	45	63	49	76	73	0	87	77	56	72
S^*	31	20	16	20	9	20	17	10	0	33	12	20
$S^*\longrightarrow A$	32	24	14	29	19	37	23	15	62	0	23	36
$S^*\longrightarrow B$	70	59	27	47	32	60	59	23	84	67	0	59
$S^*\longrightarrow P^*$	50	32	23	40	20	38	35	15	72	49	30	0

0 87

Table 3. *Structure completeness comparison for the models generated from the original HA-NCS data sets. Each row corresponds to a pipeline variant, and shows the percentage (rounded to the nearest integer) of models that the pipeline variant built with equal structure completeness to each of the other pipeline variants.*

Pipeline variant	A	A$\to P^*$	A$\to B$	B	B$\to P^*$	P^*	$P^*\longrightarrow A$	$P^*\longrightarrow B$	S^*	$S^*\longrightarrow A$	$S^*\longrightarrow B$	$S^*\longrightarrow P^*$
A	100	22	7	8	11	12	27	9	5	25	10	12
A$\to P^*$	22	100	16	12	17	16	24	8	5	24	10	24
A$\to B$	7	16	100	22	24	7	7	22	3	9	20	12
B	8	12	22	100	27	9	11	19	6	9	19	11
B$\to P^*$	11	17	24	27	100	14	9	16	4	12	20	13
P^*	12	16	7	9	14	100	12	8	5	12	10	24
$P^*\longrightarrow A$	27	24	7	11	9	12	100	9	6	30	11	20
$P^*\longrightarrow B$	9	8	22	19	16	8	9	100	3	8	21	13
S^*	5	5	3	6	4	5	6	3	100	5	3	8
$S^*\longrightarrow A$	25	24	9	9	12	12	30	8	5	100	10	15
$S^*\longrightarrow B$	10	10	20	19	20	10	11	21	3	10	100	11
$S^*\longrightarrow P^*$	12	24	12	11	13	24	20	13	8	15	11	100

3 100

Table 4. *Structure completeness comparison for the models generated from the original HA-NCS data sets. Each row corresponds to a pipeline variant, and shows the percentage (rounded to the nearest integer) of models that the pipeline variant built with at least 5% higher structure completeness than each of the other pipeline variants.*

Pipeline variant	A	A→P^*	A→B	B	B→P^*	P^*	$P^*\longrightarrow A$	$P^*\longrightarrow B$	S^*	$S^*\longrightarrow A$	$S^*\longrightarrow B$	$S^*\longrightarrow P^*$
A	0	7	8	13	7	14	5	7	41	24	12	13
A→P^*	28	0	14	22	4	14	14	8	52	31	16	15
A→B	33	18	0	25	11	25	24	8	60	35	14	30
B	28	14	11	0	7	22	17	5	52	32	10	22
B→P^*	35	20	20	27	0	29	24	10	64	37	20	24
P^*	27	12	15	23	3	0	15	7	49	29	18	12
$P^*\longrightarrow A$	21	14	17	24	10	17	0	8	52	30	17	16
$P^*\longrightarrow B$	39	26	18	29	16	33	29	0	65	38	20	35
S^*	18	6	12	12	3	8	5	3	0	22	9	4
$S^*\longrightarrow A$	10	7	10	14	6	14	1	6	34	0	8	13
$S^*\longrightarrow B$	33	19	12	25	10	25	22	6	59	35	0	24
$S^*\longrightarrow P^*$	27	12	18	25	4	10	16	7	51	31	17	0

0 65

Table 5. *Structure completeness comparison for the models generated from the original HA-NCS data sets. Each row corresponds to a pipeline variant, and shows the percentage (rounded to the nearest integer) of models that the pipeline variant built with between 1% and 4% higher structure completeness than each of the other pipeline variants.*

Pipeline variant	A	A→P^*	A→B	B	B→P^*	P^*	$P^*\longrightarrow A$	$P^*\longrightarrow B$	S^*	$S^*\longrightarrow A$	$S^*\longrightarrow B$	$S^*\longrightarrow P^*$
A	0	18	7	16	12	27	22	6	24	19	8	25
A→P^*	25	0	8	23	18	31	27	11	24	22	15	29
A→B	45	44	0	35	30	41	44	25	21	41	39	35
B	35	30	6	0	10	29	32	13	22	30	24	27
B→P^*	35	41	16	30	0	37	44	25	23	33	28	43
P^*	20	27	12	18	18	0	24	9	26	22	13	26
$P^*\longrightarrow A$	24	20	7	16	14	32	0	10	25	17	13	29
$P^*\longrightarrow B$	39	47	27	33	33	43	44	0	22	39	36	37
S^*	12	14	4	9	5	12	12	7	0	11	3	16
$S^*\longrightarrow A$	22	16	4	16	13	24	22	9	28	0	15	23
$S^*\longrightarrow B$	37	39	14	22	22	35	37	17	25	32	0	35
$S^*\longrightarrow P^*$	22	20	5	15	16	28	19	7	21	18	13	0

0 47

Table 6. *Comparison of R-work/R-free (rounded to two decimal places) for the models generated from the original HA-NCS data sets. Each row shows the percentage of models that a pipeline variant built with lower R-work or R-free than each other pipeline variant.*

Pipeline variant	A	A→P^*	A→B	B	B→P^*	P^*	P^*→A	P^*→B	S^*	S^*→A	S^*→B	S^*→P^*
A $_{R-work}$	0	24	86	93	24	33	20	86	100	33	91	33
A $_{R-free}$	-	-	-	-	-	-	-	-	-	-	-	-
A→P^* $_{R-work}$	64	0	94	98	34	56	54	89	100	61	95	56
A→P^* $_{R-free}$	-	0	90	92	33	48	77	81	-	84	88	50
A→B $_{R-work}$	9	3	0	51	2	2	2	26	98	5	46	3
A→B $_{R-free}$	-	5	0	52	4	1	15	31	-	28	46	4
B $_{R-work}$	5	1	16	0	1	3	1	11	99	3	27	2
B $_{R-free}$	-	5	22	0	2	3	11	18	-	25	28	3
B→P^* $_{R-work}$	62	35	96	99	0	56	54	96	100	61	97	59
B→P^* $_{R-free}$	-	40	93	95	0	48	84	95	-	85	94	52
P^* $_{R-work}$	44	16	93	95	19	0	31	93	99	40	95	25
P^* $_{R-free}$	-	31	95	95	24	0	73	93	-	75	95	33
P^*→A $_{R-work}$	43	29	94	97	27	44	0	93	100	35	95	42
P^*→A $_{R-free}$	-	17	75	84	12	16	0	72	-	48	79	12
P^*→B $_{R-work}$	10	5	30	56	3	3	3	0	99	10	45	5
P^*→B $_{R-free}$	-	12	39	58	3	2	18	0	-	29	45	6
S^* $_{R-work}$	0	0	2	1	0	1	0	1	0	0	0	0
S^* $_{R-free}$	-	-	-	-	-	-	-	-	-	-	-	-
S^*→A $_{R-work}$	34	23	90	95	19	38	16	86	100	0	93	37
S^*→A $_{R-free}$	-	11	63	68	9	12	21	63	-	0	64	10
S^*→B $_{R-work}$	6	2	20	41	2	3	2	15	100	4	0	2
S^*→B $_{R-free}$	-	7	22	47	3	3	12	20	-	29	0	4
S^*→P^* $_{R-work}$	46	15	94	95	16	26	34	91	100	41	94	0
S^*→P^* $_{R-free}$	-	28	90	93	21	31	73	91	-	79	95	0

0 100

Table 7. *Comparison of R-work/R-free (rounded to two decimal places) for the models generated from the original HA-NCS data sets. Each row shows the percentage of models that a pipeline variant built with equal R-work or R-free to each other pipeline variant.*

Pipeline variant	A	A→P^*	A→B	B	B→P^*	P^*	P^*→A	P^*→B	S^*	S^*→A	S^*→B	S^*→P^*
A $_{R-work}$	100	12	5	2	14	23	37	3	0	33	3	20
A $_{R-free}$	-	-	-	-	-	-	-	-	-	-	-	-
A→P^* $_{R-work}$	12	100	3	1	31	29	17	6	0	16	3	29
A→P^* $_{R-free}$	-	100	5	3	27	21	6	7	-	5	4	22
A→B $_{R-work}$	5	3	100	33	2	5	4	44	0	5	33	3
A→B $_{R-free}$	-	5	100	25	3	4	10	29	-	10	32	5
B $_{R-work}$	2	1	33	100	0	1	3	33	0	2	33	3
B $_{R-free}$	-	3	25	100	3	3	5	24	-	7	25	3
B→P^* $_{R-work}$	14	31	2	0	100	25	18	1	0	20	1	25
B→P^* $_{R-free}$	-	27	3	3	100	28	5	3	-	6	3	27
P^* $_{R-work}$	23	29	5	1	25	100	25	4	0	22	3	49
P^* $_{R-free}$	-	21	4	3	28	100	12	5	-	13	3	37
P^*→A $_{R-work}$	37	17	4	3	18	25	100	4	0	48	3	24
P^*→A $_{R-free}$	-	6	10	5	5	12	100	10	-	31	9	14
P^*→B $_{R-work}$	3	6	44	33	1	4	4	100	0	4	40	4
P^*→B $_{R-free}$	-	7	29	24	3	5	10	100	-	8	35	3
S^* $_{R-work}$	0	0	0	0	0	0	0	0	100	0	0	0
S^* $_{R-free}$	-	-	-	-	-	-	-	-	-	-	-	-
S^*→A $_{R-work}$	33	16	5	2	20	22	48	4	0	100	3	22
S^*→A $_{R-free}$	-	5	10	7	6	13	31	8	-	100	7	12
S^*→B $_{R-work}$	3	3	33	33	1	3	3	40	0	3	100	4
S^*→B $_{R-free}$	-	4	32	25	3	3	9	35	-	7	100	1
S^*→P^* $_{R-work}$	20	29	3	3	25	49	24	4	0	22	4	100
S^*→P^* $_{R-free}$	-	22	5	3	27	37	14	3	-	12	1	100

0 100

Table 8. *Comparison of R-work/R-free (rounded to two decimal places) for the models generated from the original HA-NCS data sets. Each row shows the percentage of models that a pipeline variant built with R-work or R-free at least 5% lower than each other pipeline variant.*

Pipeline variant	A	A→P^*	A→B	B	B→P^*	P^*	P^*→A	P^*→B	S^*	S^*→A	S^*→B	S^*→P^*
A $_{R-work}$	0	6	38	48	2	4	1	27	100	6	39	3
A $_{R-free}$	-	-	-	-	-	-	-	-	-	-	-	-
A→P^* $_{R-work}$	4	0	53	65	2	3	2	42	100	5	59	0
A→P^* $_{R-free}$	-	0	48	59	2	1	10	39	-	25	52	0
A→B $_{R-work}$	1	0	0	7	0	0	0	3	94	1	6	0
A→B $_{R-free}$	-	3	0	8	1	0	2	3	-	15	7	0
B $_{R-work}$	0	0	4	0	0	0	0	3	95	1	5	0
B $_{R-free}$	-	2	4	0	0	0	2	4	-	14	5	0
B→P^* $_{R-work}$	5	5	52	63	0	3	2	41	100	7	53	0
B→P^* $_{R-free}$	-	8	54	62	0	3	12	40	-	27	55	1
P^* $_{R-work}$	4	3	39	50	1	0	0	27	99	6	41	0
P^* $_{R-free}$	-	9	40	50	1	0	12	28	-	25	40	0
P^*→A $_{R-work}$	6	8	39	53	3	5	0	32	100	7	42	5
P^*→A $_{R-free}$	-	7	22	31	1	1	0	17	-	16	26	0
P^*→B $_{R-work}$	1	1	12	14	0	0	0	0	99	3	13	0
P^*→B $_{R-free}$	-	5	12	14	1	0	2	0	-	16	14	0
S^* $_{R-work}$	0	0	1	1	0	0	0	1	0	0	0	0
S^* $_{R-free}$	-	-	-	-	-	-	-	-	-	-	-	-
S^*→A $_{R-work}$	6	5	38	50	2	3	3	29	100	0	42	1
S^*→A $_{R-free}$	-	4	16	22	1	1	0	14	-	0	19	0
S^*→B $_{R-work}$	1	1	7	7	0	1	0	2	99	0	0	0
S^*→B $_{R-free}$	-	5	7	9	1	1	1	2	-	12	0	0
S^*→P^* $_{R-work}$	5	4	41	52	1	1	1	28	100	7	41	0
S^*→P^* $_{R-free}$	-	9	44	49	1	1	9	30	-	24	44	0

0 100

Table 9. *Comparison of R-work/R-free (rounded to two decimal places) for the models generated from the original HA-NCS data sets. Each row shows the percentage of models that a pipeline variant built with R-work or R-free between 1% and 4% lower than each other pipeline variant.*

Pipeline variant	A	A→P^*	A→B	B	B→P^*	P^*	P^*→A	P^*→B	S^*	S^*→A	S^*→B	S^*→P^*
A $_{R-work}$	0	18	48	45	22	29	18	60	0	27	52	30
A $_{R-free}$	-	-	-	-	-	-	-	-	-	-	-	-
A→P^* $_{R-work}$	60	0	41	33	32	53	52	47	0	56	36	56
A→P^* $_{R-free}$	-	0	42	33	31	47	67	42	-	59	36	50
A→B $_{R-work}$	8	3	0	44	2	2	2	23	4	4	40	3
A→B $_{R-free}$	-	3	0	44	3	1	13	29	-	13	39	4
B $_{R-work}$	5	1	12	0	1	3	1	7	4	3	21	2
B $_{R-free}$	-	3	18	0	2	3	9	14	-	12	23	3
B→P^* $_{R-work}$	56	31	44	36	0	53	52	55	0	54	44	59
B→P^* $_{R-free}$	-	32	39	33	0	46	72	54	-	58	39	52
P^* $_{R-work}$	40	12	53	46	18	0	31	65	0	34	53	25
P^* $_{R-free}$	-	22	54	45	23	0	61	65	-	50	54	33
P^*→A $_{R-work}$	37	21	55	44	24	38	0	61	0	29	53	37
P^*→A $_{R-free}$	-	10	52	52	11	14	0	55	-	32	53	12
P^*→B $_{R-work}$	9	4	18	42	3	3	3	0	0	7	32	5
P^*→B $_{R-free}$	-	6	28	44	2	2	16	0	-	13	31	6
S^* $_{R-work}$	0	0	1	0	0	1	0	0	0	0	0	0
S^* $_{R-free}$	-	-	-	-	-	-	-	-	-	-	-	-
S^*→A $_{R-work}$	28	18	52	45	17	35	14	58	0	0	50	35
S^*→A $_{R-free}$	-	7	46	46	8	12	21	49	-	0	45	10
S^*→B $_{R-work}$	5	1	14	33	2	2	2	13	1	4	0	2
S^*→B $_{R-free}$	-	3	16	38	2	2	11	18	-	17	0	4
S^*→P^* $_{R-work}$	41	11	53	42	15	24	33	63	0	33	52	0
S^*→P^* $_{R-free}$	-	19	47	44	20	29	65	61	-	55	50	0

0 72

Table 10. *Structure completeness comparison for the models generated from the original MR-NCS data sets. Each row corresponds to a pipeline variant, and shows the percentage (rounded to the nearest integer) of models that the pipeline variant built with higher structure completeness than each of the other pipeline variants.*

Pipeline variant	A	A→P^*	A→B	B	B→P^*	P^*	P^*→A	P^*→B	S^*	S^*→A	S^*→B	S^*→P^*
A	0	24	14	33	23	41	31	18	72	50	25	45
A→P^*	48	0	17	46	24	48	38	21	79	54	34	54
A→B	76	67	0	64	45	70	67	35	86	80	52	70
B	56	45	21	0	20	46	50	21	78	61	35	55
B→P^*	63	59	34	54	0	63	64	32	87	68	50	71
P^*	43	31	21	40	20	0	40	19	76	50	33	43
P^*→A	43	31	23	40	24	46	0	21	80	51	32	48
P^*→B	72	70	41	59	48	76	69	0	86	74	51	78
S^*	23	16	12	16	10	17	16	12	0	28	13	18
S^*→A	24	19	13	30	20	35	17	18	63	0	25	37
S^*→B	60	51	26	46	33	59	54	27	86	67	0	61
S^*→P^*	41	29	20	35	17	31	33	12	73	46	29	0

0 87

Table 11. *Structure completeness comparison for the models generated from the original MR-NCS data sets. Each row corresponds to a pipeline variant, and shows the percentage (rounded to the nearest integer) of models that the pipeline variant built with equal structure completeness to each of the other pipeline variants.*

Pipeline variant	A	A→P^*	A→B	B	B→P^*	P^*	P^*→A	P^*→B	S^*	S^*→A	S^*→B	S^*→P^*
A	100	28	11	10	14	16	27	10	5	26	15	14
A→P^*	28	100	16	9	16	20	31	9	5	27	15	16
A→B	11	16	100	15	21	9	10	24	2	7	22	10
B	10	9	15	100	25	14	10	20	6	10	19	10
B→P^*	14	16	21	25	100	17	12	20	3	12	18	12
P^*	16	20	9	14	17	100	14	5	7	16	7	27
P^*→A	27	31	10	10	12	14	100	10	4	32	14	18
P^*→B	10	9	24	20	20	5	10	100	3	7	22	10
S^*	5	5	2	6	3	7	4	3	100	9	1	8
S^*→A	26	27	7	10	12	16	32	7	9	100	7	16
S^*→B	15	15	22	19	18	7	14	22	1	7	100	10
S^*→P^*	14	16	10	10	12	27	18	10	8	16	10	100

1 100

Table 12. *Structure completeness comparison for the models generated from the original MR-NCS data sets. Each row corresponds to a pipeline variant, and shows the percentage (rounded to the nearest integer) of models that the pipeline variant built with at least 5% higher structure completeness than each of the other pipeline variants.*

Pipeline variant	A	A→P^*	A→B	B	B→P^*	P^*	$P^*\longrightarrow A$	$P^*\longrightarrow B$	S^*	$S^*\longrightarrow A$	$S^*\longrightarrow B$	$S^*\longrightarrow P^*$
A	0	6	8	16	7	16	7	10	43	20	14	14
A→P^*	24	0	12	19	8	13	16	12	54	29	18	14
A→B	29	18	0	25	13	22	19	10	63	31	18	27
B	26	15	14	0	8	20	19	9	55	35	12	21
B→P^*	30	20	18	25	0	27	20	14	63	36	21	22
P^*	26	12	13	23	5	0	15	9	52	28	19	11
$P^*\longrightarrow A$	15	14	13	21	10	17	0	11	53	24	19	16
$P^*\longrightarrow B$	30	23	17	27	15	28	22	0	60	35	20	33
S^*	16	7	9	8	4	5	5	5	0	20	9	5
$S^*\longrightarrow A$	8	6	7	14	6	14	1	9	38	0	10	13
$S^*\longrightarrow B$	27	17	12	20	11	18	18	11	61	31	0	22
$S^*\longrightarrow P^*$	22	11	14	22	5	7	15	10	51	24	18	0

0 63

Table 13. *Structure completeness comparison for the models generated from the original MR-NCS data sets. Each row corresponds to a pipeline variant, and shows the percentage (rounded to the nearest integer) of models that the pipeline variant built with between 1% and 4% higher structure completeness than each of the other pipeline variants.*

Pipeline variant	A	A→P^*	A→B	B	B→P^*	P^*	$P^*\longrightarrow A$	$P^*\longrightarrow B$	S^*	$S^*\longrightarrow A$	$S^*\longrightarrow B$	$S^*\longrightarrow P^*$
A	0	18	5	18	16	24	23	8	29	31	11	31
A→P^*	24	0	5	27	16	35	22	10	25	25	16	40
A→B	47	49	0	39	32	48	48	25	24	49	35	43
B	31	30	7	0	12	26	31	12	22	25	24	34
B→P^*	33	39	16	29	0	36	44	18	24	32	29	48
P^*	17	20	8	17	15	0	25	10	24	22	14	32
$P^*\longrightarrow A$	28	18	10	19	15	29	0	10	27	27	13	32
$P^*\longrightarrow B$	42	47	24	33	33	48	46	0	26	39	31	45
S^*	7	10	3	8	5	12	11	6	0	8	4	14
$S^*\longrightarrow A$	16	13	5	16	14	21	16	10	25	0	15	24
$S^*\longrightarrow B$	33	34	14	25	22	41	35	16	24	36	0	39
$S^*\longrightarrow P^*$	19	18	6	12	12	23	18	3	22	22	12	0

0 49

Table 14. *Comparison of R-work/R-free (rounded to two decimal places) for the models generated from the original MR-NCS data sets. Each row shows the percentage of models that a pipeline variant built with lower R-work or R-free than each other pipeline variant.*

Pipeline variant	A	A→P^*	A→B	B	B→P^*	P^*	P^*⟶A	P^*⟶B	S^*	S^*⟶A	S^*⟶B	S^*⟶P^*
A $_{R-work}$	0	21	88	91	27	35	18	90	100	31	93	37
A $_{R-free}$	-	-	-	-	-	-	-	-	-	-	-	-
A→P^* $_{R-work}$	64	0	93	97	37	61	56	89	100	61	93	59
A→P^* $_{R-free}$	-	0	89	91	36	54	78	80	-	88	88	55
A→B $_{R-work}$	7	3	0	52	2	2	2	32	99	5	45	3
A→B $_{R-free}$	-	6	0	54	5	3	16	35	-	30	48	5
B $_{R-work}$	6	3	18	0	1	3	1	17	99	5	25	3
B $_{R-free}$	-	7	22	0	2	4	11	24	-	24	30	4
B→P^* $_{R-work}$	59	31	96	99	0	54	52	95	100	57	97	61
B→P^* $_{R-free}$	-	37	89	95	0	50	77	93	-	84	93	53
P^* $_{R-work}$	45	18	93	93	20	0	33	94	100	40	95	26
P^* $_{R-free}$	-	23	93	93	29	0	70	95	-	76	96	30
P^*⟶A $_{R-work}$	40	27	95	95	30	42	0	94	100	35	95	45
P^*⟶A $_{R-free}$	-	15	74	85	12	12	0	73	-	50	80	16
P^*⟶B $_{R-work}$	6	6	28	51	3	3	4	0	100	8	39	5
P^*⟶B $_{R-free}$	-	13	31	54	4	2	18	0	-	27	44	6
S^* $_{R-work}$	0	0	1	1	0	0	0	0	0	0	0	0
S^* $_{R-free}$	-	-	-	-	-	-	-	-	-	-	-	-
S^*⟶A $_{R-work}$	33	21	90	93	24	38	16	91	100	0	92	41
S^*⟶A $_{R-free}$	-	8	65	68	11	9	16	67	-	0	69	13
S^*⟶B $_{R-work}$	5	3	19	39	1	2	3	21	100	4	0	2
S^*⟶B $_{R-free}$	-	8	20	43	4	2	12	27	-	28	0	5
S^*⟶P^* $_{R-work}$	42	14	94	94	17	20	31	90	100	37	95	0
S^*⟶P^* $_{R-free}$	-	22	90	93	25	32	72	90	-	78	94	0

0 100

Table 15. *Comparison of R-work/R-free (rounded to two decimal places) for the models generated from the original MR-NCS data sets. Each row shows the percentage of models that a pipeline variant built with equal R-work or R-free to each other pipeline variant.*

Pipeline variant	A	A→P^*	A→B	B	B→P^*	P^*	P^*→A	P^*→B	S^*	S^*→A	S^*→B	S^*→P^*
A $_{R-work}$	100	15	5	3	15	20	41	3	0	36	1	20
A $_{R-free}$	-	-	-	-	-	-	-	-	-	-	-	-
A→P^* $_{R-work}$	15	100	4	1	33	22	17	5	0	18	4	28
A→P^* $_{R-free}$	-	100	5	1	27	23	7	7	-	4	3	23
A→B $_{R-work}$	5	4	100	30	2	5	3	40	0	5	36	3
A→B $_{R-free}$	-	5	100	23	5	3	10	35	-	5	32	5
B $_{R-work}$	3	1	30	100	0	3	4	32	0	3	36	3
B $_{R-free}$	-	1	23	100	3	3	4	22	-	7	27	3
B→P^* $_{R-work}$	15	33	2	0	100	27	18	2	0	19	2	22
B→P^* $_{R-free}$	-	27	5	3	100	21	12	3	-	5	3	22
P^* $_{R-work}$	20	22	5	3	27	100	25	3	0	22	3	54
P^* $_{R-free}$	-	23	3	3	21	100	18	3	-	15	2	38
P^*→A $_{R-work}$	41	17	3	4	18	25	100	2	0	48	2	24
P^*→A $_{R-free}$	-	7	10	4	12	18	100	9	-	34	8	12
P^*→B $_{R-work}$	3	5	40	32	2	3	2	100	0	1	40	5
P^*→B $_{R-free}$	-	7	35	22	3	3	9	100	-	6	29	4
S^* $_{R-work}$	0	0	0	0	0	0	0	0	100	0	0	0
S^* $_{R-free}$	-	-	-	-	-	-	-	-	-	-	-	-
S^*→A $_{R-work}$	36	18	5	3	19	22	48	1	0	100	4	22
S^*→A $_{R-free}$	-	4	5	7	5	15	34	6	-	100	3	9
S^*→B $_{R-work}$	1	4	36	36	2	3	2	40	0	4	100	3
S^*→B $_{R-free}$	-	3	32	27	3	2	8	29	-	3	100	1
S^*→P^* $_{R-work}$	20	28	3	3	22	54	24	5	0	22	3	100
S^*→P^* $_{R-free}$	-	23	5	3	22	38	12	4	-	9	1	100

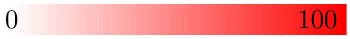

Table 16. *Comparison of R-work/R-free (rounded to two decimal places) for the models generated from the original MR-NCS data sets. Each row shows the percentage of models that a pipeline variant built with R-work or R-free at least 5% lower than each other pipeline variant.*

Pipeline variant	A	A→P^*	A→B	B	B→P^*	P^*	P^*→A	P^*→B	S^*	S^*→A	S^*→B	S^*→P^*
A $_{R-work}$	0	6	34	48	3	3	2	31	100	5	41	4
A $_{R-free}$	-	-	-	-	-	-	-	-	-	-	-	-
A→P^* $_{R-work}$	2	0	50	65	2	1	2	48	100	3	56	0
A→P^* $_{R-free}$	-	0	44	59	3	1	10	43	-	24	54	1
A→B $_{R-work}$	0	0	0	10	1	0	0	6	95	1	8	0
A→B $_{R-free}$	-	3	0	12	1	0	2	6	-	16	9	0
B $_{R-work}$	1	1	5	0	0	0	1	7	97	1	5	0
B $_{R-free}$	-	3	5	0	1	0	3	6	-	14	3	0
B→P^* $_{R-work}$	5	5	50	62	0	2	2	45	100	5	56	0
B→P^* $_{R-free}$	-	9	49	61	0	2	12	46	-	29	56	1
P^* $_{R-work}$	3	4	35	48	1	0	1	31	100	5	42	0
P^* $_{R-free}$	-	9	37	49	1	0	10	31	-	26	44	1
P^*→A $_{R-work}$	5	8	36	54	3	4	0	38	100	7	47	5
P^*→A $_{R-free}$	-	7	18	28	1	1	0	18	-	15	26	1
P^*→B $_{R-work}$	1	1	12	14	0	0	0	0	98	2	12	0
P^*→B $_{R-free}$	-	7	11	14	1	0	2	0	-	15	13	0
S^* $_{R-work}$	0	0	0	1	0	0	0	0	0	0	0	0
S^* $_{R-free}$	-	-	-	-	-	-	-	-	-	-	-	-
S^*→A $_{R-work}$	5	4	34	49	1	1	3	33	100	0	42	1
S^*→A $_{R-free}$	-	4	10	20	1	0	0	17	-	0	20	0
S^*→B $_{R-work}$	1	1	7	7	0	0	1	5	99	0	0	0
S^*→B $_{R-free}$	-	5	5	7	0	0	2	5	-	11	0	0
S^*→P^* $_{R-work}$	4	5	34	48	1	1	2	33	100	5	43	0
S^*→P^* $_{R-free}$	-	10	38	48	1	1	8	34	-	24	46	0

0 100

Table 17. *Comparison of R-work/R-free (rounded to two decimal places) for the models generated from the original MR-NCS data sets. Each row shows the percentage of models that a pipeline variant built with R-work or R-free between 1% and 4% lower than each other pipeline variant.*

Pipeline variant	A	A→P^*	A→B	B	B→P^*	P^*	P^*→A	P^*→B	S^*	S^*→A	S^*→B	S^*→P^*
A $_{R-work}$	0	15	54	44	23	31	16	59	0	25	52	33
A $_{R-free}$	-	-	-	-	-	-	-	-	-	-	-	-
A→P^* $_{R-work}$	62	0	44	31	35	59	54	41	0	58	36	59
A→P^* $_{R-free}$	-	0	46	32	33	53	69	37	-	64	34	54
A→B $_{R-work}$	7	3	0	41	1	2	2	26	3	4	37	3
A→B $_{R-free}$	-	3	0	43	5	3	14	29	-	14	39	5
B $_{R-work}$	5	2	14	0	1	3	1	10	1	4	20	3
B $_{R-free}$	-	5	18	0	1	4	8	18	-	10	27	4
B→P^* $_{R-work}$	54	26	46	37	0	52	50	50	0	52	40	61
B→P^* $_{R-free}$	-	29	40	34	0	48	65	46	-	54	37	52
P^* $_{R-work}$	41	14	57	46	19	0	31	63	0	35	53	26
P^* $_{R-free}$	-	14	56	44	29	0	60	63	-	50	52	29
P^*→A $_{R-work}$	35	19	59	41	27	38	0	56	0	29	48	40
P^*→A $_{R-free}$	-	7	56	57	11	12	0	56	-	35	54	15
P^*→B $_{R-work}$	5	5	16	37	3	3	4	0	2	6	27	5
P^*→B $_{R-free}$	-	6	20	41	3	2	16	0	-	12	31	6
S^* $_{R-work}$	0	0	1	0	0	0	0	0	0	0	0	0
S^* $_{R-free}$	-	-	-	-	-	-	-	-	-	-	-	-
S^*→A $_{R-work}$	29	17	56	44	23	37	13	59	0	0	50	39
S^*→A $_{R-free}$	-	4	55	48	10	9	16	50	-	0	48	13
S^*→B $_{R-work}$	5	2	12	31	1	2	2	16	1	4	0	2
S^*→B $_{R-free}$	-	3	15	36	4	2	10	22	-	17	0	5
S^*→P^* $_{R-work}$	38	9	60	46	16	19	29	58	0	33	52	0
S^*→P^* $_{R-free}$	-	12	52	45	24	31	64	56	-	54	48	0

0 69

Table 18. *Structure completeness comparison for the models generated from the original NO-NCS data sets. Each row corresponds to a pipeline variant, and shows the percentage (rounded to the nearest integer) of models that the pipeline variant built with higher structure completeness than each of the other pipeline variants.*

Pipeline variant	A	A→P^*	A→P	A→B	B	B→P^*	B→P	P^*	P^*→A	P^*→B	P	P→A	P→B	S	S→A	S→B	S^*	S^*→A	S^*→B	S^*→P^*	S^*→P	S→P^*	S→P
A	0	26	29	18	33	21	20	39	26	18	37	31	21	68	42	24	61	41	26	39	39	38	40
A→P^*	52	0	26	30	51	24	24	45	37	22	49	39	28	81	57	32	78	50	39	46	50	49	47
A→P	49	17	0	28	45	25	26	43	36	20	47	39	24	78	54	30	76	50	36	43	45	44	45
A→B	74	59	63	0	64	41	41	66	60	30	68	65	36	85	74	43	80	71	48	64	64	57	62
B	57	40	45	22	0	20	21	46	45	18	49	51	24	77	59	28	72	57	33	51	44	47	49
B→P^*	68	59	58	43	59	0	24	65	59	32	65	59	41	86	66	49	82	68	48	66	60	64	62
B→P	70	59	59	42	55	22	0	64	59	34	67	59	40	87	66	48	84	70	46	59	63	64	63
P^*	49	37	37	28	45	23	26	0	36	19	46	43	25	80	48	34	77	49	35	45	41	36	41
P^*→A	49	35	37	30	44	29	30	47	0	22	50	32	27	82	54	34	78	45	36	49	49	48	54
P^*→B	76	70	70	51	68	49	49	72	67	0	76	71	47	87	76	54	85	74	57	72	72	70	75
P	48	34	34	25	41	18	19	32	37	18	0	37	20	78	47	32	72	49	29	34	30	31	34
P→A	45	31	30	27	41	24	28	43	26	22	45	0	24	81	49	31	76	45	32	48	44	46	49
P→B	72	67	69	41	61	44	44	64	61	36	74	68	0	85	76	50	82	72	50	68	67	61	68
S	26	11	14	12	20	11	10	16	10	12	16	13	13	0	32	11	34	24	9	15	15	11	16
S→A	30	28	28	18	33	22	25	33	24	12	39	30	16	64	0	21	60	25	20	36	35	32	36
S→B	68	55	58	36	55	34	35	59	56	26	61	57	34	89	74	0	84	68	43	61	57	59	59
S^*	32	17	17	16	24	12	12	17	17	12	22	18	14	57	34	15	0	32	14	19	16	14	20
S^*→A	31	27	29	19	35	22	24	36	24	17	41	27	20	68	41	24	64	0	26	41	36	38	39
S^*→B	64	50	50	32	55	34	35	53	53	22	61	55	28	89	67	38	81	68	0	52	53	55	58
S^*→P^*	49	35	36	28	43	24	24	34	31	17	41	38	22	80	47	32	74	48	36	0	34	37	37
S^*→P	49	36	36	28	46	26	28	35	35	18	45	42	23	82	47	35	76	49	39	34	0	33	39
S→P^*	52	36	39	29	45	23	23	34	34	20	47	43	24	84	54	34	81	51	34	40	39	0	39
S→P	49	34	35	28	46	23	24	33	34	20	47	39	22	80	52	32	76	50	32	39	36	34	0

0 89

Table 19. *Structure completeness comparison for the models generated from the original NO-NCS data sets. Each row corresponds to a pipeline variant, and shows the percentage (rounded to the nearest integer) of models that the pipeline variant built with equal structure completeness to each of the other pipeline variants.*

Pipeline variant	A	A→P^*	A→P	A→B	B	B→P^*	B→P	P^*	P^*→A	P^*→B	P	P→A	P→B	S	S→A	S→B	S^*	S^*→A	S^*→B	S^*→P^*	S^*→P	S→P^*	S→P
A	100	22	22	7	9	11	11	12	25	6	15	24	7	5	28	7	7	28	9	12	12	10	11
A→P^*	22	100	57	11	9	16	17	18	28	8	18	30	5	7	15	13	5	23	11	19	14	15	20
A→P	22	57	100	9	11	17	14	20	26	11	19	31	7	7	18	11	7	21	14	21	18	18	20
A→B	7	11	9	100	14	17	17	6	9	19	7	8	22	3	7	21	4	10	20	8	8	14	9
B	9	9	11	14	100	21	24	9	11	14	11	9	16	3	8	17	4	7	11	7	10	9	5
B→P^*	11	16	17	17	21	100	54	12	12	19	18	17	15	2	11	18	5	9	18	11	14	14	15
B→P	11	17	14	17	24	54	100	10	11	18	14	13	16	3	9	17	4	7	19	16	9	14	13
P^*	12	18	20	6	9	12	10	100	17	9	22	14	11	4	19	7	6	15	11	22	24	30	26
P^*→A	25	28	26	9	11	12	11	17	100	11	13	42	12	7	22	10	5	32	11	20	16	18	12
P^*→B	6	8	11	19	14	19	18	9	11	100	7	7	17	1	11	20	3	9	21	11	9	10	5
P	15	18	19	7	11	18	14	22	13	7	100	18	7	6	15	7	7	10	10	25	26	22	18
P→A	24	30	31	8	9	17	13	14	42	7	18	100	7	6	21	12	5	28	13	14	14	11	13
P→B	7	5	7	22	16	15	16	11	12	17	7	7	100	2	8	16	4	8	22	10	10	14	10
S	5	7	7	3	3	2	3	4	7	1	6	6	2	100	3	1	8	7	1	5	3	5	4
S→A	28	15	18	7	8	11	9	19	22	11	15	21	8	3	100	5	5	34	13	17	18	14	11
S→B	7	13	11	21	17	18	17	7	10	20	7	12	16	1	5	100	1	8	20	6	7	7	9
S^*	7	5	7	4	4	5	4	6	5	3	7	5	4	8	5	1	100	5	5	7	8	5	4
S^*→A	28	23	21	10	7	9	7	15	32	9	10	28	8	7	34	8	5	100	7	11	15	11	11
S^*→B	9	11	14	20	11	18	19	11	11	21	10	13	22	1	13	20	5	7	100	12	9	11	9
S^*→P^*	12	19	21	8	7	11	16	22	20	11	25	14	10	5	17	6	7	11	12	100	32	23	24
S^*→P	12	14	18	8	10	14	9	24	16	9	26	14	10	3	18	7	8	15	9	32	100	28	25
S→P^*	10	15	18	14	9	14	14	30	18	10	22	11	14	5	14	7	5	11	11	23	28	100	27
S→P	11	20	20	9	5	15	13	26	12	5	18	13	10	4	11	9	4	11	9	24	25	27	100

1 100

Table 20. *Structure completeness comparison for the models generated from the original NO-NCS data sets. Each row corresponds to a pipeline variant, and shows the percentage (rounded to the nearest integer) of structures that the pipeline variant built with at least 5% higher structure completeness than each of the other pipeline variants.*

Pipeline variant	A	A$\rightarrow P^*$	A$\rightarrow P$	A$\rightarrow B$	B	B$\rightarrow P^*$	B$\rightarrow P$	P^*	$P^*\longrightarrow A$	$P^*\longrightarrow B$	P	P$\rightarrow A$	P$\rightarrow B$	S	S$\rightarrow A$	S$\rightarrow B$	S^*	$S^*\longrightarrow A$	$S^*\longrightarrow B$	$S^*\longrightarrow P^*$	$S^*\longrightarrow P$	S$\longrightarrow P^*$	S$\rightarrow P$
A	0	5	6	7	15	7	8	11	5	10	14	4	12	45	22	13	40	20	14	14	14	14	13
A$\rightarrow P^*$	30	0	9	16	28	9	10	13	10	11	21	16	14	61	31	20	57	30	21	14	14	15	14
A$\rightarrow P$	28	6	0	16	26	9	10	12	11	10	20	16	13	61	31	21	56	28	21	15	14	11	13
A$\rightarrow B$	30	13	17	0	30	11	14	24	16	9	28	20	11	64	34	18	57	29	18	24	24	21	25
B	28	11	11	12	0	9	7	16	13	8	16	18	8	56	33	14	48	29	14	22	17	16	18
B$\rightarrow P^*$	36	20	23	24	32	0	4	24	16	14	30	25	17	66	35	26	63	33	24	23	21	20	21
B$\rightarrow P$	35	20	24	24	30	5	0	21	17	14	29	24	18	64	37	26	62	34	24	22	22	22	23
P^*	28	14	14	18	26	9	9	0	13	9	14	17	13	61	30	24	55	27	22	10	6	9	9
$P^*\longrightarrow A$	26	14	18	20	28	13	12	19	0	10	22	12	15	59	30	24	57	28	23	19	20	14	16
$P^*\longrightarrow B$	33	23	25	19	35	18	21	30	22	0	30	29	16	70	40	24	63	34	24	30	30	28	32
P	28	11	15	16	23	8	7	7	14	9	0	15	13	57	32	21	51	26	20	11	9	7	7
P$\rightarrow A$	26	12	13	18	26	10	11	15	7	11	16	0	13	58	30	21	54	26	20	16	13	11	18
P$\rightarrow B$	35	20	20	19	34	17	18	26	20	11	32	25	0	65	39	24	63	36	24	28	27	25	27
S	17	7	6	10	11	5	5	7	3	5	7	7	6	0	24	7	9	17	3	7	7	6	5
S$\rightarrow A$	9	6	7	11	16	8	9	11	1	8	15	5	10	38	0	9	35	7	7	13	11	9	9
S$\rightarrow B$	36	18	18	15	26	12	11	22	18	12	28	24	11	70	38	0	59	38	18	28	24	24	24
S^*	21	7	6	13	17	5	5	5	6	5	10	7	7	32	27	11	0	20	9	5	6	5	5
$S^*\longrightarrow A$	12	7	9	12	20	8	9	12	2	9	16	3	11	47	16	11	40	0	9	14	13	11	10
$S^*\longrightarrow B$	31	16	17	14	28	11	12	19	12	9	23	20	11	65	35	12	60	30	0	22	20	22	20
$S^*\longrightarrow P^*$	28	12	14	19	28	8	9	9	12	10	18	17	14	61	30	21	53	24	22	0	5	5	7
$S^*\longrightarrow P$	30	14	14	19	32	7	9	10	12	11	17	16	14	63	33	24	52	27	20	7	0	3	7
S$\longrightarrow P^*$	30	14	16	21	30	9	10	13	15	11	16	18	14	60	29	24	53	26	23	11	10	0	11
S$\rightarrow P$	28	13	13	18	28	9	8	9	12	10	15	18	14	59	30	22	55	25	21	9	5	5	0

0 70

Table 21. *Structure completeness comparison for the models generated from the original NO-NCS data sets. Each row corresponds to a pipeline variant, and shows the percentage (rounded to the nearest integer) of models that the pipeline variant built with between 1% and 4% higher structure completeness than each of the other pipeline variants.*

Pipeline variant	A	A→P^*	A→P	A→B	B	B→P^*	B→P	P^*	P^*→A	P^*→B	P	P→A	P→B	S	S→A	S→B	S^*	S^*→A	S^*→B	S^*→P^*	S^*→P	S→P^*	S→P
A	0	22	23	11	18	14	11	27	21	8	23	27	9	24	20	11	21	21	13	25	26	24	27
A→P^*	22	0	18	14	23	16	14	32	27	11	28	23	14	20	26	12	21	20	18	32	36	34	33
A→P	20	11	0	12	18	16	16	31	26	9	26	24	11	18	23	9	20	22	15	28	32	32	32
A→B	45	46	46	0	34	29	28	42	44	21	40	45	25	21	41	24	23	42	30	40	41	36	37
B	30	29	33	10	0	11	14	30	32	10	32	33	16	21	26	14	24	28	19	29	27	30	31
B→P^*	32	39	35	19	27	0	20	41	43	19	35	34	24	20	31	22	20	35	24	43	39	44	41
B→P	34	40	36	18	26	18	0	43	42	20	38	35	22	23	28	22	22	36	22	37	41	42	40
P^*	21	23	24	10	19	14	17	0	24	9	32	26	12	20	18	10	22	22	13	34	34	27	32
P^*→A	22	21	19	11	16	16	18	28	0	12	28	20	12	23	24	10	20	16	13	30	29	34	38
P^*→B	43	47	45	32	33	30	28	41	45	0	46	42	31	18	36	30	22	40	33	42	43	43	43
P	20	22	20	9	18	9	12	25	24	9	0	22	7	21	15	11	21	23	9	23	21	24	28
P→A	18	19	17	9	15	14	18	28	19	10	29	0	11	23	19	10	22	19	13	32	31	34	30
P→B	36	47	49	22	26	27	26	39	41	26	41	43	0	20	36	26	20	36	26	41	40	36	41
S	9	5	8	2	9	7	5	9	7	7	9	5	7	0	8	3	25	7	6	8	7	5	11
S→A	21	22	22	7	17	14	16	22	24	4	24	25	6	26	0	11	25	18	13	23	24	23	27
S→B	32	36	40	22	30	22	24	37	39	14	32	33	22	19	36	0	26	30	25	34	34	35	34
S^*	11	10	11	3	7	7	7	11	11	7	11	11	6	26	7	4	0	11	5	14	9	9	14
S^*→A	19	20	20	7	15	14	15	24	22	8	26	24	9	22	26	13	24	0	17	27	23	26	28
S^*→B	33	34	33	19	28	24	23	34	41	14	38	35	16	24	32	26	21	37	0	30	32	33	39
S^*→P^*	22	23	22	9	14	16	16	25	19	7	22	21	7	20	18	11	22	24	14	0	29	32	30
S^*→P	19	23	22	9	14	18	20	25	23	7	28	26	9	19	14	11	24	22	18	27	0	30	32
S→P^*	22	22	22	8	15	14	13	21	20	9	31	25	10	24	25	10	28	25	11	29	29	0	28
S→P	20	21	22	10	18	14	16	24	22	9	32	21	9	21	22	9	21	25	11	30	31	29	0

0 49

Table 22. *Comparison of R-work/R-free (rounded to two decimal places) for the models generated from the original NO-NCS data sets. Each row shows the percentage of models that a pipeline variant built with lower R-work or R-free than each other pipeline variant.*

Pipeline variant	A	A→P^*	A→P	A→B	B	B→P^*	B→P	P^*	P^*→A	P^*→B	P	P→A	P→B	S	S→A	S→B	S^*	S^*→A	S^*→B	S^*→P^*	S^*→P	S→P^*	S→P
A $_{R-work}$	0	24	24	90	94	27	26	34	16	88	37	18	88	100	32	90	100	30	93	34	31	29	32
A $_{R-free}$	-	-	-	-	-	-	-	-	-	-	-	-	-	-	-	-	-	-	-	-	-	-	-
A→P^* $_{R-work}$	64	0	12	95	99	32	34	56	51	91	62	57	93	100	64	93	100	62	95	55	57	54	56
A→P^* $_{R-free}$	-	0	26	89	93	33	36	51	75	79	53	76	84	-	89	86	-	86	88	50	52	51	54
A→P $_{R-work}$	64	14	0	95	99	33	33	58	50	91	64	57	93	100	63	94	100	63	95	57	59	55	57
A→P $_{R-free}$	-	23	0	89	93	35	36	52	78	83	52	77	86	-	89	86	-	85	89	51	53	53	54
A→B $_{R-work}$	6	2	3	0	58	5	3	3	2	29	3	3	32	97	5	43	97	4	45	5	4	3	3
A→B $_{R-free}$	-	5	7	0	59	5	4	2	11	32	5	14	30	-	30	41	-	25	46	5	4	3	3
B $_{R-work}$	5	1	1	17	0	2	2	3	1	11	3	1	18	97	1	23	97	1	26	3	2	2	1
B $_{R-free}$	-	5	5	20	0	3	3	3	10	19	5	12	14	-	28	24	-	23	29	3	2	3	3
B→P^* $_{R-work}$	61	33	32	94	97	0	14	52	46	94	55	52	93	100	59	93	100	58	95	55	48	50	50
B→P^* $_{R-free}$	-	43	39	93	95	0	27	50	78	94	50	81	91	-	84	92	-	80	93	51	45	46	49
B→P $_{R-work}$	61	36	32	94	97	17	0	54	46	95	57	51	94	99	58	93	99	59	95	51	49	49	51
B→P $_{R-free}$	-	47	41	92	95	30	0	47	77	93	54	80	91	-	82	91	-	80	93	51	47	47	51
P^* $_{R-work}$	47	20	16	93	95	24	22	0	30	93	27	32	95	99	44	93	99	41	95	20	24	19	19
P^* $_{R-free}$	-	30	28	95	93	28	24	0	70	93	31	70	93	-	82	93	-	78	95	26	25	24	24
P^*→A $_{R-work}$	48	34	31	94	99	39	36	49	0	95	51	25	96	100	44	96	100	41	97	47	51	43	47
P^*→A $_{R-free}$	-	18	18	78	86	16	18	20	0	75	19	35	78	-	53	80	-	51	82	16	11	12	13
P^*→B $_{R-work}$	8	7	6	31	61	5	4	4	1	0	5	4	32	99	12	45	99	9	47	7	4	3	3
P^*→B $_{R-free}$	-	16	14	35	64	4	4	3	14	0	7	18	34	-	30	41	-	26	49	6	3	3	3
P $_{R-work}$	43	14	14	93	93	21	19	22	29	89	0	30	93	99	43	92	99	39	93	17	17	16	16
P $_{R-free}$	-	30	29	91	93	27	26	31	72	88	0	72	91	-	80	90	-	78	92	27	26	26	24
P→A $_{R-work}$	47	27	26	92	96	33	32	44	21	93	47	0	94	100	42	95	100	39	97	41	46	41	45
P→A $_{R-free}$	-	15	14	74	80	15	15	13	28	72	14	0	72	-	44	74	-	47	80	14	12	11	14
P→B $_{R-work}$	5	5	5	34	59	5	3	2	2	23	2	2	0	99	9	40	99	5	43	5	3	2	3
P→B $_{R-free}$	-	12	11	39	59	5	5	3	13	33	5	18	0	-	34	46	-	27	48	7	4	5	3
S $_{R-work}$	0	0	0	2	3	0	1	1	0	1	1	0	1	0	0	0	19	0	0	0	0	0	0
S $_{R-free}$	-	-	-	-	-	-	-	-	-	-	-	-	-	-	-	-	-	-	-	-	-	-	-
S→A $_{R-work}$	32	21	19	94	98	24	25	36	16	85	41	20	85	100	0	93	100	20	95	34	34	32	31
S→A $_{R-free}$	-	8	7	61	64	11	14	9	22	64	13	22	61	-	0	59	-	26	61	9	8	9	9
S→B $_{R-work}$	6	5	4	24	47	5	5	1	1	19	5	2	26	100	2	0	100	2	34	3	2	3	3
S→B $_{R-free}$	-	11	10	28	50	6	7	5	11	25	7	18	24	-	34	0	-	28	38	5	5	3	4
S^* $_{R-work}$	0	0	0	2	3	0	1	1	0	1	1	0	1	42	0	0	0	0	0	0	0	0	0
S^* $_{R-free}$	-	-	-	-	-	-	-	-	-	-	-	-	-	-	-	-	-	-	-	-	-	-	-
S^*→A $_{R-work}$	35	22	21	92	95	26	26	36	15	89	39	17	89	100	30	93	100	0	95	35	37	34	36
S^*→A $_{R-free}$	-	11	10	69	71	12	12	11	19	69	12	23	66	-	36	69	-	0	68	11	9	9	10
S^*→B $_{R-work}$	4	2	2	23	45	4	3	3	2	18	4	3	20	100	1	25	100	2	0	4	2	3	2
S^*→B $_{R-free}$	-	7	7	24	52	5	5	3	10	24	5	13	24	-	30	31	-	24	0	5	4	3	3
S^*→P^* $_{R-work}$	48	18	14	93	94	24	18	24	32	91	30	34	92	100	42	94	100	42	93	0	19	18	18
S^*→P^* $_{R-free}$	-	32	29	90	93	26	26	29	74	90	36	72	89	-	85	91	-	80	93	0	27	22	27
S^*→P $_{R-work}$	47	20	18	93	95	20	18	24	32	92	28	34	93	100	43	92	100	41	93	16	0	17	16
S^*→P $_{R-free}$	-	32	27	93	94	26	26	31	69	91	34	74	93	-	81	92	-	80	93	28	0	26	25
S→P^* $_{R-work}$	48	22	20	93	96	24	20	24	31	93	34	33	93	100	45	94	100	42	95	22	27	0	20
S→P^* $_{R-free}$	-	30	28	93	95	27	26	34	76	92	36	76	95	-	83	92	-	80	94	28	30	0	33
S→P $_{R-work}$	46	20	18	93	96	26	21	25	30	93	31	35	94	100	41	93	100	42	94	20	22	16	0
S→P $_{R-free}$	-	31	29	93	95	32	27	32	70	92	38	74	93	-	84	94	-	78	95	27	24	27	0

0 100

Table 23. *Comparison of R-work/R-free (rounded to two decimal places) for the models generated from the original NO-NCS data sets. Each row shows the percentage of models that a pipeline variant built with equal R-work or R-free to each other pipeline variant.*

Pipeline variant	A	A→P^*	A→P	A→B	B	B→P^*	B→P	P^*	P^*→A	P^*→B	P	P→A	P→B	S	S→A	S→B	S^*	S^*→A	S^*→B	S^*→P^*	S^*→P	S→P^*	S→P
A $_{R-work}$	100	12	12	4	1	11	13	20	36	4	20	35	7	0	36	4	0	35	3	18	22	23	22
A $_{R-free}$	-	-	-	-	-	-	-	-	-	-	-	-	-	-	-	-	-	-	-	-	-	-	-
A→P^* $_{R-work}$	12	100	74	3	1	34	30	24	15	3	24	16	2	0	16	2	0	16	3	28	24	24	24
A→P^* $_{R-free}$	-	100	51	6	2	24	18	20	7	5	18	9	4	-	3	3	-	3	5	18	16	18	15
A→P $_{R-work}$	12	74	100	1	0	34	35	26	19	3	22	18	2	0	18	2	0	16	3	29	23	26	25
A→P $_{R-free}$	-	51	100	4	3	26	23	20	4	3	19	9	3	-	4	3	-	5	4	20	20	18	17
A→B $_{R-work}$	4	3	1	100	25	1	3	4	4	40	3	5	34	1	1	32	1	4	32	1	3	3	3
A→B $_{R-free}$	-	6	4	100	21	3	4	3	10	33	4	12	31	-	9	30	-	6	30	5	3	3	3
B $_{R-work}$	1	1	0	25	100	1	1	3	0	28	4	3	24	0	1	30	0	3	28	3	3	2	3
B $_{R-free}$	-	2	3	21	100	3	3	4	3	18	3	8	26	-	8	26	-	6	19	3	4	2	2
B→P^* $_{R-work}$	11	34	34	1	1	100	69	24	16	1	24	15	3	0	17	2	0	16	1	22	32	26	24
B→P^* $_{R-free}$	-	24	26	3	3	100	43	22	5	2	23	4	3	-	5	2	-	7	3	24	28	27	18
B→P $_{R-work}$	13	30	35	3	1	69	100	24	18	1	24	18	3	0	17	3	0	14	1	30	33	31	28
B→P $_{R-free}$	-	18	23	4	3	43	100	29	5	3	20	5	5	-	4	2	-	7	2	22	27	27	22
P^* $_{R-work}$	20	24	26	4	3	24	24	100	21	3	51	24	3	0	20	5	0	24	3	55	53	57	56
P^* $_{R-free}$	-	20	20	3	4	22	29	100	10	5	38	18	3	-	9	3	-	10	3	45	44	43	44
P^*→A $_{R-work}$	36	15	19	4	0	16	18	21	100	4	20	54	2	0	40	3	0	45	1	20	17	26	23
P^*→A $_{R-free}$	-	7	4	10	3	5	5	10	100	11	9	37	9	-	26	8	-	30	8	10	20	11	17
P^*→B $_{R-work}$	4	3	3	40	28	1	1	3	4	100	6	3	45	0	3	36	0	2	34	2	4	4	3
P^*→B $_{R-free}$	-	5	3	33	18	2	3	5	11	100	5	10	32	-	5	34	-	5	27	4	6	5	5
P $_{R-work}$	20	24	22	3	4	24	24	51	20	6	100	23	5	0	16	3	0	22	3	53	55	50	53
P $_{R-free}$	-	18	19	4	3	23	20	38	9	5	100	14	4	-	7	3	-	9	3	36	40	39	38
P→A $_{R-work}$	35	16	18	5	3	15	18	24	54	3	23	100	4	0	38	3	0	44	1	24	20	26	20
P→A $_{R-free}$	-	9	9	12	8	4	5	18	37	10	14	100	9	-	34	9	-	30	7	15	14	13	12
P→B $_{R-work}$	7	2	2	34	24	3	3	3	2	45	5	4	100	0	5	34	0	6	38	3	5	5	3
P→B $_{R-free}$	-	4	3	31	26	3	5	3	9	32	4	9	100	-	5	30	-	7	28	3	3	0	3
S $_{R-work}$	0	0	0	1	0	0	0	0	0	0	0	0	0	100	0	0	39	0	0	0	0	0	0
S $_{R-free}$	-	-	-	-	-	-	-	-	-	-	-	-	-	-	-	-	-	-	-	-	-	-	-
S→A $_{R-work}$	36	16	18	1	1	17	17	20	40	3	16	38	5	0	100	5	0	49	3	24	24	24	28
S→A $_{R-free}$	-	3	4	9	8	5	4	9	26	5	7	34	5	-	100	7	-	39	9	5	11	8	7
S→B $_{R-work}$	4	2	2	32	30	2	3	5	3	36	3	3	34	0	5	100	0	5	41	3	6	3	4
S→B $_{R-free}$	-	3	3	30	26	2	2	3	8	34	3	9	30	-	7	100	-	3	31	3	3	5	2
S^* $_{R-work}$	0	0	0	1	0	0	0	0	0	0	0	0	0	39	0	0	100	0	0	0	0	0	0
S^* $_{R-free}$	-	-	-	-	-	-	-	-	-	-	-	-	-	-	-	-	-	-	-	-	-	-	-
S^*→A $_{R-work}$	35	16	16	4	3	16	14	24	45	2	22	44	6	0	49	5	0	100	3	23	22	24	22
S^*→A $_{R-free}$	-	3	5	6	6	7	7	10	30	5	9	30	7	-	39	3	-	100	7	9	11	10	12
S^*→B $_{R-work}$	3	3	3	32	28	1	1	3	1	34	3	1	38	0	3	41	0	3	100	3	5	3	4
S^*→B $_{R-free}$	-	5	4	30	19	3	2	3	8	27	3	7	28	-	9	31	-	7	100	1	3	3	3
S^*→P^* $_{R-work}$	18	28	29	1	3	22	30	55	20	2	53	24	3	0	24	3	0	23	3	100	65	60	62
S^*→P^* $_{R-free}$	-	18	20	5	3	24	22	45	10	4	36	15	3	-	5	3	-	9	1	100	45	50	46
S^*→P $_{R-work}$	22	24	23	3	3	32	33	53	17	4	55	20	5	0	24	6	0	22	5	65	100	56	62
S^*→P $_{R-free}$	-	16	20	3	4	28	27	44	20	6	40	14	3	-	11	3	-	11	3	45	100	44	51
S→P^* $_{R-work}$	23	24	26	3	2	26	31	57	26	4	50	26	5	0	24	3	0	24	3	60	56	100	64
S→P^* $_{R-free}$	-	18	18	3	2	27	27	43	11	5	39	13	0	-	8	5	-	10	3	50	44	100	40
S→P $_{R-work}$	22	24	25	3	3	24	28	56	23	3	53	20	3	0	28	4	0	22	4	62	62	64	100
S→P $_{R-free}$	-	15	17	3	2	18	22	44	17	5	38	12	3	-	7	2	-	12	3	46	51	40	100

0 100

Table 24. *Comparison of R-work/R-free (rounded to two decimal places) for the models generated from the original NO-NCS data sets. Each row shows the percentage of models that a pipeline variant built with R-work or R-free between 1% and 4% lower than each other pipeline variant.*

Pipeline variant	A	A→P^*	A→P	A→B	B	B→P^*	B→P	P^*	P^*→A	P^*→B	P	P→A	P→B	S	S→A	S→B	S^*	S^*→A	S^*→B	S^*→P^*	S^*→P	S→P^*	S→P
A $_{R-work}$	0	18	18	51	42	24	22	29	16	59	30	15	53	0	22	45	0	23	51	30	28	27	30
A $_{R-free}$	–	–	–	–	–	–	–	–	–	–	–	–	–	–	–	–	–	–	–	–	–	–	–
A→P^* $_{R-work}$	58	0	12	38	32	29	30	53	48	44	59	53	39	0	54	36	0	57	35	53	55	53	54
A→P^* $_{R-free}$	–	0	26	37	30	29	31	49	65	34	51	66	39	–	61	33	–	64	34	48	50	50	51
A→P $_{R-work}$	57	14	0	36	32	30	29	55	47	44	61	53	39	0	53	37	0	56	38	55	57	54	55
A→P $_{R-free}$	–	22	0	36	32	31	32	51	70	41	49	66	42	–	59	32	–	64	36	49	51	52	52
A→B $_{R-work}$	6	2	3	0	48	5	3	3	2	24	3	3	28	3	5	33	3	3	39	5	3	3	3
A→B $_{R-free}$	–	3	4	0	47	5	4	2	9	29	4	13	26	–	13	32	–	11	39	5	3	3	3
B $_{R-work}$	5	1	1	11	0	2	2	3	1	7	2	1	14	2	1	20	3	1	23	3	1	2	1
B $_{R-free}$	–	3	3	15	0	3	3	3	9	14	4	11	9	–	9	21	–	9	26	3	1	3	2
B→P^* $_{R-work}$	56	28	29	41	35	0	14	49	44	52	50	48	41	0	49	37	0	53	39	53	46	49	49
B→P^* $_{R-free}$	–	31	27	41	30	0	26	47	66	51	47	66	42	–	57	36	–	57	36	48	44	45	48
B→P $_{R-work}$	55	32	28	42	32	17	0	51	43	52	54	47	43	0	49	36	0	54	38	49	47	48	50
B→P $_{R-free}$	–	35	28	39	30	30	0	43	63	52	50	61	42	–	52	36	–	53	37	49	45	47	49
P^* $_{R-work}$	42	16	13	54	41	22	19	0	28	59	25	29	53	0	34	49	0	34	49	19	23	18	18
P^* $_{R-free}$	–	19	18	53	36	25	20	0	61	58	29	58	51	–	56	48	–	55	49	25	24	23	23
P^*→A $_{R-work}$	42	24	23	47	39	33	31	43	0	53	43	20	49	0	30	48	0	31	46	40	45	39	42
P^*→A $_{R-free}$	–	10	9	53	49	15	16	18	0	57	16	32	57	–	32	53	–	35	55	15	11	11	12
P^*→B $_{R-work}$	7	6	5	17	43	5	4	4	1	0	4	3	28	1	9	28	1	7	34	6	3	3	3
P^*→B $_{R-free}$	–	9	7	21	46	4	4	3	14	0	5	16	29	–	11	26	–	10	35	6	3	3	2
P $_{R-work}$	39	11	13	55	39	18	16	21	28	55	0	29	53	0	36	49	1	35	48	14	16	16	15
P $_{R-free}$	–	22	20	50	35	23	22	30	64	55	0	63	51	–	56	48	–	57	48	24	25	25	23
P→A $_{R-work}$	43	18	18	47	38	28	26	39	19	56	41	0	52	0	29	48	0	30	49	34	41	37	41
P→A $_{R-free}$	–	7	5	49	45	12	12	12	27	51	12	0	55	–	24	50	–	32	54	11	11	10	14
P→B $_{R-work}$	5	5	5	22	39	5	3	2	2	19	1	2	0	2	8	26	2	4	30	5	3	2	3
P→B $_{R-free}$	–	7	5	27	41	5	5	3	9	30	5	16	0	–	13	32	–	9	37	7	4	5	3
S $_{R-work}$	0	0	0	1	1	0	1	0	0	0	1	0	0	0	0	0	19	0	0	0	0	0	0
S $_{R-free}$	–	–	–	–	–	–	–	–	–	–	–	–	–	–	–	–	–	–	–	–	–	–	–
S→A $_{R-work}$	27	16	15	53	47	22	22	36	14	55	39	17	50	0	0	47	0	20	53	33	32	30	30
S→A $_{R-free}$	–	4	3	47	42	9	11	9	22	49	11	20	50	–	0	43	–	26	47	9	7	8	9
S→B $_{R-work}$	6	4	3	15	38	5	4	1	1	14	3	2	22	2	2	0	3	1	32	3	2	3	3
S→B $_{R-free}$	–	7	5	19	41	6	6	4	9	20	5	15	18	–	15	0	–	14	35	5	5	3	3
S^* $_{R-work}$	0	0	0	1	1	0	1	1	0	0	1	0	0	41	0	0	0	0	0	0	0	0	0
S^* $_{R-free}$	–	–	–	–	–	–	–	–	–	–	–	–	–	–	–	–	–	–	–	–	–	–	–
S^*→A $_{R-work}$	31	18	18	50	43	23	23	34	14	59	35	13	52	0	28	47	0	0	48	32	36	32	35
S^*→A $_{R-free}$	–	7	5	52	44	10	9	11	19	51	11	21	49	–	30	51	–	0	51	11	8	9	9
S^*→B $_{R-work}$	4	1	1	15	34	4	3	2	2	15	3	3	14	1	1	18	1	2	0	4	1	3	1
S^*→B $_{R-free}$	–	3	3	16	41	5	5	2	9	22	3	10	18	–	13	25	–	12	0	5	3	3	2
S^*→P^* $_{R-work}$	43	13	11	51	39	21	15	22	30	56	27	31	47	0	33	48	0	35	44	0	18	18	18
S^*→P^* $_{R-free}$	–	22	18	44	38	22	22	27	64	55	34	61	45	–	61	45	–	57	44	0	26	22	26
S^*→P $_{R-work}$	43	15	14	53	40	18	15	22	31	57	25	32	47	0	33	46	0	35	45	16	0	17	16
S^*→P $_{R-free}$	–	22	16	45	35	22	22	29	58	55	32	63	47	–	55	43	–	55	41	28	0	26	25
S→P^* $_{R-work}$	44	16	16	53	40	21	17	22	30	59	31	30	50	0	35	48	0	36	47	20	26	0	19
S→P^* $_{R-free}$	–	18	16	51	37	24	20	32	66	55	33	62	51	–	58	45	–	57	46	27	29	0	32
S→P $_{R-work}$	42	14	14	53	43	23	18	23	29	58	28	32	51	0	32	48	0	36	47	19	22	16	0
S→P $_{R-free}$	–	20	17	47	37	30	23	30	59	57	35	64	49	–	58	45	–	55	44	26	24	27	0

0 70

Table 25. *Comparison of R-work/R-free (rounded to two decimal places) for the models generated from the original NO-NCS data sets. Each row shows the percentage of models that a pipeline variant built with R-work or R-free at least 5% lower than each other pipeline variant.*

Pipeline variant	A	A→P^*	A→P	A→B	B	B→P^*	B→P	P^*	P^*→A	P^*→B	P	P→A	P→B	S	S→A	S→B	S^*	S^*→A	S^*→B	S^*→P^*	S^*→P	S→P^*	S→P
A $_{R-work}$	0	7	6	39	52	3	5	5	1	28	7	3	34	100	10	45	100	7	43	4	3	2	2
A $_{R-free}$	-	-	-	-	-	-	-	-	-	-	-	-	-	-	-	-	-	-	-	-	-	-	-
A→P^* $_{R-work}$	5	0	0	57	67	3	4	3	3	47	3	3	53	100	9	57	100	5	59	2	2	1	2
A→P^* $_{R-free}$	-	0	0	51	63	4	5	1	10	45	2	10	45	-	27	53	-	23	53	2	2	1	3
A→P $_{R-work}$	6	1	0	59	67	3	4	3	3	47	2	3	54	100	9	57	100	7	57	2	2	1	1
A→P $_{R-free}$	-	1	0	53	60	4	5	1	8	43	3	11	45	-	29	54	-	22	53	2	2	1	2
A→B $_{R-work}$	0	0	0	0	10	0	0	0	0	5	1	0	4	95	0	10	94	1	7	0	1	0	1
A→B $_{R-free}$	-	3	3	0	11	0	0	0	2	3	1	1	4	-	17	9	-	14	7	0	1	0	1
B $_{R-work}$	0	0	0	5	0	0	0	0	0	4	1	0	3	95	0	3	94	1	3	0	1	0	1
B $_{R-free}$	-	2	1	5	0	0	0	0	1	5	1	1	5	-	18	3	-	14	3	0	1	0	1
B→P^* $_{R-work}$	5	5	3	53	62	0	1	3	2	42	5	4	52	100	9	55	100	5	56	2	2	1	1
B→P^* $_{R-free}$	-	11	11	52	64	0	1	3	13	43	3	16	49	-	28	56	-	24	57	3	1	1	1
B→P $_{R-work}$	6	4	3	52	66	0	0	3	3	43	3	4	51	99	9	56	99	5	57	2	3	1	1
B→P $_{R-free}$	-	11	12	53	65	0	0	4	14	41	4	18	49	-	30	55	-	27	55	3	2	1	3
P^* $_{R-work}$	5	4	3	39	54	3	3	0	1	34	2	3	42	99	9	44	99	6	45	1	1	1	1
P^* $_{R-free}$	-	11	10	41	57	3	4	0	9	34	2	11	43	-	26	45	-	23	46	1	1	1	1
P^*→A $_{R-work}$	6	10	8	47	59	5	5	6	0	42	8	5	47	100	14	48	100	9	51	7	5	5	5
P^*→A $_{R-free}$	-	8	9	26	37	1	3	1	0	18	3	3	20	-	21	27	-	16	26	1	1	1	1
P^*→B $_{R-work}$	1	1	1	14	18	0	0	0	0	0	1	1	4	99	3	16	99	2	14	1	1	0	1
P^*→B $_{R-free}$	-	6	6	14	18	0	0	0	1	0	1	3	5	-	19	16	-	16	14	0	1	0	1
P $_{R-work}$	4	3	1	38	55	3	3	1	1	34	0	1	40	99	7	43	98	4	45	3	1	1	1
P $_{R-free}$	-	8	9	41	57	4	4	1	8	33	0	9	40	-	24	42	-	21	44	3	1	1	1
P→A $_{R-work}$	4	9	7	45	58	5	6	5	2	36	6	0	42	100	13	47	100	9	47	7	5	3	3
P→A $_{R-free}$	-	8	9	25	34	3	3	1	1	21	2	0	17	-	20	24	-	16	26	2	1	1	1
P→B $_{R-work}$	0	0	0	12	20	0	0	0	0	4	1	0	0	97	1	14	97	1	12	0	0	0	0
P→B $_{R-free}$	-	5	5	11	18	0	0	0	4	3	1	3	0	-	21	14	-	18	11	0	0	0	0
S $_{R-work}$	0	0	0	1	1	0	0	1	0	1	1	0	1	0	0	0	0	0	0	0	0	0	0
S $_{R-free}$	-	-	-	-	-	-	-	-	-	-	-	-	-	-	-	-	-	-	-	-	-	-	-
S→A $_{R-work}$	5	5	4	41	51	3	3	1	3	30	2	3	35	100	0	46	100	1	43	1	1	1	1
S→A $_{R-free}$	-	4	5	14	22	1	2	1	0	16	1	2	11	-	0	17	-	0	14	1	1	1	1
S→B $_{R-work}$	0	1	1	9	9	0	1	1	0	5	1	0	5	98	0	0	97	1	3	0	0	0	0
S→B $_{R-free}$	-	4	5	9	9	0	1	1	3	5	1	3	7	-	19	0	-	15	3	0	0	0	1
S^* $_{R-work}$	0	0	0	1	1	0	0	0	0	1	1	0	1	1	0	0	0	0	0	0	0	0	0
S^* $_{R-free}$	-	-	-	-	-	-	-	-	-	-	-	-	-	-	-	-	-	-	-	-	-	-	-
S^*→A $_{R-work}$	4	4	3	42	53	3	3	2	1	30	3	4	36	100	3	45	100	0	47	3	1	1	1
S^*→A $_{R-free}$	-	4	5	17	27	2	3	1	0	18	1	2	17	-	6	18	-	0	18	1	1	1	1
S^*→B $_{R-work}$	0	1	1	8	11	0	1	1	0	3	1	0	5	99	0	7	99	0	0	0	1	0	1
S^*→B $_{R-free}$	-	5	5	8	11	0	1	1	1	3	1	3	6	-	17	6	-	12	0	0	1	0	1
S^*→P^* $_{R-work}$	5	5	3	42	55	3	3	2	3	35	3	3	45	100	9	46	100	7	49	0	1	0	1
S^*→P^* $_{R-free}$	-	11	11	46	55	3	5	2	9	35	3	11	45	-	24	47	-	22	49	0	1	0	1
S^*→P $_{R-work}$	5	5	3	41	55	2	3	2	1	34	3	3	46	100	9	46	100	6	49	1	0	0	0
S^*→P $_{R-free}$	-	11	11	47	59	4	5	2	11	36	3	11	46	-	26	49	-	24	51	1	0	0	0
S→P^* $_{R-work}$	4	5	4	41	56	3	3	2	1	33	3	3	43	100	9	46	100	6	48	2	1	0	1
S→P^* $_{R-free}$	-	12	12	43	57	3	5	1	10	36	3	14	44	-	25	47	-	23	48	1	1	0	1
S→P $_{R-work}$	4	6	5	41	53	3	3	2	1	35	3	3	43	100	9	45	100	6	47	1	0	0	0
S→P $_{R-free}$	-	11	12	46	58	3	4	2	11	34	3	10	44	-	26	49	-	23	51	1	1	0	0

0 100

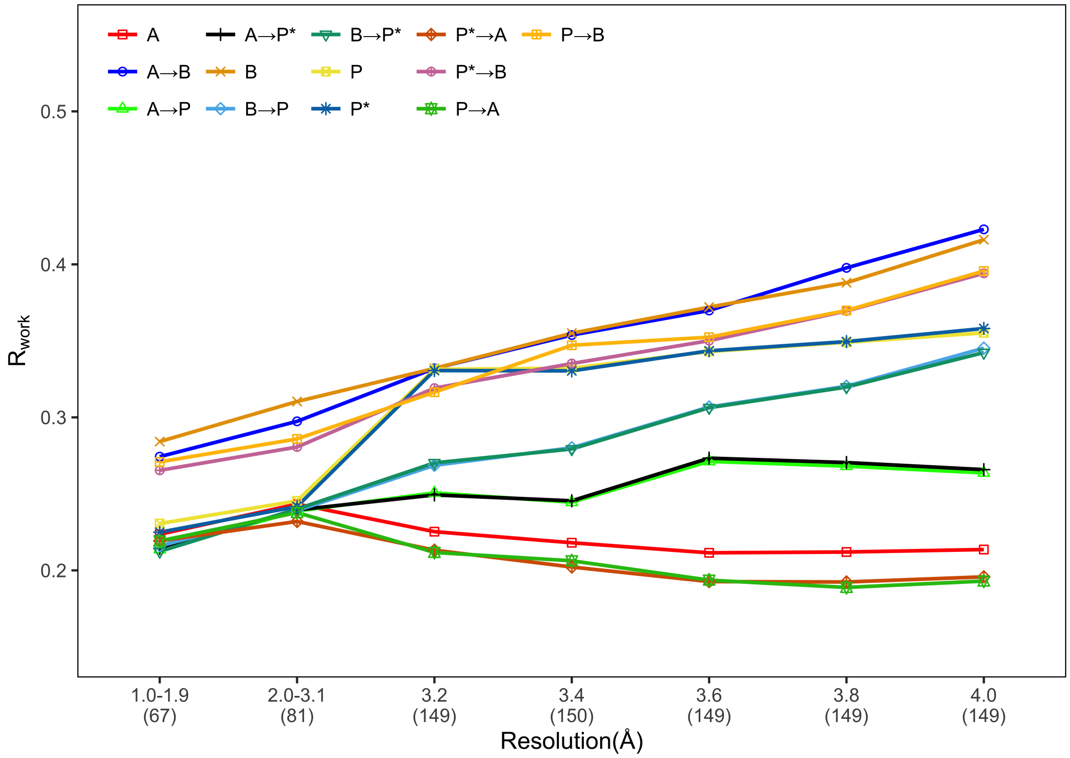

Fig. 1. Mean protein model R-work for the NO-NCS data sets partitioned into classes based on their resolution. The number of data sets in each class is indicated in brackets under the graph.

Appendix B
The Results of the Synthetic data sets without the Buccaneer Development Data sets

Table 1. *Complete and intermediate models produced by the 13 pipeline variants for the synthetic-resolution data sets, where '(T)' and '(C)' denote intermediate models produced by pipeline executions that timed out and crashed, respectively.*

Pipeline variant	HA-NCS			MR-NCS			NO-NCS		
	Complete	Intermediate	Failed	Complete	Intermediate	Failed	Complete	Intermediate	Failed
A	1008	1(T) 0(C)	0	1007	2(T) 0(C)	0	1008	1(T) 0(C)	0
A→P^*	1006	2(T) 0(C)	1	1006	2(T) 0(C)	1	1007	2(T) 0(C)	0
A→B	1009	0(T) 0(C)	0	1009	0(T) 0(C)	0	1009	0(T) 0(C)	0
B	1009	0(T) 0(C)	0	1009	0(T) 0(C)	0	1009	0(T) 0(C)	0
B→P^*	1003	1(T) 0(C)	5	1004	0(T) 0(C)	5	1005	0(T) 0(C)	4
P^*	1002	7(T) 0(C)	0	1004	5(T) 0(C)	0	1001	8(T) 0(C)	0
P^*→A	1008	1(T) 0(C)	0	1009	0(T) 0(C)	0	1008	1(T) 0(C)	0
P^*→B	1009	0(T) 0(C)	0	1009	0(T) 0(C)	0	1009	0(T) 0(C)	0
A→P	-	-	-	-	-	-	1009	0(T) 0(C)	0
B→P	-	-	-	-	-	-	1003	2(T) 0(C)	4
P	-	-	-	-	-	-	1001	7(T) 0(C)	1
P→A	-	-	-	-	-	-	1002	6(T) 0(C)	1
P→B	-	-	-	-	-	-	1008	0(T) 0(C)	1

Models used in the comparison: 744 HA-NCS, 745 MR-NCS and 746 NO-NCS.

Table 2. *Structure completeness comparison for the models generated from the synthetic HA-NCS data sets. Each row corresponds to a pipeline variant, and shows the percentage (rounded to the nearest integer) of models that the pipeline variant built with higher structure completeness than each of the other pipeline variants.*

Pipeline variant	A	A→P^*	A→B	B	B→P^*	P^*	P^*→A	P^*→B
A	0	1	1	1	0	2	28	1
A→P^*	95	0	15	12	3	23	96	6
A→B	93	80	0	41	29	72	93	25
B	93	83	50	0	25	75	94	30
B→P^*	98	95	66	69	0	86	99	45
P^*	97	71	26	23	10	0	97	13
P^*→A	15	1	1	1	0	2	0	0
P^*→B	96	92	70	63	50	84	97	0

0 99

Table 3. *Structure completeness comparison for the models generated from the synthetic HA-NCS data sets. Each row corresponds to a pipeline variant, and shows the percentage (rounded to the nearest integer) of models that the pipeline variant built with equal structure completeness to each of the other pipeline variants.*

Pipeline variant	A	A→P^*	A→B	B	B→P^*	P^*	P^*→A	P^*→B
A	100	4	6	5	1	1	57	3
A→P^*	4	100	5	5	2	6	3	2
A→B	6	5	100	9	5	3	6	5
B	5	5	9	100	6	2	5	7
B→P^*	1	2	5	6	100	4	1	5
P^*	1	6	3	2	4	100	1	3
P^*→A	57	3	6	5	1	1	100	3
P^*→B	3	2	5	7	5	3	3	100

1 100

Table 4. *Structure completeness comparison for the models generated from the synthetic HA-NCS data sets. Each row corresponds to a pipeline variant, and shows the percentage (rounded to the nearest integer) of models that the pipeline variant built with at least 5% higher structure completeness than each of the other pipeline variants.*

Pipeline variant	A	A→P^*	A→B	B	B→P^*	P^*	P^*→A	P^*→B
A	0	0	0	1	0	2	7	0
A→P^*	77	0	6	5	1	14	78	2
A→B	82	73	0	28	22	68	83	17
B	84	77	36	0	14	69	85	20
B→P^*	94	88	52	48	0	79	95	32
P^*	92	47	18	16	4	0	93	7
P^*→A	2	0	0	0	0	1	0	0
P^*→B	92	87	55	50	39	79	92	0

0 95

Table 5. *Structure completeness comparison for the models generated from the synthetic HA-NCS data sets. Each row corresponds to a pipeline variant, and shows the percentage (rounded to the nearest integer) of models that the pipeline variant built with between 1% and 4% higher structure completeness than each of the other pipeline variants.*

Pipeline variant	A	A→P^*	A→B	B	B→P^*	P^*	P^*→A	P^*→B
A	0	1	1	1	0	0	21	0
A→P^*	19	0	9	8	2	9	18	3
A→B	11	7	0	13	8	3	10	9
B	9	6	14	0	11	5	9	10
B→P^*	4	6	13	21	0	8	4	13
P^*	5	24	8	7	6	0	4	6
P^*→A	13	0	1	1	0	0	0	0
P^*→B	5	5	15	14	11	5	5	0

0 24

Table 6. *Comparison of R-work/R-free (rounded to two decimal places) for the models generated from the synthetic HA-NCS data sets. Each row shows the percentage of models that a pipeline variant built with lower R-work or R-free than each other pipeline variant.*

Pipeline variant	A	A→P^*	A→B	B	B→P^*	P^*	P^*→A	P^*→B
A $_{R-work}$	0	83	94	93	86	97	28	91
A $_{R-free}$	-	-	-	-	-	-	-	-
A→P^* $_{R-work}$	13	0	89	89	71	99	3	85
A→P^* $_{R-free}$	-	0	59	56	29	55	90	49
A→B $_{R-work}$	5	8	0	36	7	34	2	22
A→B $_{R-free}$	-	36	0	42	10	36	79	29
B $_{R-work}$	6	9	51	0	4	37	1	29
B $_{R-free}$	-	38	48	0	5	38	80	34
B→P^* $_{R-work}$	11	23	90	92	0	82	4	80
B→P^* $_{R-free}$	-	67	87	91	0	76	97	77
P^* $_{R-work}$	2	0	62	59	11	0	0	49
P^* $_{R-free}$	-	40	59	56	18	0	94	47
P^*→A $_{R-work}$	60	95	97	98	93	100	0	97
P^*→A $_{R-free}$	-	7	16	16	2	5	0	10
P^*→B $_{R-work}$	8	12	65	60	14	45	2	0
P^*→B $_{R-free}$	-	46	63	58	19	47	87	0

0 100

Table 7. *Comparison of R-work/R-free (rounded to two decimal places) for the models generated from the synthetic HA-NCS data sets. Each row shows the percentage of models that a pipeline variant built with equal R-work or R-free to each other pipeline variant.*

Pipeline variant	A	A→P^*	A→B	B	B→P^*	P^*	P^*→A	P^*→B
A $_{R-work}$	100	4	1	1	3	0	12	1
A $_{R-free}$	-	-	-	-	-	-	-	-
A→P^* $_{R-work}$	4	100	3	2	6	0	2	3
A→P^* $_{R-free}$	-	100	5	6	4	5	3	6
A→B $_{R-work}$	1	3	100	13	3	5	1	13
A→B $_{R-free}$	-	5	100	9	4	5	4	8
B $_{R-work}$	1	2	13	100	4	4	1	11
B $_{R-free}$	-	6	9	100	4	6	4	8
B→P^* $_{R-work}$	3	6	3	4	100	7	3	6
B→P^* $_{R-free}$	-	4	4	4	100	6	1	4
P^* $_{R-work}$	0	0	5	4	7	100	0	6
P^* $_{R-free}$	-	5	5	6	6	100	1	7
P^*→A $_{R-work}$	12	2	1	1	3	0	100	1
P^*→A $_{R-free}$	-	3	4	4	1	1	100	2
P^*→B $_{R-work}$	1	3	13	11	6	6	1	100
P^*→B $_{R-free}$	-	6	8	8	4	7	2	100

0 100

Table 8. *Comparison of R-work/R-free (rounded to two decimal places) for the models generated from the synthetic HA-NCS data sets. Each row shows the percentage of models that a pipeline variant built with R-work or R-free at least 5% lower than each other pipeline variant.*

Pipeline variant	A	A→P^*	A→B	B	B→P^*	P^*	P^*⟶A	P^*⟶B
A $_{R-work}$	0	53	85	85	71	93	3	82
A $_{R-free}$	-	-	-	-	-	-	-	-
A→P^* $_{R-work}$	6	0	75	72	39	85	0	67
A→P^* $_{R-free}$	-	0	38	36	16	32	78	29
A→B $_{R-work}$	2	1	0	9	1	17	0	7
A→B $_{R-free}$	-	17	0	15	2	18	63	9
B $_{R-work}$	2	1	13	0	0	20	0	8
B $_{R-free}$	-	19	17	0	1	19	66	12
B→P^* $_{R-work}$	4	4	70	67	0	45	0	51
B→P^* $_{R-free}$	-	47	62	63	0	47	90	49
P^* $_{R-work}$	0	0	41	38	0	0	0	27
P^* $_{R-free}$	-	22	37	34	4	0	84	24
P^*⟶A $_{R-work}$	19	69	91	91	81	99	0	89
P^*⟶A $_{R-free}$	-	2	6	7	1	2	0	3
P^*⟶B $_{R-work}$	2	2	26	23	2	25	0	0
P^*⟶B $_{R-free}$	-	26	28	26	5	25	73	0

0 99

Table 9. *Comparison of R-work/R-free (rounded to two decimal places) for the models generated from the synthetic HA-NCS data sets. Each row shows the percentage of models that a pipeline variant built with R-work or R-free between 1% and 4% lower than each other pipeline variant.*

Pipeline variant	A	A→P^*	A→B	B	B→P^*	P^*	P^*⟶A	P^*⟶B
A $_{R-work}$	0	29	9	8	14	4	25	9
A $_{R-free}$	-	-	-	-	-	-	-	-
A→P^* $_{R-work}$	7	0	14	17	32	14	3	19
A→P^* $_{R-free}$	-	0	21	20	13	23	12	19
A→B $_{R-work}$	3	7	0	27	6	16	2	16
A→B $_{R-free}$	-	19	0	28	7	18	17	20
B $_{R-work}$	4	8	37	0	4	17	1	21
B $_{R-free}$	-	19	31	0	4	19	14	22
B→P^* $_{R-work}$	7	19	20	25	0	37	3	30
B→P^* $_{R-free}$	-	20	25	28	0	29	6	28
P^* $_{R-work}$	2	0	21	20	10	0	0	21
P^* $_{R-free}$	-	18	22	22	15	0	10	23
P^*⟶A $_{R-work}$	40	27	7	7	13	1	0	8
P^*⟶A $_{R-free}$	-	5	10	9	1	3	0	7
P^*⟶B $_{R-work}$	6	10	39	38	12	20	2	0
P^*⟶B $_{R-free}$	-	20	35	32	14	22	14	0

0 40

Table 10. *Structure completeness comparison for the models generated from the synthetic MR-NCS data sets. Each row corresponds to a pipeline variant, and shows the percentage (rounded to the nearest integer) of models that the pipeline variant built with higher structure completeness than each of the other pipeline variants.*

Pipeline variant	A	A→P^*	A→B	B	B→P^*	P^*	P^*⟶A	P^*⟶B
A	0	1	1	1	0	2	30	1
A→P^*	96	0	15	12	3	24	96	6
A→B	94	81	0	43	32	74	94	28
B	95	85	48	0	24	76	95	33
B→P^*	99	95	65	69	0	86	99	48
P^*	97	69	24	22	10	0	97	13
P^*⟶A	16	1	1	0	0	2	0	1
P^*⟶B	97	91	67	61	48	84	97	0

0 99

Table 11. *Structure completeness comparison for the models generated from the synthetic MR-NCS data sets. Each row corresponds to a pipeline variant, and shows the percentage (rounded to the nearest integer) of models that the pipeline variant built with equal structure completeness to each of the other pipeline variants.*

Pipeline variant	A	A→P^*	A→B	B	B→P^*	P^*	P^*⟶A	P^*⟶B
A	100	3	6	5	1	1	54	2
A→P^*	3	100	4	4	1	7	3	2
A→B	6	4	100	9	4	2	6	5
B	5	4	9	100	7	2	4	6
B→P^*	1	1	4	7	100	4	1	4
P^*	1	7	2	2	4	100	1	2
P^*⟶A	54	3	6	4	1	1	100	2
P^*⟶B	2	2	5	6	4	2	2	100

1 100

Table 12. *Structure completeness comparison for the models generated from the synthetic MR-NCS data sets. Each row corresponds to a pipeline variant, and shows the percentage (rounded to the nearest integer) of models that the pipeline variant built with at least 5% higher structure completeness than each of the other pipeline variants.*

Pipeline variant	A	A→P^*	A→B	B	B→P^*	P^*	P^*⟶A	P^*⟶B
A	0	0	0	0	0	2	7	0
A→P^*	78	0	7	5	1	14	79	3
A→B	83	75	0	31	23	71	83	20
B	86	78	34	0	13	72	86	22
B→P^*	94	89	51	46	0	79	95	33
P^*	92	46	17	15	4	0	93	8
P^*⟶A	3	0	0	0	0	1	0	0
P^*⟶B	92	86	52	48	39	81	92	0

0 95

Table 13. *Structure completeness comparison for the models generated from the synthetic MR-NCS data sets. Each row corresponds to a pipeline variant, and shows the percentage (rounded to the nearest integer) of models that the pipeline variant built with between 1% and 4% higher structure completeness than each of the other pipeline variants.*

Pipeline variant	A	A→P^*	A→B	B	B→P^*	P^*	P^*→A	P^*→B
A	0	0	1	0	0	0	24	0
A→P^*	18	0	8	6	2	10	17	4
A→B	11	6	0	12	9	4	11	9
B	9	6	14	0	11	4	9	11
B→P^*	5	6	13	23	0	8	4	15
P^*	5	23	7	7	6	0	4	5
P^*→A	13	0	1	0	0	0	0	1
P^*→B	5	5	15	13	10	4	5	0

0 24

Table 14. *Comparison of R-work/R-free (rounded to two decimal places) for the models generated from the synthetic MR-NCS data sets. Each row shows the percentage of models that a pipeline variant built with lower R-work or R-free than each other pipeline variant.*

Pipeline variant	A	A→P^*	A→B	B	B→P^*	P^*	P^*→A	P^*→B
A $_{R-work}$	0	83	93	93	85	98	30	92
A $_{R-free}$	-	-	-	-	-	-	-	-
A→P^* $_{R-work}$	13	0	88	88	68	99	3	83
A→P^* $_{R-free}$	-	0	58	56	28	55	90	49
A→B $_{R-work}$	6	8	0	41	7	37	2	27
A→B $_{R-free}$	-	35	0	47	10	39	79	34
B $_{R-work}$	5	9	46	0	4	40	1	32
B $_{R-free}$	-	38	44	0	6	40	81	34
B→P^* $_{R-work}$	11	25	90	92	0	83	4	81
B→P^* $_{R-free}$	-	68	86	90	0	74	96	77
P^* $_{R-work}$	2	0	57	56	11	0	0	49
P^* $_{R-free}$	-	40	57	54	19	0	95	44
P^*→A $_{R-work}$	59	94	97	97	94	100	0	97
P^*→A $_{R-free}$	-	7	17	15	3	4	0	11
P^*→B $_{R-work}$	7	13	60	59	14	45	1	0
P^*→B $_{R-free}$	-	47	59	58	17	46	86	0

0 100

Table 15. *Comparison of R-work/R-free (rounded to two decimal places) for the models generated from the synthetic MR-NCS data sets. Each row shows the percentage of models that a pipeline variant built with equal R-work or R-free to each other pipeline variant.*

Pipeline variant	A	A→P^*	A→B	B	B→P^*	P^*	P^*→A	P^*→B
A $_{R-work}$	100	4	1	2	4	0	12	2
A $_{R-free}$	-	-	-	-	-	-	-	-
A→P^* $_{R-work}$	4	100	3	3	7	1	3	4
A→P^* $_{R-free}$	-	100	6	6	4	5	2	5
A→B $_{R-work}$	1	3	100	13	4	6	1	13
A→B $_{R-free}$	-	6	100	9	4	4	4	8
B $_{R-work}$	2	3	13	100	3	4	1	10
B $_{R-free}$	-	6	9	100	4	5	4	8
B→P^* $_{R-work}$	4	7	4	3	100	7	2	5
B→P^* $_{R-free}$	-	4	4	4	100	7	1	5
P^* $_{R-work}$	0	1	6	4	7	100	0	6
P^* $_{R-free}$	-	5	4	5	7	100	1	9
P^*→A $_{R-work}$	12	3	1	1	2	0	100	1
P^*→A $_{R-free}$	-	2	4	4	1	1	100	3
P^*→B $_{R-work}$	2	4	13	10	5	6	1	100
P^*→B $_{R-free}$	-	5	8	8	5	9	3	100

0 100

Table 16. *Comparison of R-work/R-free (rounded to two decimal places) for the models generated from the synthetic MR-NCS data sets. Each row shows the percentage of models that a pipeline variant built with R-work or R-free at least 5% lower than each other pipeline variant.*

Pipeline variant	A	A→P^*	A→B	B	B→P^*	P^*	P^*→A	P^*→B
A $_{R-work}$	0	51	84	86	68	93	4	81
A $_{R-free}$	-	-	-	-	-	-	-	-
A→P^* $_{R-work}$	5	0	73	71	39	85	0	65
A→P^* $_{R-free}$	-	0	36	36	15	32	81	28
A→B $_{R-work}$	2	1	0	10	1	19	0	9
A→B $_{R-free}$	-	18	0	16	3	20	65	11
B $_{R-work}$	2	1	12	0	0	21	0	9
B $_{R-free}$	-	20	18	0	1	21	66	11
B→P^* $_{R-work}$	3	4	66	64	0	48	0	51
B→P^* $_{R-free}$	-	48	59	60	0	47	90	46
P^* $_{R-work}$	0	0	38	37	1	0	0	26
P^* $_{R-free}$	-	21	32	33	4	0	85	22
P^*→A $_{R-work}$	19	68	90	91	79	99	0	88
P^*→A $_{R-free}$	-	2	7	6	1	1	0	3
P^*→B $_{R-work}$	2	2	24	21	2	26	0	0
P^*→B $_{R-free}$	-	26	28	26	4	26	74	0

0 99

Table 17. *Comparison of R-work/R-free (rounded to two decimal places) for the models generated from the synthetic MR-NCS data sets. Each row shows the percentage of models that a pipeline variant built with R-work or R-free between 1% and 4% lower than each other pipeline variant.*

Pipeline variant	A	A→P^*	A→B	B	B→P^*	P^*	P^*→A	P^*→B
A $_{R-work}$	0	32	9	8	17	4	26	11
A $_{R-free}$	-	-	-	-	-	-	-	-
A→P^* $_{R-work}$	8	0	15	17	29	14	3	18
A→P^* $_{R-free}$	-	0	22	20	12	23	10	21
A→B $_{R-work}$	3	7	0	31	6	18	2	18
A→B $_{R-free}$	-	17	0	31	8	19	14	23
B $_{R-work}$	4	8	33	0	4	18	1	23
B $_{R-free}$	-	18	26	0	5	19	15	23
B→P^* $_{R-work}$	8	21	24	29	0	34	4	30
B→P^* $_{R-free}$	-	21	27	30	0	27	6	32
P^* $_{R-work}$	2	0	19	19	10	0	0	23
P^* $_{R-free}$	-	18	25	21	15	0	10	22
P^*→A $_{R-work}$	40	27	7	6	15	1	0	10
P^*→A $_{R-free}$	-	5	10	9	2	3	0	8
P^*→B $_{R-work}$	4	11	36	37	11	19	1	0
P^*→B $_{R-free}$	-	21	31	32	13	21	13	0

0 40

Table 18. *Structure completeness comparison for the models generated from the synthetic NO-NCS data sets. Each row corresponds to a pipeline variant, and shows the percentage (rounded to the nearest integer) of models that the pipeline variant built with higher structure completeness than each of the other pipeline variants.*

Pipeline variant	A	A→P^*	A→P	A→B	B	B→P^*	B→P	P^*	P^*→A	P^*→B	P	P→A	P→B
A	0	1	1	1	1	0	0	2	27	0	2	25	0
A→P^*	95	0	40	20	16	3	3	22	96	6	22	96	7
A→P	96	50	0	25	20	4	4	20	96	8	21	96	8
A→B	92	74	71	0	40	27	24	65	92	24	65	93	25
B	94	79	76	50	0	23	20	68	94	27	69	95	29
B→P^*	99	95	94	70	72	0	34	83	99	45	84	99	48
B→P	99	96	94	71	73	49	0	85	99	45	86	99	50
P^*	97	72	73	32	29	12	11	0	97	16	43	98	16
P^*→A	16	1	0	1	1	0	0	2	0	0	2	19	1
P^*→B	97	91	88	71	68	51	50	81	97	0	81	98	50
P	97	74	74	33	28	11	10	46	97	16	0	97	17
P→A	14	1	1	0	1	0	0	1	18	0	1	0	0
P→B	97	89	88	69	65	47	46	81	97	45	81	97	0

0 99

Table 19. *Structure completeness comparison for the models generated from the synthetic NO-NCS data sets. Each row corresponds to a pipeline variant, and shows the percentage (rounded to the nearest integer) of models that the pipeline variant built with equal structure completeness to each of the other pipeline variants.*

Pipeline variant	A	A→P^*	A→P	A→B	B	B→P^*	B→P	P^*	P^*→A	P^*→B	P	P→A	P→B
A	100	4	3	7	5	1	1	1	57	3	1	61	3
A→P^*	4	100	10	6	5	2	2	6	4	3	4	3	4
A→P	3	10	100	4	4	2	2	7	3	4	5	3	4
A→B	7	6	4	100	10	4	4	3	6	5	2	7	6
B	5	5	4	10	100	6	6	3	5	6	3	5	6
B→P^*	1	2	2	4	6	100	17	5	1	4	5	1	5
B→P	1	2	2	4	6	17	100	4	1	4	4	1	4
P^*	1	6	7	3	3	5	4	100	1	3	12	1	3
P^*→A	57	4	3	6	5	1	1	1	100	2	1	63	2
P^*→B	3	3	4	5	6	4	4	3	2	100	3	2	5
P	1	4	5	2	3	5	4	12	1	3	100	1	2
P→A	61	3	3	7	5	1	1	1	63	2	1	100	3
P→B	3	4	4	6	6	5	4	3	2	5	2	3	100

1 100

Table 20. *Structure completeness comparison for the models generated from the synthetic NO-NCS data sets. Each row corresponds to a pipeline variant, and shows the percentage (rounded to the nearest integer) of models that the pipeline variant built with at least 5% higher structure completeness than each of the other pipeline variants.*

Pipeline variant	A	A→P^*	A→P	A→B	B	B→P^*	B→P	P^*	P^*→A	P^*→B	P	P→A	P→B
A	0	1	0	0	1	0	0	2	6	0	2	6	0
A→P^*	75	0	21	9	7	1	1	13	76	2	11	76	2
A→P	84	26	0	13	10	1	1	11	84	3	11	84	4
A→B	79	67	65	0	27	18	17	61	79	16	61	80	18
B	82	71	70	33	0	14	11	63	82	18	63	82	19
B→P^*	94	88	85	55	53	0	11	74	95	31	75	94	34
B→P	94	88	86	59	55	16	0	76	94	33	75	94	36
P^*	92	50	47	24	21	4	3	0	93	9	15	93	9
P^*→A	2	0	0	0	0	0	0	1	0	0	2	3	0
P^*→B	90	84	82	56	54	40	38	77	91	0	76	91	37
P	92	49	49	24	21	4	3	16	92	9	0	93	9
P→A	2	0	1	0	0	0	0	1	2	0	1	0	0
P→B	90	83	81	55	49	36	35	76	90	33	75	90	0

0 95

Table 21. *Structure completeness comparison for the models generated from the synthetic NO-NCS data sets. Each row corresponds to a pipeline variant, and shows the percentage (rounded to the nearest integer) of models that the pipeline variant built with between 1% and 4% higher structure completeness than each of the other pipeline variants.*

Pipeline variant	A	A→P^*	A→P	A→B	B	B→P^*	B→P	P^*	P^*→A	P^*→B	P	P→A	P→B
A	0	0	0	1	1	0	0	0	21	0	0	18	0
A→P^*	20	0	19	11	9	2	2	9	20	4	11	20	4
A→P	12	24	0	12	9	3	3	9	12	5	10	12	5
A→B	13	8	5	0	13	9	8	3	13	8	5	13	7
B	12	8	7	17	0	8	9	5	12	8	6	13	10
B→P^*	5	7	9	14	18	0	23	9	4	14	9	4	14
B→P	5	8	8	13	18	33	0	9	5	13	11	5	14
P^*	5	22	26	8	8	8	8	0	5	8	27	4	7
P^*→A	14	0	0	1	1	0	0	0	0	0	0	16	1
P^*→B	6	7	6	15	14	11	13	4	7	0	5	7	13
P	5	25	25	9	8	7	7	29	5	7	0	5	9
P→A	12	1	0	0	1	0	0	0	16	0	0	0	0
P→B	7	6	7	14	16	11	11	5	7	12	5	7	0

0 33

Table 22. *Comparison of R-work/R-free (rounded to two decimal places) for the models generated from the synthetic NO-NCS data sets. Each row shows the percentage of models that a pipeline variant built with lower R-work or R-free than each other pipeline variant.*

Pipeline variant	A	A→P^*	A→P	A→B	B	B→P^*	B→P	P^*	P^*→A	P^*→B	P	P→A	P→B
A $_{R-work}$	0	82	82	94	95	87	87	97	29	92	97	28	93
A $_{R-free}$	-	-	-	-	-	-	-	-	-	-	-	-	-
A→P^* $_{R-work}$	13	0	34	89	91	73	74	99	3	86	99	4	87
A→P^* $_{R-free}$	-	0	42	63	62	30	29	54	89	53	52	92	55
A→P $_{R-work}$	14	39	0	91	91	74	75	99	5	87	99	5	87
A→P $_{R-free}$	-	44	0	64	63	31	30	55	91	55	53	92	55
A→B $_{R-work}$	5	7	7	0	40	6	6	30	1	22	29	2	26
A→B $_{R-free}$	-	31	30	0	46	8	7	32	75	31	31	78	31
B $_{R-work}$	4	7	7	46	0	3	3	32	1	26	31	2	29
B $_{R-free}$	-	30	31	43	0	4	4	34	75	32	33	77	31
B→P^* $_{R-work}$	10	21	21	92	93	0	34	79	3	81	79	4	82
B→P^* $_{R-free}$	-	66	64	88	92	0	41	73	95	80	73	97	82
B→P $_{R-work}$	10	21	20	91	94	32	0	77	4	83	77	4	82
B→P $_{R-free}$	-	66	65	90	92	42	0	71	96	80	73	97	82
P^* $_{R-work}$	3	0	1	66	64	12	14	0	0	54	33	0	56
P^* $_{R-free}$	-	41	41	65	62	21	21	0	94	53	41	95	52
P^*→A $_{R-work}$	59	94	93	98	98	95	95	100	0	97	100	38	98
P^*→A $_{R-free}$	-	7	6	21	21	3	3	5	0	14	6	47	14
P^*→B $_{R-work}$	6	11	9	66	65	13	13	40	2	0	39	2	48
P^*→B $_{R-free}$	-	42	40	62	62	16	15	40	83	0	40	84	49
P $_{R-work}$	2	0	0	67	64	13	14	36	0	56	0	0	55
P $_{R-free}$	-	41	41	65	63	21	21	43	93	53	0	94	53
P→A $_{R-work}$	59	94	94	98	98	95	95	100	38	98	100	0	97
P→A $_{R-free}$	-	6	6	18	19	2	2	4	41	12	5	0	13
P→B $_{R-work}$	5	10	10	64	60	14	12	40	1	41	40	1	0
P→B $_{R-free}$	-	40	39	61	61	14	13	41	82	44	40	83	0

0 100

Table 23. *Comparison of R-work/R-free (rounded to two decimal places) for the models generated from the synthetic NO-NCS data sets. Each row shows the percentage of models that a pipeline variant built with equal R-work or R-free to each other pipeline variant.*

Pipeline variant	A	A→P^*	A→P	A→B	B	B→P^*	B→P	P^*	P^*⟶A	P^*⟶B	P	P→A	P→B
A $_{R-work}$	100	5	4	1	1	3	3	0	12	1	1	13	2
A $_{R-free}$	-	-	-	-	-	-	-	-	-	-	-	-	-
A→P^* $_{R-work}$	5	100	27	3	2	5	6	0	2	3	0	2	3
A→P^* $_{R-free}$	-	100	14	6	8	4	5	5	3	5	7	2	5
A→P $_{R-work}$	4	27	100	2	2	5	5	0	2	4	0	1	3
A→P $_{R-free}$	-	14	100	6	6	5	5	4	3	5	6	1	5
A→B $_{R-work}$	1	3	2	100	14	3	3	4	1	12	4	1	10
A→B $_{R-free}$	-	6	6	100	11	3	3	3	5	7	4	4	8
B $_{R-work}$	1	2	2	14	100	3	3	4	1	9	5	1	11
B $_{R-free}$	-	8	6	11	100	4	3	4	4	7	4	4	8
B→P^* $_{R-work}$	3	5	5	3	3	100	34	9	1	5	8	1	5
B→P^* $_{R-free}$	-	4	5	3	4	100	17	7	2	4	6	1	5
B→P $_{R-work}$	3	6	5	3	3	34	100	9	1	4	9	1	6
B→P $_{R-free}$	-	5	5	3	3	17	100	9	1	5	7	0	5
P^* $_{R-work}$	0	0	0	4	4	9	9	100	0	5	31	0	4
P^* $_{R-free}$	-	5	4	3	4	7	9	100	1	7	16	1	8
P^*⟶A $_{R-work}$	12	2	2	1	1	1	1	0	100	1	0	24	1
P^*⟶A $_{R-free}$	-	3	3	5	4	2	1	1	100	2	2	12	4
P^*⟶B $_{R-work}$	1	3	4	12	9	5	4	5	1	100	5	1	11
P^*⟶B $_{R-free}$	-	5	5	7	7	4	5	7	2	100	7	3	7
P $_{R-work}$	1	0	0	4	5	8	9	31	0	5	100	0	5
P $_{R-free}$	-	7	6	4	4	6	7	16	2	7	100	1	6
P→A $_{R-work}$	13	2	1	1	1	1	1	0	24	1	0	100	1
P→A $_{R-free}$	-	2	1	4	4	1	0	1	12	3	1	100	4
P→B $_{R-work}$	2	3	3	10	11	5	6	4	1	11	5	1	100
P→B $_{R-free}$	-	5	5	8	8	5	5	8	4	7	6	4	100

0 100

Table 24. *Comparison of R-work/R-free (rounded to two decimal places) for the models generated from the synthetic NO-NCS data sets. Each row shows the percentage of models that a pipeline variant built with R-work or R-free at least 5% lower than each other pipeline variant.*

Pipeline variant	A	A→P^*	A→P	A→B	B	B→P^*	B→P	P^*	P^*→A	P^*→B	P	P→A	P→B
A $_{R-work}$	0	54	53	86	88	73	72	93	4	84	92	4	84
A $_{R-free}$	-	-	-	-	-	-	-	-	-	-	-	-	-
A→P^* $_{R-work}$	5	0	0	77	77	44	44	85	1	69	83	1	71
A→P^* $_{R-free}$	-	0	7	42	41	16	16	32	77	34	33	78	37
A→P $_{R-work}$	5	1	0	77	76	47	45	88	1	70	86	1	71
A→P $_{R-free}$	-	10	0	43	42	18	16	33	79	36	34	80	38
A→B $_{R-work}$	2	1	1	0	9	1	1	16	0	7	16	0	8
A→B $_{R-free}$	-	14	15	0	15	2	2	15	59	9	16	57	11
B $_{R-work}$	2	1	1	12	0	0	0	16	0	6	16	0	8
B $_{R-free}$	-	15	14	16	0	1	1	16	59	10	15	60	11
B→P^* $_{R-work}$	3	3	3	71	72	0	1	40	0	54	40	1	58
B→P^* $_{R-free}$	-	44	44	67	68	0	5	42	88	50	41	89	52
B→P $_{R-work}$	4	4	3	72	70	1	0	41	0	55	40	1	57
B→P $_{R-free}$	-	45	43	68	67	5	0	43	89	51	43	90	53
P^* $_{R-work}$	0	0	0	48	47	1	1	0	0	32	1	0	36
P^* $_{R-free}$	-	23	22	42	43	4	5	0	84	29	6	84	33
P^*→A $_{R-work}$	19	68	67	91	92	83	82	99	0	90	99	5	90
P^*→A $_{R-free}$	-	2	1	9	8	1	1	2	0	5	2	12	5
P^*→B $_{R-work}$	2	2	2	25	24	3	3	23	0	0	22	0	14
P^*→B $_{R-free}$	-	23	22	29	29	5	4	22	69	0	22	70	18
P $_{R-work}$	1	0	0	49	47	0	1	1	0	33	0	0	34
P $_{R-free}$	-	22	21	43	43	5	5	7	84	29	0	84	31
P→A $_{R-work}$	21	67	68	91	92	82	81	99	5	90	99	0	91
P→A $_{R-free}$	-	3	2	7	6	1	1	1	9	4	1	0	5
P→B $_{R-work}$	3	2	1	26	24	2	2	21	0	12	19	0	0
P→B $_{R-free}$	-	22	21	28	27	3	3	19	67	15	19	69	0

0 99

Table 25. *Comparison of R-work/R-free (rounded to two decimal places) for the models generated from the synthetic NO-NCS data sets. Each row shows the percentage of models that a pipeline variant built with R-work or R-free between 1% and 4% lower than each other pipeline variant.*

Pipeline variant	A	A→P^*	A→P	A→B	B	B→P^*	B→P	P^*	P^*→A	P^*→B	P	P→A	P→B
A $_{R-work}$	0	28	29	8	7	14	15	4	26	9	5	24	9
A $_{R-free}$	-	-	-	-	-	-	-	-	-	-	-	-	-
A→P^* $_{R-work}$	8	0	34	12	14	29	29	15	3	17	16	4	16
A→P^* $_{R-free}$	-	0	35	21	21	14	13	22	12	19	19	14	18
A→P $_{R-work}$	9	38	0	14	16	27	29	11	4	17	13	4	16
A→P $_{R-free}$	-	34	0	21	21	13	14	23	12	18	20	12	18
A→B $_{R-work}$	3	6	6	0	31	5	5	14	1	16	13	1	18
A→B $_{R-free}$	-	17	15	0	32	6	5	16	16	22	16	21	20
B $_{R-work}$	3	6	6	34	0	3	3	16	1	19	16	1	22
B $_{R-free}$	-	15	17	26	0	3	3	18	16	22	18	17	20
B→P^* $_{R-work}$	7	18	18	20	22	0	33	39	3	27	40	3	24
B→P^* $_{R-free}$	-	21	20	22	23	0	36	31	7	30	31	8	30
B→P $_{R-work}$	6	17	17	19	24	32	0	36	3	28	37	3	25
B→P $_{R-free}$	-	21	21	22	25	36	0	27	7	29	30	7	29
P^* $_{R-work}$	2	0	1	18	17	11	13	0	0	22	32	0	20
P^* $_{R-free}$	-	17	19	23	19	16	15	0	10	24	34	10	19
P^*→A $_{R-work}$	40	26	26	7	6	12	13	1	0	7	1	33	7
P^*→A $_{R-free}$	-	5	5	12	13	2	2	3	0	10	4	35	9
P^*→B $_{R-work}$	4	8	8	41	41	10	10	18	2	0	17	1	34
P^*→B $_{R-free}$	-	19	18	33	33	11	12	18	14	0	18	14	31
P $_{R-work}$	1	0	0	18	17	12	13	35	0	23	0	0	20
P $_{R-free}$	-	19	20	22	20	16	16	36	9	24	0	10	22
P→A $_{R-work}$	38	27	26	7	5	13	14	1	34	8	1	0	7
P→A $_{R-free}$	-	3	5	11	13	1	2	3	32	8	4	0	8
P→B $_{R-work}$	2	8	8	38	36	11	10	20	1	29	21	1	0
P→B $_{R-free}$	-	18	18	33	34	11	10	22	15	29	21	15	0

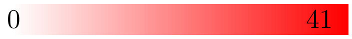

Appendix C
The Results of the Original data sets Used in Buccaneer Development

Table 1. *Complete and intermediate models produced by the 23 pipeline variants for the 52 original data sets, where (T) and (C) denote intermediate models produced by pipeline executions that timed out and crashed, respectively.*

Pipeline variant	HA-NCS			MR-NCS			NO-NCS		
	Complete	Intermediate	Failed	Complete	Intermediate	Failed	Complete	Intermediate	Failed
A	52	0(T) 0(C)	0	52	0(T) 0(C)	0	52	0(T) 0(C)	0
A→P^*	51	1(T) 0(C)	0	52	0(T) 0(C)	0	52	0(T) 0(C)	0
A→B	52	0(T) 0(C)	0	52	0(T) 0(C)	0	52	0(T) 0(C)	0
B	52	0(T) 0(C)	0	52	0(T) 0(C)	0	52	0(T) 0(C)	0
B→P^*	51	0(T) 0(C)	1	51	0(T) 0(C)	1	50	1(T) 0(C)	1
P^*	51	1(T) 0(C)	0	52	0(T) 0(C)	0	52	0(T) 0(C)	0
$P^* \longrightarrow A$	52	0(T) 0(C)	0	52	0(T) 0(C)	0	52	0(T) 0(C)	0
$P^* \longrightarrow B$	52	0(T) 0(C)	0	52	0(T) 0(C)	0	52	0(T) 0(C)	0
S^*	52	0(T) 0(C)	0	52	0(T) 0(C)	0	52	0(T) 0(C)	0
$S^* \longrightarrow A$	52	0(T) 0(C)	0	52	0(T) 0(C)	0	52	0(T) 0(C)	0
$S^* \longrightarrow B$	52	0(T) 0(C)	0	52	0(T) 0(C)	0	52	0(T) 0(C)	0
$S^* \longrightarrow P^*$	52	0(T) 0(C)	0	51	1(T) 0(C)	0	52	0(T) 0(C)	0
A→P	-	-	-	-	-	-	52	0(T) 0(C)	0
B→P	-	-	-	-	-	-	51	0(T) 0(C)	1
P	-	-	-	-	-	-	52	0(T) 0(C)	0
P→A	-	-	-	-	-	-	52	0(T) 0(C)	0
P→B	-	-	-	-	-	-	52	0(T) 0(C)	0
S	-	-	-	-	-	-	52	0(T) 0(C)	0
S→A	-	-	-	-	-	-	52	0(T) 0(C)	0
S→B	-	-	-	-	-	-	52	0(T) 0(C)	0
$S^* \longrightarrow P$	-	-	-	-	-	-	52	0(T) 0(C)	0
S$\longrightarrow P^*$	-	-	-	-	-	-	52	0(T) 0(C)	0
S→P	-	-	-	-	-	-	52	0(T) 0(C)	0

Models used in the comparison: 51 HA-NCS, 51 MR-NCS and 51 NO-NCS.

Table 2. *Structure completeness comparison for the models generated from the 52 original HA-NCS data sets. Each row corresponds to a pipeline variant, and shows the percentage (rounded to the nearest integer) of models that the pipeline variant built with higher structure completeness than each of the other pipeline variants.*

Pipeline variant	A	A→P^*	A→B	B	B→P^*	P^*	P^*→A	P^*→B	S^*	S^*→A	S^*→B	S^*→P^*
A	0	20	18	27	24	29	35	14	71	53	29	35
A→P^*	63	0	22	47	33	45	61	14	94	71	39	57
A→B	69	67	0	57	45	69	73	31	92	80	61	71
B	59	43	31	0	20	43	49	22	90	69	41	55
B→P^*	61	53	41	67	0	63	55	29	94	75	49	75
P^*	57	29	27	45	25	0	47	14	82	65	39	45
P^*→A	45	14	20	37	27	35	0	12	82	61	27	49
P^*→B	80	67	45	71	55	76	76	0	96	80	65	82
S^*	25	2	8	8	2	10	14	4	0	41	6	8
S^*→A	20	12	14	18	16	29	20	12	55	0	14	29
S^*→B	59	45	25	47	31	51	51	22	94	75	0	55
S^*→P^*	51	31	25	43	16	29	39	18	86	65	35	0

0 … 96

Table 3. *Structure completeness comparison for the models generated from the 52 original HA-NCS data sets. Each row corresponds to a pipeline variant, and shows the percentage (rounded to the nearest integer) of models that the pipeline variant built with equal structure completeness to each of the other pipeline variants.*

Pipeline variant	A	A→P^*	A→B	B	B→P^*	P^*	P^*→A	P^*→B	S^*	S^*→A	S^*→B	S^*→P^*
A	100	18	14	14	16	14	20	6	4	27	12	14
A→P^*	18	100	12	10	14	25	25	20	4	18	16	12
A→B	14	12	100	12	14	4	8	24	0	6	14	4
B	14	10	12	100	14	12	14	8	2	14	12	2
B→P^*	16	14	14	14	100	12	18	16	4	10	20	10
P^*	14	25	4	12	12	100	18	10	8	6	10	25
P^*→A	20	25	8	14	18	18	100	12	4	20	22	12
P^*→B	6	20	24	8	16	10	12	100	0	8	14	0
S^*	4	4	0	2	4	8	4	0	100	4	0	6
S^*→A	27	18	6	14	10	6	20	8	4	100	12	6
S^*→B	12	16	14	12	20	10	22	14	0	12	100	10
S^*→P^*	14	12	4	2	10	25	12	0	6	6	10	100

0 … 100

Table 4. *Structure completeness comparison for the models generated from the 52 original HA-NCS data sets. Each row corresponds to a pipeline variant, and shows the percentage (rounded to the nearest integer) of models that the pipeline variant built with at least 5% higher structure completeness than each of the other pipeline variants.*

Pipeline variant	A	A→P^*	A→B	B	B→P^*	P^*	P^*→A	P^*→B	S^*	S^*→A	S^*→B	S^*→P^*
A	0	0	10	14	10	14	12	10	57	33	6	14
A→P^*	35	0	14	25	12	12	27	10	78	43	18	25
A→B	29	18	0	22	16	29	29	12	75	49	24	33
B	35	18	18	0	12	22	25	10	75	43	14	24
B→P^*	41	25	22	29	0	25	31	16	75	47	25	33
P^*	29	14	20	22	8	0	25	10	69	39	22	16
P^*→A	20	4	14	24	8	14	0	6	69	37	12	25
P^*→B	37	29	22	27	22	37	37	0	80	49	31	43
S^*	16	0	4	2	0	8	8	4	0	35	4	4
S^*→A	10	0	6	4	6	8	6	6	41	0	4	14
S^*→B	35	18	10	16	10	20	25	12	80	43	0	31
S^*→P^*	33	12	16	22	10	14	24	10	73	43	16	0

0 80

Table 5. *Structure completeness comparison for the models generated from the 52 original HA-NCS data sets. Each row corresponds to a pipeline variant, and shows the percentage (rounded to the nearest integer) of models that the pipeline variant built with between 1% and 4% higher structure completeness than each of the other pipeline variants.*

Pipeline variant	A	A→P^*	A→B	B	B→P^*	P^*	P^*→A	P^*→B	S^*	S^*→A	S^*→B	S^*→P^*
A	0	20	8	14	14	16	24	4	14	20	24	22
A→P^*	27	0	8	22	22	33	33	4	16	27	22	31
A→B	39	49	0	35	29	39	43	20	18	31	37	37
B	24	25	14	0	8	22	24	12	16	25	27	31
B→P^*	20	27	20	37	0	37	24	14	20	27	24	41
P^*	27	16	8	24	18	0	22	4	14	25	18	29
P^*→A	25	10	6	14	20	22	0	6	14	24	16	24
P^*→B	43	37	24	43	33	39	39	0	16	31	33	39
S^*	10	2	4	6	2	2	6	0	0	6	2	4
S^*→A	10	12	8	14	10	22	14	6	14	0	10	16
S^*→B	24	27	16	31	22	31	25	10	14	31	0	24
S^*→P^*	18	20	10	22	6	16	16	8	14	22	20	0

0 49

Table 6. *Comparison of R-work/R-free (rounded to two decimal places) for the models generated from the 52 original HA-NCS data sets. Each row shows the percentage of models that a pipeline variant built with lower R-work or R-free than each other pipeline variant.*

Pipeline variant	A	A→P^*	A→B	B	B→P^*	P^*	P^*⟶A	P^*⟶B	S^*	S^*⟶A	S^*⟶B	S^*⟶P^*
A $_{R-work}$	0	25	92	96	39	51	31	86	100	49	94	47
A $_{R-free}$	-	-	-	-	-	-	-	-	-	-	-	-
A→P^* $_{R-work}$	57	0	98	98	51	73	51	92	100	71	100	76
A→P^* $_{R-free}$	-	0	86	90	43	61	80	82	-	90	92	63
A→B $_{R-work}$	4	2	0	53	2	6	4	25	98	4	53	4
A→B $_{R-free}$	-	12	0	47	4	6	24	24	-	45	53	4
B $_{R-work}$	2	0	22	0	0	0	2	12	100	2	27	2
B $_{R-free}$	-	8	29	0	2	6	14	25	-	43	37	6
B→P^* $_{R-work}$	41	27	86	98	0	45	39	90	100	57	96	57
B→P^* $_{R-free}$	-	43	90	96	0	47	69	86	-	76	94	53
P^* $_{R-work}$	35	12	86	98	18	0	27	88	100	45	96	31
P^* $_{R-free}$	-	24	92	90	31	0	67	84	-	82	98	35
P^*⟶A $_{R-work}$	43	31	96	96	45	57	0	94	100	51	98	59
P^*⟶A $_{R-free}$	-	6	73	76	20	18	0	69	-	61	73	16
P^*⟶B $_{R-work}$	8	6	37	61	4	8	4	0	100	18	55	6
P^*⟶B $_{R-free}$	-	12	41	61	8	12	24	0	-	45	55	14
S^* $_{R-work}$	0	0	0	0	0	0	0	0	0	0	0	0
S^* $_{R-free}$	-	-	-	-	-	-	-	-	-	-	-	-
S^*⟶A $_{R-work}$	27	16	94	92	29	41	20	80	100	0	96	35
S^*⟶A $_{R-free}$	-	4	51	55	8	8	18	51	-	0	53	12
S^*⟶B $_{R-work}$	2	0	22	37	0	2	2	18	100	2	0	0
S^*⟶B $_{R-free}$	-	6	29	45	0	2	20	22	-	43	0	2
S^*⟶P^* $_{R-work}$	29	10	92	96	16	22	25	86	100	37	94	0
S^*⟶P^* $_{R-free}$	-	22	94	94	22	22	65	82	-	78	94	0

0 — 100

Table 7. *Comparison of R-work/R-free (rounded to two decimal places) for the models generated from the 52 original HA-NCS data sets. Each row shows the percentage of models that a pipeline variant built with equal R-work or R-free to each other pipeline variant.*

Pipeline variant	A	A→P^*	A→B	B	B→P^*	P^*	P^*→A	P^*→B	S^*	S^*→A	S^*→B	S^*→P^*
A $_{R-work}$	100	18	4	2	20	14	25	6	0	24	4	24
A $_{R-free}$	-	-	-	-	-	-	-	-	-	-	-	-
A→P^* $_{R-work}$	18	100	0	2	22	16	18	2	0	14	0	14
A→P^* $_{R-free}$	-	100	2	2	14	16	14	6	-	6	2	16
A→B $_{R-work}$	4	0	100	25	12	8	0	37	2	2	25	4
A→B $_{R-free}$	-	2	100	24	6	2	4	35	-	4	18	2
B $_{R-work}$	2	2	25	100	2	2	2	27	0	6	35	2
B $_{R-free}$	-	2	24	100	2	4	10	14	-	2	18	0
B→P^* $_{R-work}$	20	22	12	2	100	37	16	6	0	14	4	27
B→P^* $_{R-free}$	-	14	6	2	100	22	12	6	-	16	6	25
P^* $_{R-work}$	14	16	8	2	37	100	16	4	0	14	2	47
P^* $_{R-free}$	-	16	2	4	22	100	16	4	-	10	0	43
P^*→A $_{R-work}$	25	18	0	2	16	16	100	2	0	29	0	16
P^*→A $_{R-free}$	-	14	4	10	12	16	100	8	-	22	8	20
P^*→B $_{R-work}$	6	2	37	27	6	4	2	100	0	2	27	8
P^*→B $_{R-free}$	-	6	35	14	6	4	8	100	-	4	24	4
S^* $_{R-work}$	0	0	2	0	0	0	0	0	100	0	0	0
S^* $_{R-free}$	-	-	-	-	-	-	-	-	-	-	-	-
S^*→A $_{R-work}$	24	14	2	6	14	14	29	2	0	100	2	27
S^*→A $_{R-free}$	-	6	4	2	16	10	22	4	-	100	4	10
S^*→B $_{R-work}$	4	0	25	35	4	2	0	27	0	2	100	6
S^*→B $_{R-free}$	-	2	18	18	6	0	8	24	-	4	100	4
S^*→P^* $_{R-work}$	24	14	4	2	27	47	16	8	0	27	6	100
S^*→P^* $_{R-free}$	-	16	2	0	25	43	20	4	-	10	4	100

0 100

Table 8. *Comparison of R-work/R-free (rounded to two decimal places) for the models generated from the 52 original HA-NCS data sets. Each row shows the percentage of models that a pipeline variant built with R-work or R-free at least 5% lower than each other pipeline variant.*

Pipeline variant	A	A→P^*	A→B	B	B→P^*	P^*	P^*→A	P^*→B	S^*	S^*→A	S^*→B	S^*→P^*
A $_{R-work}$	0	14	53	59	10	12	4	41	100	22	55	14
A $_{R-free}$	-	-	-	-	-	-	-	-	-	-	-	-
A→P^* $_{R-work}$	2	0	57	67	0	6	4	49	100	16	71	4
A→P^* $_{R-free}$	-	0	45	61	0	4	20	39	-	49	59	0
A→B $_{R-work}$	0	0	0	12	0	2	0	10	96	2	6	0
A→B $_{R-free}$	-	2	0	10	0	2	8	8	-	33	4	0
B $_{R-work}$	0	0	6	0	0	0	0	6	96	2	4	0
B $_{R-free}$	-	2	8	0	0	0	6	4	-	31	4	0
B→P^* $_{R-work}$	4	4	51	61	0	4	6	45	100	16	59	2
B→P^* $_{R-free}$	-	6	53	63	0	2	22	41	-	39	59	2
P^* $_{R-work}$	4	4	39	49	0	0	2	22	100	12	49	0
P^* $_{R-free}$	-	6	39	51	0	0	16	27	-	43	49	2
P^*→A $_{R-work}$	4	12	47	61	12	14	0	43	100	18	53	14
P^*→A $_{R-free}$	-	4	18	31	0	0	0	18	-	31	29	0
P^*→B $_{R-work}$	2	2	16	22	0	0	0	0	100	6	20	0
P^*→B $_{R-free}$	-	6	14	22	0	0	10	0	-	37	18	0
S^* $_{R-work}$	0	0	0	0	0	0	0	0	0	0	0	0
S^* $_{R-free}$	-	-	-	-	-	-	-	-	-	-	-	-
S^*→A $_{R-work}$	2	0	35	51	0	4	2	29	100	0	49	4
S^*→A $_{R-free}$	-	0	12	20	0	0	0	10	-	0	16	0
S^*→B $_{R-work}$	0	0	6	4	0	2	0	6	96	2	0	0
S^*→B $_{R-free}$	-	0	4	6	0	2	8	6	-	29	0	0
S^*→P^* $_{R-work}$	4	4	41	53	0	2	2	25	100	12	49	0
S^*→P^* $_{R-free}$	-	8	37	53	0	4	14	25	-	39	43	0

0 100

Table 9. *Comparison of R-work/R-free (rounded to two decimal places) for the models generated from the 52 original HA-NCS data sets. Each row shows the percentage of models that a pipeline variant built with R-work or R-free between 1% and 4% lower than each other pipeline variant.*

Pipeline variant	A	A→P^*	A→B	B	B→P^*	P^*	P^*→A	P^*→B	S^*	S^*→A	S^*→B	S^*→P^*
A $_{R-work}$	0	12	39	37	29	39	27	45	0	27	39	33
A $_{R-free}$	-	-	-	-	-	-	-	-	-	-	-	-
A→P^* $_{R-work}$	55	0	41	31	51	67	47	43	0	55	29	73
A→P^* $_{R-free}$	-	0	41	29	43	57	61	43	-	41	33	63
A→B $_{R-work}$	4	2	0	41	2	4	4	16	2	2	47	4
A→B $_{R-free}$	-	10	0	37	4	4	16	16	-	12	49	4
B $_{R-work}$	2	0	16	0	0	0	2	6	4	0	24	2
B $_{R-free}$	-	6	22	0	2	6	8	22	-	12	33	6
B→P^* $_{R-work}$	37	24	35	37	0	41	33	45	0	41	37	55
B→P^* $_{R-free}$	-	37	37	33	0	45	47	45	-	37	35	51
P^* $_{R-work}$	31	8	47	49	18	0	25	67	0	33	47	31
P^* $_{R-free}$	-	18	53	39	31	0	51	57	-	39	49	33
P^*→A $_{R-work}$	39	20	49	35	33	43	0	51	0	33	45	45
P^*→A $_{R-free}$	-	2	55	45	20	18	0	51	-	29	43	16
P^*→B $_{R-work}$	6	4	22	39	4	8	4	0	0	12	35	6
P^*→B $_{R-free}$	-	6	27	39	8	12	14	0	-	8	37	14
S^* $_{R-work}$	0	0	0	0	0	0	0	0	0	0	0	0
S^* $_{R-free}$	-	-	-	-	-	-	-	-	-	-	-	-
S^*→A $_{R-work}$	25	16	59	41	29	37	18	51	0	0	47	31
S^*→A $_{R-free}$	-	4	39	35	8	8	18	41	-	0	37	12
S^*→B $_{R-work}$	2	0	16	33	0	0	2	12	4	0	0	0
S^*→B $_{R-free}$	-	6	25	39	0	0	12	16	-	14	0	2
S^*→P^* $_{R-work}$	25	6	51	43	16	20	24	61	0	25	45	0
S^*→P^* $_{R-free}$	-	14	57	41	22	18	51	57	-	39	51	0

0 73

Table 10. *Structure completeness comparison for the models generated from the 52 original MR-NCS data sets. Each row corresponds to a pipeline variant, and shows the percentage (rounded to the nearest integer) of models that the pipeline variant built with higher structure completeness than each of the other pipeline variants.*

Pipeline variant	A	A$\rightarrow P^*$	A$\rightarrow B$	B	B$\rightarrow P^*$	P^*	$P^*\longrightarrow A$	$P^*\longrightarrow B$	S^*	$S^*\longrightarrow A$	$S^*\longrightarrow B$	$S^*\longrightarrow P^*$
A	0	25	22	25	22	37	27	14	73	51	29	39
A$\rightarrow P^*$	51	0	31	41	24	37	49	16	94	67	43	45
A$\rightarrow B$	65	61	0	45	37	65	75	31	94	78	57	61
B	59	47	29	0	25	49	55	20	94	73	41	61
B$\rightarrow P^*$	61	63	43	61	0	67	65	35	92	78	55	80
P^*	49	37	29	37	25	0	47	16	86	59	39	47
$P^*\longrightarrow A$	43	24	18	25	22	35	0	8	80	55	31	43
$P^*\longrightarrow B$	78	65	53	65	51	69	82	0	96	82	67	80
S^*	22	2	6	6	4	8	14	4	0	39	4	8
$S^*\longrightarrow A$	20	18	16	20	12	31	20	10	57	0	14	31
$S^*\longrightarrow B$	59	43	25	39	33	53	49	16	96	71	0	51
$S^*\longrightarrow P^*$	47	39	29	35	12	31	45	14	88	59	37	0

0 96

Table 11. *Structure completeness comparison for the models generated from the 52 original MR-NCS data sets. Each row corresponds to a pipeline variant, and shows the percentage (rounded to the nearest integer) of models that the pipeline variant built with equal structure completeness to each of the other pipeline variants.*

Pipeline variant	A	A$\rightarrow P^*$	A$\rightarrow B$	B	B$\rightarrow P^*$	P^*	$P^*\longrightarrow A$	$P^*\longrightarrow B$	S^*	$S^*\longrightarrow A$	$S^*\longrightarrow B$	$S^*\longrightarrow P^*$
A	100	24	14	16	18	14	29	8	6	29	12	14
A$\rightarrow P^*$	24	100	8	12	14	25	27	20	4	16	14	16
A$\rightarrow B$	14	8	100	25	20	6	8	16	0	6	18	10
B	16	12	25	100	14	14	20	16	0	8	20	4
B$\rightarrow P^*$	18	14	20	14	100	8	14	14	4	10	12	8
P^*	14	25	6	14	8	100	18	16	6	10	8	22
$P^*\longrightarrow A$	29	27	8	20	14	18	100	10	6	25	20	12
$P^*\longrightarrow B$	8	20	16	16	14	16	10	100	0	8	18	6
S^*	6	4	0	0	4	6	6	0	100	4	0	4
$S^*\longrightarrow A$	29	16	6	8	10	10	25	8	4	100	16	10
$S^*\longrightarrow B$	12	14	18	20	12	8	20	18	0	16	100	12
$S^*\longrightarrow P^*$	14	16	10	4	8	22	12	6	4	10	12	100

0 100

Table 12. *Structure completeness comparison for the models generated from the 52 original MR-NCS data sets. Each row corresponds to a pipeline variant, and shows the percentage (rounded to the nearest integer) of models that the pipeline variant built with at least 5% higher structure completeness than each of the other pipeline variants.*

Pipeline variant	A	A→P^*	A→B	B	B→P^*	P^*	P^*→A	P^*→B	S^*	S^*→A	S^*→B	S^*→P^*
A	0	6	8	10	10	10	8	8	61	35	10	14
A→P^*	31	0	16	18	12	10	27	10	82	49	24	18
A→B	35	22	0	14	12	31	31	8	75	47	18	35
B	35	20	16	0	10	22	33	10	76	45	18	27
B→P^*	43	29	24	25	0	31	35	10	78	49	25	43
P^*	31	18	16	20	10	0	25	12	76	43	22	20
P^*→A	16	8	10	16	10	10	0	4	65	39	12	20
P^*→B	35	24	18	25	18	29	35	0	82	49	31	39
S^*	10	0	2	2	2	4	8	4	0	31	0	6
S^*→A	6	0	4	6	6	8	6	6	41	0	6	12
S^*→B	37	18	8	12	8	25	27	8	78	45	0	27
S^*→P^*	31	12	20	20	10	12	24	12	75	45	20	0

0 82

Table 13. *Structure completeness comparison for the models generated from the 52 original MR-NCS data sets. Each row corresponds to a pipeline variant, and shows the percentage (rounded to the nearest integer) of models that the pipeline variant built with between 1% and 4% higher structure completeness than each of the other pipeline variants.*

Pipeline variant	A	A→P^*	A→B	B	B→P^*	P^*	P^*→A	P^*→B	S^*	S^*→A	S^*→B	S^*→P^*
A	0	20	14	16	12	27	20	6	12	16	20	25
A→P^*	20	0	16	24	12	27	22	6	12	18	20	27
A→B	29	39	0	31	25	33	43	24	20	31	39	25
B	24	27	14	0	16	27	22	10	18	27	24	33
B→P^*	18	33	20	35	0	35	29	25	14	29	29	37
P^*	18	20	14	18	16	0	22	4	10	16	18	27
P^*→A	27	16	8	10	12	25	0	4	16	16	20	24
P^*→B	43	41	35	39	33	39	47	0	14	33	35	41
S^*	12	2	4	4	2	4	6	0	0	8	4	2
S^*→A	14	18	12	14	6	24	14	4	16	0	8	20
S^*→B	22	25	18	27	25	27	22	8	18	25	0	24
S^*→P^*	16	27	10	16	2	20	22	2	14	14	18	0

0 47

Table 14. *Comparison of R-work/R-free (rounded to two decimal places) for the models generated from the 52 original MR-NCS data sets. Each row shows the percentage of models that a pipeline variant built with lower R-work or R-free than each other pipeline variant.*

Pipeline variant	A	A→P^*	A→B	B	B→P^*	P^*	P^*⟶A	P^*⟶B	S^*	S^*⟶A	S^*⟶B	S^*⟶P^*
A $_{R-work}$	0	25	88	92	39	57	22	84	100	43	90	45
A $_{R-free}$	-	-	-	-	-	-	-	-	-	-	-	-
A→P^* $_{R-work}$	59	0	98	96	55	78	47	92	100	69	100	75
A→P^* $_{R-free}$	-	0	92	92	49	61	82	86	-	90	96	63
A→B $_{R-work}$	6	0	0	39	4	4	0	27	98	8	49	4
A→B $_{R-free}$	-	4	0	37	6	4	18	24	-	43	49	8
B $_{R-work}$	4	0	25	0	2	2	0	18	100	4	33	6
B $_{R-free}$	-	8	33	0	6	6	18	25	-	49	43	8
B→P^* $_{R-work}$	49	31	84	96	0	51	39	88	100	55	92	63
B→P^* $_{R-free}$	-	33	88	92	0	51	69	88	-	78	90	63
P^* $_{R-work}$	27	10	86	92	20	0	20	88	100	43	96	29
P^* $_{R-free}$	-	16	88	86	24	0	65	86	-	80	98	39
P^*⟶A $_{R-work}$	51	39	98	100	43	69	0	96	100	47	100	65
P^*⟶A $_{R-free}$	-	6	71	71	24	16	0	71	-	61	75	22
P^*⟶B $_{R-work}$	8	6	39	53	6	6	2	0	100	14	55	6
P^*⟶B $_{R-free}$	-	6	43	55	8	8	24	0	-	45	57	12
S^* $_{R-work}$	0	0	0	0	0	0	0	0	0	0	0	0
S^* $_{R-free}$	-	-	-	-	-	-	-	-	-	-	-	-
S^*⟶A $_{R-work}$	35	16	90	92	24	39	12	84	100	0	94	39
S^*⟶A $_{R-free}$	-	2	49	49	10	8	16	49	-	0	51	14
S^*⟶B $_{R-work}$	4	0	14	27	2	0	0	18	100	4	0	0
S^*⟶B $_{R-free}$	-	2	25	31	2	0	18	14	-	45	0	4
S^*⟶P^* $_{R-work}$	31	10	88	94	16	27	24	88	100	41	92	0
S^*⟶P^* $_{R-free}$	-	18	92	92	16	27	63	80	-	78	94	0

0 100

Table 15. *Comparison of R-work/R-free (rounded to two decimal places) for the models generated from the 52 original MR-NCS data sets. Each row shows the percentage of models that a pipeline variant built with equal R-work or R-free to each other pipeline variant.*

Pipeline variant	A	A→P^*	A→B	B	B→P^*	P^*	$P^*\longrightarrow A$	$P^*\longrightarrow B$	S^*	$S^*\longrightarrow A$	$S^*\longrightarrow B$	$S^*\longrightarrow P^*$
A $_{R-work}$	100	16	6	4	12	16	27	8	0	22	6	24
A $_{R-free}$	-	-	-	-	-	-	-	-	-	-	-	-
A→P^* $_{R-work}$	16	100	2	4	14	12	14	2	0	16	0	16
A→P^* $_{R-free}$	-	100	4	0	18	24	12	8	-	8	2	20
A→B $_{R-work}$	6	2	100	35	12	10	2	33	2	2	37	8
A→B $_{R-free}$	-	4	100	29	6	8	12	33	-	8	25	0
B $_{R-work}$	4	4	35	100	2	6	0	29	0	4	39	0
B $_{R-free}$	-	0	29	100	2	8	12	20	-	2	25	0
B→P^* $_{R-work}$	12	14	12	2	100	29	18	6	0	22	6	22
B→P^* $_{R-free}$	-	18	6	2	100	25	8	4	-	12	8	22
P^* $_{R-work}$	16	12	10	6	29	100	12	6	0	18	4	43
P^* $_{R-free}$	-	24	8	8	25	100	20	6	-	12	2	33
$P^*\longrightarrow A$ $_{R-work}$	27	14	2	0	18	12	100	2	0	41	0	12
$P^*\longrightarrow A$ $_{R-free}$	-	12	12	12	8	20	100	6	-	24	8	16
$P^*\longrightarrow B$ $_{R-work}$	8	2	33	29	6	6	2	100	0	2	27	6
$P^*\longrightarrow B$ $_{R-free}$	-	8	33	20	4	6	6	100	-	6	29	8
S^* $_{R-work}$	0	0	2	0	0	0	0	0	100	0	0	0
S^* $_{R-free}$	-	-	-	-	-	-	-	-	-	-	-	-
$S^*\longrightarrow A$ $_{R-work}$	22	16	2	4	22	18	41	2	0	100	2	20
$S^*\longrightarrow A$ $_{R-free}$	-	8	8	2	12	12	24	6	-	100	4	8
$S^*\longrightarrow B$ $_{R-work}$	6	0	37	39	6	4	0	27	0	2	100	8
$S^*\longrightarrow B$ $_{R-free}$	-	2	25	25	8	2	8	29	-	4	100	2
$S^*\longrightarrow P^*$ $_{R-work}$	24	16	8	0	22	43	12	6	0	20	8	100
$S^*\longrightarrow P^*$ $_{R-free}$	-	20	0	0	22	33	16	8	-	8	2	100

0 100

Table 16. *Comparison of R-work/R-free (rounded to two decimal places) for the models generated from the 52 original MR-NCS data sets. Each row shows the percentage of models that a pipeline variant built with R-work or R-free at least 5% lower than each other pipeline variant.*

Pipeline variant	A	A→P^*	A→B	B	B→P^*	P^*	P^*→A	P^*→B	S^*	S^*→A	S^*→B	S^*→P^*
A $_{R-work}$	0	6	47	53	8	10	0	43	100	18	55	8
A $_{R-free}$	-	-	-	-	-	-	-	-	-	-	-	-
A→P^* $_{R-work}$	0	0	61	61	4	4	0	55	100	12	69	4
A→P^* $_{R-free}$	-	0	51	55	2	4	18	39	-	45	55	0
A→B $_{R-work}$	0	0	0	10	0	0	0	8	96	2	6	0
A→B $_{R-free}$	-	2	0	4	2	0	4	8	-	29	4	0
B $_{R-work}$	0	0	4	0	0	0	0	8	98	2	6	0
B $_{R-free}$	-	2	8	0	2	0	4	6	-	31	10	0
B→P^* $_{R-work}$	4	6	49	53	0	4	2	47	100	18	57	2
B→P^* $_{R-free}$	-	10	51	45	0	10	24	41	-	45	57	2
P^* $_{R-work}$	2	4	39	39	2	0	0	27	100	10	49	0
P^* $_{R-free}$	-	6	35	41	2	0	10	31	-	39	49	2
P^*→A $_{R-work}$	2	10	45	53	14	14	0	51	100	22	53	14
P^*→A $_{R-free}$	-	4	24	24	2	0	0	16	-	29	33	0
P^*→B $_{R-work}$	0	2	16	18	2	0	0	0	98	6	16	0
P^*→B $_{R-free}$	-	4	18	16	2	0	8	0	-	35	16	0
S^* $_{R-work}$	0	0	0	0	0	0	0	0	0	0	0	0
S^* $_{R-free}$	-	-	-	-	-	-	-	-	-	-	-	-
S^*→A $_{R-work}$	0	0	37	41	4	0	0	33	100	0	49	4
S^*→A $_{R-free}$	-	0	12	12	0	0	0	8	-	0	18	0
S^*→B $_{R-work}$	0	0	4	6	0	0	0	4	98	2	0	0
S^*→B $_{R-free}$	-	0	4	4	2	0	4	4	-	27	0	0
S^*→P^* $_{R-work}$	2	4	39	37	2	0	0	27	100	12	49	0
S^*→P^* $_{R-free}$	-	4	37	43	2	0	12	29	-	39	51	0

0 100

Table 17. *Comparison of R-work/R-free (rounded to two decimal places) for the models generated from the 52 original MR-NCS data sets. Each row shows the percentage of models that a pipeline variant built with R-work or R-free between 1% and 4% lower than each other pipeline variant.*

Pipeline variant	A	A→P^*	A→B	B	B→P^*	P^*	P^*→A	P^*→B	S^*	S^*→A	S^*→B	S^*→P^*
A $_{R-work}$	0	20	41	39	31	47	22	41	0	25	35	37
A $_{R-free}$	-	-	-	-	-	-	-	-	-	-	-	-
A→P^* $_{R-work}$	59	0	37	35	51	75	47	37	0	57	31	71
A→P^* $_{R-free}$	-	0	41	37	47	57	65	47	-	45	41	63
A→B $_{R-work}$	6	0	0	29	4	4	0	20	2	6	43	4
A→B $_{R-free}$	-	2	0	33	4	4	14	16	-	14	45	8
B $_{R-work}$	4	0	22	0	2	2	0	10	2	2	27	6
B $_{R-free}$	-	6	25	0	4	6	14	20	-	18	33	8
B→P^* $_{R-work}$	45	25	35	43	0	47	37	41	0	37	35	61
B→P^* $_{R-free}$	-	24	37	47	0	41	45	47	-	33	33	61
P^* $_{R-work}$	25	6	47	53	18	0	20	61	0	33	47	29
P^* $_{R-free}$	-	10	53	45	22	0	55	55	-	41	49	37
P^*→A $_{R-work}$	49	29	53	47	29	55	0	45	0	25	47	51
P^*→A $_{R-free}$	-	2	47	47	22	16	0	55	-	31	41	22
P^*→B $_{R-work}$	8	4	24	35	4	6	2	0	2	8	39	6
P^*→B $_{R-free}$	-	2	25	39	6	8	16	0	-	10	41	12
S^* $_{R-work}$	0	0	0	0	0	0	0	0	0	0	0	0
S^* $_{R-free}$	-	-	-	-	-	-	-	-	-	-	-	-
S^*→A $_{R-work}$	35	16	53	51	20	39	12	51	0	0	45	35
S^*→A $_{R-free}$	-	2	37	37	10	8	16	41	-	0	33	14
S^*→B $_{R-work}$	4	0	10	22	2	0	0	14	2	2	0	0
S^*→B $_{R-free}$	-	2	22	27	0	0	14	10	-	18	0	4
S^*→P^* $_{R-work}$	29	6	49	57	14	27	24	61	0	29	43	0
S^*→P^* $_{R-free}$	-	14	55	49	14	27	51	51	-	39	43	0

0 75

Table 18. *Structure completeness comparison for the models generated from the 52 original NO-NCS data sets. Each row corresponds to a pipeline variant, and shows the percentage (rounded to the nearest integer) of models that the pipeline variant built with higher structure completeness than each of the other pipeline variants.*

Pipeline variant	A	A$\rightarrow P^*$	A$\rightarrow P$	A$\rightarrow B$	B	B$\rightarrow P^*$	B$\rightarrow P$	P^*	$P^*\longrightarrow A$	$P^*\longrightarrow B$	P	P$\rightarrow A$	P$\rightarrow B$	S	S$\rightarrow A$	S$\rightarrow B$	S^*	$S^*\longrightarrow A$	$S^*\longrightarrow B$	$S^*\longrightarrow P^*$	$S^*\longrightarrow P$	S$\longrightarrow P^*$	S$\rightarrow P$
A	0	22	22	16	27	31	27	35	31	22	41	37	14	76	71	35	75	51	29	41	37	41	41
A$\rightarrow P^*$	63	0	25	35	55	45	41	43	63	20	49	67	24	92	82	59	94	67	53	59	55	51	53
A$\rightarrow P$	59	25	0	27	51	49	39	39	55	20	43	59	22	88	80	57	90	69	51	57	49	59	55
A$\rightarrow B$	67	55	55	0	55	45	39	61	61	24	57	61	27	84	82	49	86	76	59	59	55	61	57
B	61	31	37	31	0	25	25	39	53	22	45	53	10	88	80	41	90	73	45	49	49	47	47
B$\rightarrow P^*$	53	37	43	41	57	0	24	53	55	31	47	53	22	88	73	57	90	69	53	57	55	57	61
B$\rightarrow P$	53	45	47	41	55	25	0	59	57	27	53	57	24	88	73	53	90	67	51	57	59	63	65
P^*	53	29	33	29	51	37	31	0	49	18	39	45	14	78	63	53	84	59	49	43	45	45	37
$P^*\longrightarrow A$	45	12	20	24	35	31	27	31	0	20	41	33	10	80	69	41	84	55	31	45	49	47	43
$P^*\longrightarrow B$	73	61	63	61	67	57	61	71	73	0	65	75	37	88	82	65	94	75	69	75	73	76	75
P	49	25	31	31	41	37	31	27	47	18	0	43	10	80	65	47	84	61	45	35	37	37	37
P$\rightarrow A$	43	22	27	27	33	31	25	37	41	18	39	0	6	84	65	43	86	59	31	45	41	43	43
P$\rightarrow B$	80	63	65	57	75	61	57	71	75	39	78	76	0	96	88	65	98	86	69	78	82	76	82
S	20	6	8	14	6	8	10	10	18	6	12	14	4	0	51	14	37	47	14	8	12	10	18
S$\rightarrow A$	8	8	10	14	16	18	16	29	12	12	25	14	6	45	0	18	49	20	8	24	33	33	31
S$\rightarrow B$	59	29	29	35	45	29	29	37	49	24	45	49	10	86	73	0	86	69	43	43	47	39	51
S^*	22	0	2	12	6	6	10	8	12	6	10	12	2	53	49	12	0	43	12	6	14	12	14
$S^*\longrightarrow A$	22	16	16	14	22	22	20	31	22	18	31	22	6	51	49	24	53	0	18	33	31	33	39
$S^*\longrightarrow B$	57	33	33	27	45	41	37	45	49	22	49	55	22	82	76	47	88	69	0	43	41	43	45
$S^*\longrightarrow P^*$	47	31	27	31	41	35	31	35	41	24	51	47	16	88	67	47	86	59	49	0	35	39	45
$S^*\longrightarrow P$	45	31	35	29	43	27	24	41	41	18	43	47	12	82	59	47	80	55	49	45	0	51	45
S$\longrightarrow P^*$	49	25	24	31	41	35	27	35	43	14	37	51	20	82	63	53	82	57	49	27	33	0	33
S$\rightarrow P$	49	24	31	33	41	27	24	33	45	16	39	45	14	76	57	43	76	53	47	39	37	41	0

0 98

Table 19. *Structure completeness comparison for the models generated from the 52 original NO-NCS data sets. Each row corresponds to a pipeline variant, and shows the percentage (rounded to the nearest integer) of models that the pipeline variant built with equal structure completeness to each of the other pipeline variants.*

Pipeline variant	A	A→P^*	A→P	A→B	B	B→P^*	B→P	P^*	P^*→A	P^*→B	P	P→A	P→B	S	S→A	S→B	S^*	S^*→A	S^*→B	S^*→P^*	S^*→P	S→P^*	S→P
A	100	16	20	18	12	16	20	12	24	6	10	20	6	4	22	6	4	27	14	12	18	10	10
A→P^*	16	100	49	10	14	18	14	27	25	20	25	12	14	2	10	12	6	18	14	10	14	24	24
A→P	20	49	100	18	12	8	14	27	25	18	25	14	14	4	10	14	8	16	16	16	16	18	14
A→B	18	10	18	100	14	14	20	10	16	16	12	12	16	2	4	16	2	10	14	10	16	8	10
B	12	14	12	14	100	18	20	10	12	12	14	14	16	6	4	14	4	6	10	10	8	12	12
B→P^*	16	18	8	14	18	100	51	10	14	12	16	16	18	4	10	14	4	10	6	8	18	8	12
B→P	20	14	14	20	20	51	100	10	16	12	16	18	20	2	12	18	0	14	12	12	18	10	12
P^*	12	27	27	10	10	10	10	100	20	12	33	18	16	12	8	10	8	10	6	22	14	20	29
P^*→A	24	25	25	16	12	14	16	20	100	8	12	25	16	2	20	10	4	24	20	14	10	10	12
P^*→B	6	20	18	16	12	12	12	12	8	100	18	8	24	6	6	12	0	8	10	2	10	10	10
P	10	25	25	12	14	16	16	33	12	18	100	18	12	8	10	8	6	8	6	14	20	25	24
P→A	20	12	14	12	14	16	18	18	25	8	18	100	18	2	22	8	2	20	14	8	12	6	12
P→B	6	14	14	16	16	18	20	16	16	24	12	18	100	0	6	25	0	8	10	6	6	4	4
S	4	2	4	2	6	4	2	12	2	6	8	2	0	100	4	0	10	2	4	4	6	8	6
S→A	22	10	10	4	4	10	12	8	20	6	10	22	6	4	100	10	2	31	16	10	8	4	12
S→B	6	12	14	16	14	14	18	10	10	12	8	8	25	0	10	100	2	8	10	10	6	8	6
S^*	4	6	8	2	4	4	0	8	4	0	6	2	0	10	2	2	100	4	0	8	6	6	10
S^*→A	27	18	16	10	6	10	14	10	24	8	8	20	8	2	31	8	4	100	14	8	14	10	8
S^*→B	14	14	16	14	10	6	12	6	20	10	6	14	10	4	16	10	0	14	100	8	10	8	8
S^*→P^*	12	10	16	10	10	8	12	22	14	2	14	8	6	4	10	10	8	8	8	100	20	33	16
S^*→P	18	14	16	16	8	18	18	14	10	10	20	12	6	6	8	6	6	14	10	20	100	16	18
S→P^*	10	24	18	8	12	8	10	20	10	10	25	6	4	8	4	8	6	10	8	33	16	100	25
S→P	10	24	14	10	12	12	12	29	12	10	24	12	4	6	12	6	10	8	8	16	18	25	100

0 100

Table 20. *Structure completeness comparison for the models generated from the 52 original NO-NCS data sets. Each row corresponds to a pipeline variant, and shows the percentage (rounded to the nearest integer) of models that the pipeline variant built with at least 5% higher structure completeness than each of the other pipeline variants.*

Pipeline variant	A	A$\rightarrow P^*$	A$\rightarrow P$	A$\rightarrow B$	B	B$\rightarrow P^*$	B$\rightarrow P$	P^*	$P^*\longrightarrow A$	$P^*\longrightarrow B$	P	P$\rightarrow A$	P$\rightarrow B$	S	S$\rightarrow A$	S$\rightarrow B$	S^*	$S^*\longrightarrow A$	$S^*\longrightarrow B$	$S^*\longrightarrow P^*$	$S^*\longrightarrow P$	S$\longrightarrow P^*$	S$\rightarrow P$
A	0	0	0	10	14	14	12	12	8	16	14	8	4	57	43	14	61	37	10	16	18	16	16
A$\rightarrow P^*$	33	0	8	20	27	20	20	12	29	16	22	25	8	73	57	29	80	53	22	18	25	20	24
A$\rightarrow P$	31	10	0	24	27	24	18	16	27	16	18	27	6	69	57	29	76	49	25	22	24	18	20
A$\rightarrow B$	33	12	10	0	20	22	18	25	20	8	29	27	2	63	47	27	69	47	24	24	29	31	27
B	31	12	10	14	0	18	14	22	25	12	27	20	4	67	53	16	78	43	14	16	18	24	24
B$\rightarrow P^*$	37	16	22	25	29	0	6	22	27	12	27	27	10	67	57	31	76	51	27	31	33	29	31
B$\rightarrow P$	33	20	24	25	33	2	0	27	27	16	31	29	8	67	57	31	75	49	27	33	31	31	33
P^*	33	16	16	24	27	18	16	0	27	10	14	31	8	67	53	33	71	45	27	16	16	12	14
$P^*\longrightarrow A$	24	4	4	16	18	16	16	14	0	12	18	12	6	59	47	25	69	45	14	20	20	16	20
$P^*\longrightarrow B$	37	27	24	25	31	22	24	35	33	0	37	31	14	67	55	41	73	49	33	31	41	39	43
P	33	12	12	24	31	18	20	8	27	12	0	24	6	67	53	31	73	49	25	14	20	14	22
P$\rightarrow A$	22	6	4	20	24	12	16	10	12	12	16	0	6	61	51	25	69	47	20	20	20	16	20
P$\rightarrow B$	37	25	24	29	33	29	29	33	27	18	39	35	0	73	63	41	80	55	37	35	41	35	41
S	12	2	2	6	0	2	2	6	6	4	8	2	2	0	37	6	10	35	2	6	8	4	4
S$\rightarrow A$	2	0	0	8	6	8	10	8	4	12	6	2	2	24	0	6	33	12	2	8	8	12	14
S$\rightarrow B$	29	12	12	18	12	16	10	20	20	12	24	22	4	67	51	0	76	49	14	20	22	24	27
S^*	12	0	0	6	0	4	4	4	8	4	8	4	2	20	41	6	0	37	2	4	4	2	0
$S^*\longrightarrow A$	6	0	0	6	4	10	10	10	4	10	16	2	2	35	25	8	39	0	4	12	12	14	10
$S^*\longrightarrow B$	35	10	8	16	20	18	16	20	22	18	25	24	4	65	55	18	75	47	0	16	18	20	25
$S^*\longrightarrow P^*$	33	16	18	25	27	25	24	16	29	16	16	27	8	67	53	27	73	49	24	0	14	8	20
$S^*\longrightarrow P$	33	14	16	22	24	20	16	8	25	12	12	29	10	63	47	25	73	47	22	8	0	10	14
S$\longrightarrow P^*$	35	18	14	24	29	18	16	6	25	10	12	25	8	61	51	33	71	47	25	12	12	0	14
S$\rightarrow P$	31	12	14	20	22	14	14	10	25	10	14	29	6	65	47	31	69	43	24	12	12	10	0

0 … 80

Table 21. *Structure completeness comparison for the models generated from the 52 original NO-NCS data sets. Each row corresponds to a pipeline variant, and shows the percentage (rounded to the nearest integer) of models that the pipeline variant built with between 1% and 4% higher structure completeness than each of the other pipeline variants.*

Pipeline variant	A	A→P^*	A→P	A→B	B	B→P^*	B→P	P^*	P^*→A	P^*→B	P	P→A	P→B	S	S→A	S→B	S^*	S^*→A	S^*→B	S^*→P^*	S^*→P	S→P^*	S→P
A	0	22	22	6	14	18	16	24	24	6	27	29	10	20	27	22	14	14	20	25	20	25	25
A→P^*	29	0	18	16	27	25	22	31	33	4	27	41	16	20	25	29	14	14	31	41	29	31	29
A→P	27	16	0	4	24	25	22	24	27	4	25	31	16	20	24	27	14	20	25	35	25	41	35
A→B	33	43	45	0	35	24	22	35	41	16	27	33	25	22	35	22	18	29	35	35	25	29	29
B	29	20	27	18	0	8	12	18	27	10	18	33	6	22	27	25	12	29	31	33	31	24	24
B→P^*	16	22	22	16	27	0	18	31	27	20	20	25	12	22	16	25	14	18	25	25	22	27	29
B→P	20	25	24	16	22	24	0	31	29	12	22	27	16	22	16	22	16	18	24	24	27	31	31
P^*	20	14	18	6	24	20	16	0	22	8	25	14	6	12	10	20	14	14	22	27	29	33	24
P^*→A	22	8	16	8	18	16	12	18	0	8	24	22	4	22	22	16	16	10	18	25	29	31	24
P^*→B	35	33	39	35	35	35	37	35	39	0	27	43	24	22	27	24	22	25	35	43	31	37	31
P	16	14	20	8	10	20	12	20	20	6	0	20	4	14	12	16	12	12	20	22	18	24	16
P→A	22	16	24	8	10	20	10	27	29	6	24	0	0	24	14	18	18	12	12	25	22	27	24
P→B	43	37	41	27	41	31	27	37	47	22	39	41	0	24	25	24	18	31	31	43	41	41	41
S	8	4	6	8	6	6	8	4	12	2	4	12	2	0	14	8	27	12	12	2	4	6	14
S→A	6	8	10	6	10	10	6	22	8	0	20	12	4	22	0	12	16	8	6	16	25	22	18
S→B	29	18	18	18	33	14	20	18	29	12	22	27	6	20	22	0	10	20	29	24	25	16	24
S^*	10	0	2	6	6	2	6	4	4	2	2	8	0	33	8	6	0	6	10	2	10	10	14
S^*→A	16	16	16	8	18	12	10	22	18	8	16	20	4	16	24	16	14	0	14	22	20	20	29
S^*→B	22	24	25	12	25	24	22	25	27	4	24	31	18	18	22	29	14	22	0	27	24	24	20
S^*→P^*	14	16	10	6	14	10	8	20	12	8	35	20	8	22	14	20	14	10	25	0	22	31	25
S^*→P	12	18	20	8	20	8	8	33	16	6	31	18	2	20	12	22	8	8	27	37	0	41	31
S→P^*	14	8	10	8	12	18	12	29	18	4	25	25	12	22	12	20	12	10	24	16	22	0	20
S→P	18	12	18	14	20	14	10	24	20	6	25	16	8	12	10	12	8	10	24	27	25	31	0

0 47

Table 22. *Comparison of R-work/R-free (rounded to two decimal places) for the models generated from the 52 original NO-NCS data sets. Each row shows the percentage of models that a pipeline variant built with lower R-work or R-free than each other pipeline variant.*

Pipeline variant	A	A→P^*	A→P	A→B	B	B→P^*	B→P	P^*	P^*→A	P^*→B	P	P→A	P→B	S	S→A	S→B	S^*	S^*→A	S^*→B	S^*→P^*	S^*→P	S→P^*	S→P
A $_{R-work}$	0	27	29	94	98	47	47	55	22	84	49	22	94	100	51	98	100	41	96	47	51	51	45
A $_{R-free}$	-	-	-	-	-	-	-	-	-	-	-	-	-	-	-	-	-	-	-	-	-	-	-
A→P^* $_{R-work}$	59	0	18	100	100	61	59	71	45	94	69	49	98	100	75	100	100	69	100	73	71	65	71
A→P^* $_{R-free}$	-	0	29	94	96	51	47	53	84	82	51	82	86	-	90	98	-	90	98	55	57	51	53
A→P $_{R-work}$	57	24	0	100	100	67	61	69	47	94	73	49	98	100	78	100	100	71	100	73	73	73	71
A→P $_{R-free}$	-	27	0	92	98	51	45	51	84	82	59	84	86	-	92	96	-	94	98	57	51	51	57
A→B $_{R-work}$	2	0	0	0	49	6	6	2	2	20	4	0	16	98	16	55	98	12	53	2	4	4	4
A→B $_{R-free}$	-	2	0	0	51	8	6	2	14	25	4	14	16	-	43	53	-	49	51	4	2	2	4
B $_{R-work}$	0	0	0	18	0	0	0	0	0	20	0	0	6	100	10	41	100	4	33	2	2	2	2
B $_{R-free}$	-	2	0	27	0	2	0	2	10	31	4	10	8	-	45	43	-	47	41	2	4	2	2
B→P^* $_{R-work}$	37	25	20	84	98	0	16	41	27	86	51	33	84	100	61	96	100	53	96	47	45	47	51
B→P^* $_{R-free}$	-	35	37	88	96	0	18	31	65	86	45	71	84	-	78	96	-	73	94	43	39	43	39
B→P $_{R-work}$	35	22	12	84	100	18	0	41	29	84	45	33	90	100	59	94	100	53	96	49	39	47	47
B→P $_{R-free}$	-	31	37	92	94	29	0	41	69	82	49	65	90	-	78	96	-	80	96	51	41	47	49
P^* $_{R-work}$	35	12	6	90	98	27	29	0	24	92	33	24	90	100	53	96	100	47	98	33	35	27	25
P^* $_{R-free}$	-	35	29	96	94	39	37	0	71	90	39	78	84	-	82	98	-	86	100	39	37	35	35
P^*→A $_{R-work}$	49	35	33	96	98	55	53	61	0	94	63	25	98	100	55	98	100	47	100	63	59	59	57
P^*→A $_{R-free}$	-	8	10	76	80	24	18	14	0	67	14	29	63	-	59	84	-	63	76	18	12	16	18
P^*→B $_{R-work}$	10	4	4	41	57	8	10	6	2	0	6	0	25	100	22	61	100	18	55	6	10	4	6
P^*→B $_{R-free}$	-	10	12	39	59	10	10	6	20	0	6	22	27	-	49	65	-	47	55	10	8	8	8
P $_{R-work}$	35	12	10	96	100	33	29	29	27	90	0	22	94	100	47	96	100	47	98	29	25	29	27
P $_{R-free}$	-	27	31	92	94	33	31	25	69	88	0	71	86	-	82	94	-	80	92	29	27	29	33
P→A $_{R-work}$	45	29	29	96	100	55	49	59	25	90	51	0	96	100	53	100	100	49	100	57	59	51	51
P→A $_{R-free}$	-	12	12	71	80	18	16	10	31	69	16	0	63	-	61	86	-	65	82	16	16	10	12
P→B $_{R-work}$	4	2	2	41	57	8	6	4	2	31	4	2	0	100	25	65	100	18	57	4	8	6	8
P→B $_{R-free}$	-	10	12	47	65	12	8	2	20	45	6	22	0	-	51	67	-	55	69	6	10	4	10
S $_{R-work}$	0	0	0	0	0	0	0	0	0	0	0	0	0	0	0	0	22	0	0	0	0	0	0
S $_{R-free}$	-	-	-	-	-	-	-	-	-	-	-	-	-	-	-	-	-	-	-	-	-	-	-
S→A $_{R-work}$	16	8	10	82	88	29	27	33	18	69	27	10	73	100	0	86	100	24	84	37	31	35	33
S→A $_{R-free}$	-	2	2	47	45	12	10	6	18	47	8	18	37	-	0	47	-	31	47	6	4	10	10
S→B $_{R-work}$	2	0	0	18	31	0	0	0	0	16	2	0	8	100	10	0	100	2	25	0	0	0	0
S→B $_{R-free}$	-	2	0	27	31	2	2	0	10	18	4	8	14	-	47	0	-	47	27	0	0	0	0
S^* $_{R-work}$	0	0	0	0	0	0	0	0	0	0	0	0	0	31	0	0	0	0	0	0	0	0	0
S^* $_{R-free}$	-	-	-	-	-	-	-	-	-	-	-	-	-	-	-	-	-	-	-	-	-	-	-
S^*→A $_{R-work}$	29	20	18	84	92	33	35	37	18	78	35	20	76	100	39	96	100	0	94	35	33	39	35
S^*→A $_{R-free}$	-	2	2	47	49	12	8	6	22	47	8	18	43	-	45	47	-	0	47	10	6	8	8
S^*→B $_{R-work}$	2	0	0	20	31	0	0	0	0	22	2	0	6	100	14	41	100	4	0	0	0	2	4
S^*→B $_{R-free}$	-	2	0	29	41	0	0	0	12	27	2	8	14	-	49	33	-	49	0	2	2	2	4
S^*→P^* $_{R-work}$	33	12	8	92	98	33	33	29	24	86	29	22	88	100	49	98	100	43	94	0	24	27	31
S^*→P^* $_{R-free}$	-	29	25	96	98	35	31	27	67	86	29	73	82	-	78	98	-	82	96	0	24	27	31
S^*→P $_{R-work}$	35	10	4	90	96	25	24	29	22	86	31	24	88	100	47	96	100	47	96	33	0	33	31
S^*→P $_{R-free}$	-	24	22	94	96	37	29	31	71	84	37	76	88	-	82	96	-	86	94	33	0	29	31
S→P^* $_{R-work}$	37	12	10	90	96	31	33	25	16	88	31	22	92	100	49	98	100	45	94	29	22	0	25
S→P^* $_{R-free}$	-	29	25	94	94	41	29	29	71	86	35	73	88	-	82	98	-	90	96	35	24	0	31
S→P $_{R-work}$	35	10	10	90	96	24	25	25	22	88	29	20	90	100	47	98	100	43	94	29	20	27	0
S→P $_{R-free}$	-	27	29	94	96	33	27	29	73	88	37	76	88	-	86	98	-	84	96	31	33	33	0

0 100

Table 23. *Comparison of R-work/R-free (rounded to two decimal places) for the models generated from the 52 original NO-NCS data sets. Each row shows the percentage of models that a pipeline variant built with equal R-work or R-free to each other pipeline variant.*

Pipeline variant	A	A→P^*	A→P	A→B	B	B→P^*	B→P	P^*	P^*→A	P^*→B	P	P→A	P→B	S	S→A	S→B	S^*	S^*→A	S^*→B	S^*→P^*	S^*→P	S→P^*	S→P
A $_{R-work}$	100	14	14	4	2	16	18	10	29	6	16	33	2	0	33	0	0	29	2	20	14	12	20
A $_{R-free}$	-	-	-	-	-	-	-	-	-	-	-	-	-	-	-	-	-	-	-	-	-	-	-
A→P^* $_{R-work}$	14	100	59	0	0	14	20	18	20	2	20	22	0	0	18	0	0	12	0	16	20	24	20
A→P^* $_{R-free}$	-	100	43	4	2	14	22	12	8	8	22	6	4	-	8	0	-	8	0	16	20	20	20
A→P $_{R-work}$	14	59	100	0	0	14	27	25	20	2	18	22	0	0	12	0	0	12	0	20	24	18	20
A→P $_{R-free}$	-	43	100	8	2	12	18	20	6	6	10	4	2	-	6	4	-	4	2	18	27	24	14
A→B $_{R-work}$	4	0	0	100	33	10	10	8	2	39	0	4	43	2	2	27	2	4	27	6	6	6	6
A→B $_{R-free}$	-	4	8	100	22	4	2	2	10	35	4	16	37	-	10	20	-	4	20	0	4	4	2
B $_{R-work}$	2	0	0	33	100	2	0	2	2	24	0	0	37	0	2	27	0	4	35	0	2	2	2
B $_{R-free}$	-	2	2	22	100	2	6	4	10	10	2	10	27	-	10	25	-	4	18	0	0	4	2
B→P^* $_{R-work}$	16	14	14	10	2	100	67	31	18	6	16	12	8	0	10	4	0	14	4	20	29	22	25
B→P^* $_{R-free}$	-	14	12	4	2	100	53	29	12	4	22	12	4	-	10	2	-	16	6	22	24	16	27
B→P $_{R-work}$	18	20	27	10	0	67	100	29	18	6	25	18	4	0	14	6	0	12	4	18	37	20	27
B→P $_{R-free}$	-	22	18	2	6	53	100	22	14	8	20	20	2	-	12	2	-	12	4	18	29	24	24
P^* $_{R-work}$	10	18	25	8	2	31	29	100	16	2	37	18	6	0	14	4	0	16	2	37	35	47	49
P^* $_{R-free}$	-	12	20	2	4	29	22	100	16	4	35	12	14	-	12	2	-	8	0	33	31	35	35
P^*→A $_{R-work}$	29	20	20	2	2	18	18	16	100	4	10	49	0	0	27	2	0	35	0	14	20	25	22
P^*→A $_{R-free}$	-	8	6	10	10	12	14	16	100	14	18	39	18	-	24	6	-	16	12	16	18	14	10
P^*→B $_{R-work}$	6	2	2	39	24	6	6	2	4	100	4	10	43	0	10	24	0	4	24	8	4	8	6
P^*→B $_{R-free}$	-	8	6	35	10	4	8	4	14	100	6	10	27	-	4	18	-	6	18	4	8	6	4
P $_{R-work}$	16	20	18	0	0	16	25	37	10	4	100	27	2	0	25	2	0	18	0	41	43	39	43
P $_{R-free}$	-	22	10	4	2	22	20	35	18	6	100	14	8	-	10	2	-	12	6	41	35	35	29
P→A $_{R-work}$	33	22	22	4	0	12	18	18	49	10	27	100	2	0	37	0	0	31	0	22	18	27	29
P→A $_{R-free}$	-	6	4	16	10	12	20	12	39	10	14	100	16	-	22	6	-	18	10	12	8	18	12
P→B $_{R-work}$	2	0	0	43	37	8	4	6	0	43	2	2	100	0	2	27	0	6	37	8	4	2	2
P→B $_{R-free}$	-	4	2	37	27	4	2	14	18	27	8	16	100	-	12	20	-	2	18	12	2	8	2
S $_{R-work}$	0	0	0	2	0	0	0	0	0	0	0	0	0	100	0	0	47	0	0	0	0	0	0
S $_{R-free}$	-	-	-	-	-	-	-	-	-	-	-	-	-	-	-	-	-	-	-	-	-	-	-
S→A $_{R-work}$	33	18	12	2	2	10	14	14	27	10	25	37	2	0	100	4	0	37	2	14	22	16	20
S→A $_{R-free}$	-	8	6	10	10	10	12	12	24	4	10	22	12	-	100	6	-	24	4	16	14	8	4
S→B $_{R-work}$	0	0	0	27	27	4	6	4	2	24	2	0	27	0	4	100	0	2	33	2	4	2	2
S→B $_{R-free}$	-	0	4	20	25	2	2	2	6	18	2	6	20	-	6	100	-	6	39	2	4	2	2
S^* $_{R-work}$	0	0	0	2	0	0	0	0	0	0	0	0	0	47	0	0	100	0	0	0	0	0	0
S^* $_{R-free}$	-	-	-	-	-	-	-	-	-	-	-	-	-	-	-	-	-	-	-	-	-	-	-
S^*→A $_{R-work}$	29	12	12	4	4	14	12	16	35	4	18	31	6	0	37	2	0	100	2	22	20	16	22
S^*→A $_{R-free}$	-	8	4	4	4	16	12	8	16	6	12	18	2	-	24	6	-	100	4	8	8	2	8
S^*→B $_{R-work}$	2	0	0	27	35	4	4	2	0	24	0	0	37	0	2	33	0	2	100	6	4	4	2
S^*→B $_{R-free}$	-	0	2	20	18	6	4	0	12	18	6	10	18	-	4	39	-	4	100	2	4	2	0
S^*→P^* $_{R-work}$	20	16	20	6	0	20	18	37	14	8	41	22	8	0	14	2	0	22	6	100	43	43	39
S^*→P^* $_{R-free}$	-	16	18	0	0	22	18	33	16	4	41	12	12	-	16	2	-	8	2	100	43	37	37
S^*→P $_{R-work}$	14	20	24	6	2	29	37	35	20	4	43	18	4	0	22	4	0	20	4	43	100	45	49
S^*→P $_{R-free}$	-	20	27	4	0	24	29	31	18	8	35	8	2	-	14	4	-	8	4	43	100	47	35
S→P^* $_{R-work}$	12	24	18	6	2	22	20	47	25	8	39	27	2	0	16	2	0	16	4	43	45	100	47
S→P^* $_{R-free}$	-	20	24	4	4	16	24	35	14	6	35	18	8	-	8	2	-	2	2	37	47	100	35
S→P $_{R-work}$	20	20	20	6	2	25	27	49	22	6	43	29	2	0	20	2	0	22	2	39	49	47	100
S→P $_{R-free}$	-	20	14	2	2	27	24	35	10	4	29	12	2	-	4	2	-	8	0	37	35	35	100

0 100

Table 24. *Comparison of R-work/R-free (rounded to two decimal places) for the models generated from the 52 original NO-NCS data sets. Each row shows the percentage of models that a pipeline variant built with R-work or R-free at least 5% lower than each other pipeline variant.*

Pipeline variant	A	A→P^*	A→P	A→B	B	B→P^*	B→P	P^*	P^*→A	P^*→B	P	P→A	P→B	S	S→A	S→B	S^*	S^*→A	S^*→B	S^*→P^*	S^*→P	S→P^*	S→P
A $_{R-work}$	0	10	8	51	61	10	12	10	2	49	12	0	39	100	24	69	100	24	61	12	14	16	16
A $_{R-free}$	-	-	-	-	-	-	-	-	-	-	-	-	-	-	-	-	-	-	-	-	-	-	-
A→P^* $_{R-work}$	4	0	2	61	73	8	8	2	2	53	2	0	49	100	22	78	100	20	78	2	2	2	4
A→P^* $_{R-free}$	-	0	2	51	63	4	6	2	18	47	2	14	35	-	57	71	-	51	63	0	2	0	4
A→P $_{R-work}$	4	2	0	57	71	8	8	2	2	51	4	0	43	100	20	76	100	22	75	2	2	4	6
A→P $_{R-free}$	-	2	0	49	65	6	4	0	18	45	2	14	33	-	57	67	-	49	61	0	0	0	2
A→B $_{R-work}$	0	0	0	0	8	0	0	0	0	6	0	0	2	94	6	18	94	2	10	0	0	0	0
A→B $_{R-free}$	-	0	0	0	4	0	0	0	4	8	0	0	2	-	37	16	-	33	10	0	0	0	0
B $_{R-work}$	0	0	0	10	0	0	0	0	0	6	0	0	2	96	2	8	96	2	2	0	0	0	0
B $_{R-free}$	-	0	0	8	0	0	0	0	2	6	0	2	2	-	39	10	-	35	6	0	0	0	0
B→P^* $_{R-work}$	2	4	2	53	61	0	2	0	2	49	0	0	43	100	18	71	100	20	59	0	2	0	2
B→P^* $_{R-free}$	-	10	10	53	61	0	2	4	18	45	2	14	41	-	53	67	-	45	59	0	2	0	4
B→P $_{R-work}$	2	2	0	57	63	2	0	0	2	47	0	0	35	100	16	71	100	18	61	0	0	0	0
B→P $_{R-free}$	-	4	8	55	63	2	0	0	18	49	0	14	35	-	53	73	-	47	59	0	0	0	0
P^* $_{R-work}$	6	4	2	45	57	4	4	0	2	29	0	0	29	100	16	67	100	16	59	0	2	0	4
P^* $_{R-free}$	-	4	4	43	63	4	6	0	18	35	2	14	27	-	47	57	-	47	61	0	2	0	4
P^*→A $_{R-work}$	4	16	14	49	61	18	20	16	0	49	16	0	47	100	25	69	100	25	61	16	16	16	16
P^*→A $_{R-free}$	-	4	4	25	31	0	4	0	0	25	0	0	18	-	41	35	-	39	35	0	2	0	2
P^*→B $_{R-work}$	2	2	2	18	20	0	2	0	0	0	0	0	6	100	10	24	100	10	25	0	2	0	2
P^*→B $_{R-free}$	-	4	6	16	24	2	4	0	10	0	0	6	6	-	43	29	-	35	25	0	2	0	2
P $_{R-work}$	6	4	2	45	57	2	4	0	2	35	0	0	31	100	18	63	100	18	59	0	2	2	4
P $_{R-free}$	-	6	6	41	57	4	6	2	12	39	0	14	27	-	45	59	-	45	59	0	4	0	4
P→A $_{R-work}$	6	16	14	49	61	16	18	14	0	47	14	0	41	100	25	69	100	22	63	16	16	18	18
P→A $_{R-free}$	-	4	4	25	29	0	4	0	0	20	2	0	12	-	41	33	-	39	31	0	2	0	4
P→B $_{R-work}$	0	0	0	16	22	0	2	0	2	8	0	0	0	100	8	29	100	8	24	0	2	0	2
P→B $_{R-free}$	-	2	2	20	20	0	2	0	10	10	2	10	0	-	45	29	-	41	25	0	2	0	2
S $_{R-work}$	0	0	0	0	0	0	0	0	0	0	0	0	0	0	0	0	0	0	0	0	0	0	0
S $_{R-free}$	-	-	-	-	-	-	-	-	-	-	-	-	-	-	-	-	-	-	-	-	-	-	-
S→A $_{R-work}$	2	2	2	39	47	6	8	4	0	33	4	2	24	100	0	53	100	4	49	4	8	6	10
S→A $_{R-free}$	-	0	0	12	16	2	2	0	0	14	0	2	8	-	0	14	-	2	20	0	2	0	2
S→B $_{R-work}$	0	0	0	10	4	0	0	0	0	4	0	0	2	96	0	0	96	2	4	0	0	0	0
S→B $_{R-free}$	-	0	0	10	4	0	0	0	2	4	0	4	2	-	41	0	-	31	6	0	0	0	0
S^* $_{R-work}$	0	0	0	0	0	0	0	0	0	0	0	0	0	0	0	0	0	0	0	0	0	0	0
S^* $_{R-free}$	-	-	-	-	-	-	-	-	-	-	-	-	-	-	-	-	-	-	-	-	-	-	-
S^*→A $_{R-work}$	0	2	0	39	57	4	6	6	0	33	4	2	27	100	8	63	100	0	51	4	8	8	10
S^*→A $_{R-free}$	-	0	0	12	16	0	2	0	0	14	2	0	10	-	10	14	-	0	20	0	0	0	2
S^*→B $_{R-work}$	0	0	0	6	2	0	0	0	0	6	0	0	4	96	2	10	96	2	0	0	0	0	0
S^*→B $_{R-free}$	-	0	0	6	2	0	0	0	2	6	0	2	2	-	43	8	-	33	0	0	0	0	0
S^*→P^* $_{R-work}$	6	4	2	45	59	4	6	2	2	35	0	0	29	100	20	69	100	18	55	0	2	0	4
S^*→P^* $_{R-free}$	-	8	4	41	57	6	10	4	12	37	2	14	24	-	45	57	-	45	55	0	4	2	4
S^*→P $_{R-work}$	4	2	0	39	57	4	2	2	2	35	0	0	27	100	20	67	100	16	53	0	0	0	4
S^*→P $_{R-free}$	-	6	8	37	55	6	2	2	12	37	0	12	31	-	41	59	-	39	57	0	0	0	4
S→P^* $_{R-work}$	6	4	2	47	57	2	4	2	2	29	0	0	29	100	20	67	100	18	59	0	2	0	2
S→P^* $_{R-free}$	-	6	10	41	61	2	4	2	14	43	2	12	29	-	43	59	-	39	57	0	2	0	4
S→P $_{R-work}$	4	2	0	39	55	4	2	0	2	27	0	0	24	100	16	61	100	16	51	0	0	0	0
S→P $_{R-free}$	-	4	6	41	55	4	6	2	12	39	0	12	25	-	43	57	-	41	55	0	2	2	0

0 100

Table 25. *Comparison of R-work/R-free (rounded to two decimal places) for the models generated from the 52 original NO-NCS data sets. Each row shows the percentage of models that a pipeline variant built with R-work or R-free between 1% and 4% lower than each other pipeline variant.*

Pipeline variant	A	A→P^*	A→P	A→B	B	B→P^*	B→P	P^*	P^*→A	P^*→B	P	P→A	P→B	S	S→A	S→B	S^*	S^*→A	S^*→B	S^*→P^*	S^*→P	S→P^*	S→P
A $_{R-work}$	0	18	22	43	37	37	35	45	20	35	37	22	55	0	27	29	0	18	35	35	37	35	29
A $_{R-free}$	–	–	–	–	–	–	–	–	–	–	–	–	–	–	–	–	–	–	–	–	–	–	–
A→P^* $_{R-work}$	55	0	16	39	27	53	51	69	43	41	67	49	49	0	53	22	0	49	22	71	69	63	67
A→P^* $_{R-free}$	–	0	27	43	33	47	41	51	67	35	49	69	51	–	33	27	–	39	35	55	55	51	49
A→P $_{R-work}$	53	22	0	43	29	59	53	67	45	43	69	49	55	0	59	24	0	49	25	71	71	69	65
A→P $_{R-free}$	–	25	0	43	33	45	41	51	67	37	57	71	53	–	35	29	–	45	37	57	51	51	55
A→B $_{R-work}$	2	0	0	0	41	6	6	2	2	14	4	0	14	4	10	37	4	10	43	2	4	4	4
A→B $_{R-free}$	–	2	0	0	47	8	6	2	10	18	4	14	14	–	6	37	–	16	41	4	2	2	4
B $_{R-work}$	0	0	0	8	0	0	0	0	0	14	0	0	4	4	8	33	4	2	31	2	2	2	2
B $_{R-free}$	–	2	0	20	0	2	0	2	8	25	4	8	6	–	6	33	–	12	35	2	4	2	2
B→P^* $_{R-work}$	35	22	18	31	37	0	14	41	25	37	51	33	41	0	43	25	0	33	37	47	43	47	49
B→P^* $_{R-free}$	–	25	27	35	35	0	16	27	47	41	43	57	43	–	25	29	–	27	35	43	37	43	35
B→P $_{R-work}$	33	20	12	27	37	16	0	41	27	37	45	33	55	0	43	24	0	35	35	49	39	47	47
B→P $_{R-free}$	–	27	29	37	31	27	0	41	51	33	49	51	55	–	25	24	–	33	37	51	41	47	49
P^* $_{R-work}$	29	8	4	45	41	24	25	0	22	63	33	24	61	0	37	29	0	31	39	33	33	27	22
P^* $_{R-free}$	–	31	25	53	31	35	31	0	53	55	37	65	57	–	35	41	–	39	39	39	35	35	31
P^*→A $_{R-work}$	45	20	20	47	37	37	33	45	0	45	47	25	51	0	29	29	0	22	39	47	43	43	41
P^*→A $_{R-free}$	–	4	6	51	49	24	14	14	0	41	14	29	45	–	18	49	–	24	41	18	10	16	16
P^*→B $_{R-work}$	8	2	2	24	37	8	8	6	2	0	6	0	20	0	12	37	0	8	29	6	8	4	4
P^*→B $_{R-free}$	–	6	6	24	35	8	6	6	10	0	6	16	22	–	6	35	–	12	29	10	6	8	6
P $_{R-work}$	29	8	8	51	43	31	25	29	25	55	0	22	63	0	29	33	0	29	39	29	24	27	24
P $_{R-free}$	–	22	25	51	37	29	25	24	57	49	0	57	59	–	37	35	–	35	33	29	24	29	29
P→A $_{R-work}$	39	14	16	47	39	39	31	45	25	43	37	0	55	0	27	31	0	27	37	41	43	33	33
P→A $_{R-free}$	–	8	8	45	51	18	12	10	31	49	14	0	51	–	20	53	–	25	51	16	14	10	8
P→B $_{R-work}$	4	2	2	25	35	8	4	4	0	24	4	2	0	0	18	35	0	10	33	4	6	6	6
P→B $_{R-free}$	–	8	10	27	45	12	6	2	10	35	4	12	0	–	6	37	–	14	43	6	8	4	8
S $_{R-work}$	0	0	0	0	0	0	0	0	0	0	0	0	0	0	0	0	22	0	0	0	0	0	0
S $_{R-free}$	–	–	–	–	–	–	–	–	–	–	–	–	–	–	–	–	–	–	–	–	–	–	–
S→A $_{R-work}$	14	6	8	43	41	24	20	29	18	35	24	8	49	0	0	33	0	20	35	33	24	29	24
S→A $_{R-free}$	–	2	2	35	29	10	8	6	18	33	8	16	29	–	0	33	–	29	27	6	2	10	8
S→B $_{R-work}$	2	0	0	8	27	0	0	0	0	12	2	0	6	4	10	0	4	0	22	0	0	0	0
S→B $_{R-free}$	–	2	0	18	27	2	2	0	8	14	4	4	12	–	6	0	–	16	22	0	0	0	0
S^* $_{R-work}$	0	0	0	0	0	0	0	0	0	0	0	0	0	31	0	0	0	0	0	0	0	0	0
S^* $_{R-free}$	–	–	–	–	–	–	–	–	–	–	–	–	–	–	–	–	–	–	–	–	–	–	–
S^*→A $_{R-work}$	29	18	18	45	35	29	29	31	18	45	31	18	49	0	31	33	0	0	43	31	25	31	25
S^*→A $_{R-free}$	–	2	2	35	33	12	6	6	22	33	6	18	33	–	35	33	–	0	27	10	6	8	6
S^*→B $_{R-work}$	2	0	0	14	29	0	0	0	0	16	2	0	2	4	12	31	4	2	0	0	0	2	4
S^*→B $_{R-free}$	–	2	0	24	39	0	0	0	10	22	2	6	12	–	6	25	–	16	0	2	2	2	4
S^*→P^* $_{R-work}$	27	8	6	47	39	29	27	27	22	51	29	22	59	0	29	29	0	25	39	0	22	27	27
S^*→P^* $_{R-free}$	–	22	22	55	41	29	22	24	55	49	27	59	59	–	33	41	–	37	41	0	20	25	27
S^*→P $_{R-work}$	31	8	4	51	39	22	22	27	20	51	31	24	61	0	27	29	0	31	43	33	0	33	27
S^*→P $_{R-free}$	–	18	14	57	41	31	27	29	59	47	37	65	57	–	41	37	–	47	37	33	0	29	27
S→P^* $_{R-work}$	31	8	8	43	39	29	29	24	14	59	31	22	63	0	29	31	0	27	35	29	20	0	24
S→P^* $_{R-free}$	–	24	16	53	33	39	25	27	57	43	33	61	59	–	39	39	–	51	39	35	22	0	27
S→P $_{R-work}$	31	8	10	51	41	20	24	25	20	61	29	20	67	0	31	37	0	27	43	29	20	27	0
S→P $_{R-free}$	–	24	24	53	41	29	22	27	61	49	37	65	63	–	43	41	–	43	41	31	31	31	0

0 71

Appendix D
The Results of the Synthetic data sets for the Original data sets Used in Buccaneer Development

Table 1. *Complete and intermediate models produced by the 13 pipeline variants for the 52 synthetic data sets, where (T) and (C) denote intermediate models produced by pipeline executions that timed out and crashed, respectively.*

Pipeline variant	HA-NCS			MR-NCS			NO-NCS		
	Complete	Intermediate	Failed	Complete	Intermediate	Failed	Complete	Intermediate	Failed
A	258	1(T) 0(C)	0	258	1(T) 0(C)	0	258	1(T) 0(C)	0
A→P^*	259	0(T) 0(C)	0	258	0(T) 0(C)	1	259	0(T) 0(C)	0
A→B	259	0(T) 0(C)	0	259	0(T) 0(C)	0	259	0(T) 0(C)	0
B	259	0(T) 0(C)	0	259	0(T) 0(C)	0	259	0(T) 0(C)	0
B→P^*	259	0(T) 0(C)	0	259	0(T) 0(C)	0	259	0(T) 0(C)	0
P^*	259	0(T) 0(C)	0	259	0(T) 0(C)	0	257	2(T) 0(C)	0
P^*→A	259	0(T) 0(C)	0	259	0(T) 0(C)	0	259	0(T) 0(C)	0
P^*→B	259	0(T) 0(C)	0	259	0(T) 0(C)	0	259	0(T) 0(C)	0
A→P	-	-	-	-	-	-	259	0(T) 0(C)	0
B→P	-	-	-	-	-	-	258	1(T) 0(C)	0
P	-	-	-	-	-	-	256	2(T) 0(C)	1
P→A	-	-	-	-	-	-	256	2(T) 0(C)	1
P→B	-	-	-	-	-	-	258	0(T) 0(C)	1

Models used in the comparison: 259 HA-NCS, 258 MR-NCS and 258 NO-NCS.

Table 2. *Structure completeness comparison for the models generated from the 52 synthetic HA-NCS data sets. Each row corresponds to a pipeline variant, and shows the percentage (rounded to the nearest integer) of models that the pipeline variant built with higher structure completeness than each of the other pipeline variants.*

Pipeline variant	A	A→P^*	A→B	B	B→P^*	P^*	P^*→A	P^*→B
A	0	2	1	1	0	4	35	2
A→P^*	96	0	8	7	1	26	98	4
A→B	95	90	0	42	32	86	95	27
B	97	92	53	0	32	88	96	34
B→P^*	99	97	63	63	0	94	100	44
P^*	95	70	13	10	3	0	98	7
P^*→A	15	2	0	0	0	0	0	1
P^*→B	97	95	66	62	53	92	98	0

0 100

Table 3. *Structure completeness comparison for the models generated from the 52 synthetic HA-NCS data sets. Each row corresponds to a pipeline variant, and shows the percentage (rounded to the nearest integer) of models that the pipeline variant built with equal structure completeness to each of the other pipeline variants.*

Pipeline variant	A	A→P^*	A→B	B	B→P^*	P^*	$P^*\longrightarrow A$	$P^*\longrightarrow B$
A	100	2	4	3	1	1	50	1
A→P^*	2	100	2	2	2	4	1	1
A→B	4	2	100	4	5	2	4	7
B	3	2	4	100	6	1	3	3
B→P^*	1	2	5	6	100	3	0	3
P^*	1	4	2	1	3	100	2	1
$P^*\longrightarrow A$	50	1	4	3	0	2	100	1
$P^*\longrightarrow B$	1	1	7	3	3	1	1	100

0 100

Table 4. *Structure completeness comparison for the models generated from the 52 synthetic HA-NCS data sets. Each row corresponds to a pipeline variant, and shows the percentage (rounded to the nearest integer) of models that the pipeline variant built with at least 5% higher structure completeness than each of the other pipeline variants.*

Pipeline variant	A	A→P^*	A→B	B	B→P^*	P^*	$P^*\longrightarrow A$	$P^*\longrightarrow B$
A	0	1	0	0	0	3	10	1
A→P^*	79	0	2	1	1	14	82	1
A→B	90	85	0	30	22	82	90	20
B	93	89	37	0	19	86	93	22
B→P^*	97	94	47	38	0	90	98	32
P^*	93	41	8	5	1	0	94	5
$P^*\longrightarrow A$	3	0	0	0	0	0	0	0
$P^*\longrightarrow B$	93	90	54	47	42	90	94	0

0 98

Table 5. *Structure completeness comparison for the models generated from the 52 synthetic HA-NCS data sets. Each row corresponds to a pipeline variant, and shows the percentage (rounded to the nearest integer) of models that the pipeline variant built with between 1% and 4% higher structure completeness than each of the other pipeline variants.*

Pipeline variant	A	A→P^*	A→B	B	B→P^*	P^*	$P^*\longrightarrow A$	$P^*\longrightarrow B$
A	0	1	1	1	0	1	25	1
A→P^*	17	0	6	6	0	12	15	3
A→B	5	5	0	13	10	3	5	7
B	3	3	16	0	12	3	3	12
B→P^*	2	3	16	24	0	4	2	11
P^*	2	29	4	5	3	0	3	2
$P^*\longrightarrow A$	11	2	0	0	0	0	0	0
$P^*\longrightarrow B$	4	5	12	15	11	2	4	0

0 29

Table 6. *Comparison of R-work/R-free (rounded to two decimal places) for the models generated from the 52 synthetic HA-NCS data sets. Each row shows the percentage of models that a pipeline variant built with lower R-work or R-free than each other pipeline variant.*

Pipeline variant	A	A→P^*	A→B	B	B→P^*	P^*	P^*⟶A	P^*⟶B
A $_{R-work}$	0	86	96	95	92	97	29	94
A $_{R-free}$	-	-	-	-	-	-	-	-
A→P^* $_{R-work}$	9	0	92	91	67	100	2	85
A→P^* $_{R-free}$	-	0	49	49	20	53	94	44
A→B $_{R-work}$	3	7	0	41	8	41	1	30
A→B $_{R-free}$	-	42	0	44	11	44	86	33
B $_{R-work}$	4	6	48	0	3	45	1	34
B $_{R-free}$	-	44	45	0	6	48	87	33
B→P^* $_{R-work}$	7	23	87	93	0	92	2	81
B→P^* $_{R-free}$	-	76	85	90	0	85	96	80
P^* $_{R-work}$	2	0	51	49	3	0	0	41
P^* $_{R-free}$	-	45	49	48	10	0	93	36
P^*⟶A $_{R-work}$	53	97	99	98	97	100	0	98
P^*⟶A $_{R-free}$	-	4	11	12	4	6	0	10
P^*⟶B $_{R-work}$	6	11	60	58	13	53	2	0
P^*⟶B $_{R-free}$	-	50	59	57	16	54	89	0

0 100

Table 7. *Comparison of R-work/R-free (rounded to two decimal places) for the models generated from the 52 synthetic HA-NCS data sets. Each row shows the percentage of models that a pipeline variant built with equal R-work or R-free to each other pipeline variant.*

Pipeline variant	A	A→P^*	A→B	B	B→P^*	P^*	P^*⟶A	P^*⟶B
A $_{R-work}$	100	4	1	1	1	0	18	0
A $_{R-free}$	-	-	-	-	-	-	-	-
A→P^* $_{R-work}$	4	100	2	3	10	0	2	4
A→P^* $_{R-free}$	-	100	8	7	4	2	2	6
A→B $_{R-work}$	1	2	100	11	4	7	0	10
A→B $_{R-free}$	-	8	100	11	4	7	3	8
B $_{R-work}$	1	3	11	100	4	7	0	8
B $_{R-free}$	-	7	11	100	4	4	1	10
B→P^* $_{R-work}$	1	10	4	4	100	5	1	7
B→P^* $_{R-free}$	-	4	4	4	100	4	0	4
P^* $_{R-work}$	0	0	7	7	5	100	0	6
P^* $_{R-free}$	-	2	7	4	4	100	1	10
P^*⟶A $_{R-work}$	18	2	0	0	1	0	100	0
P^*⟶A $_{R-free}$	-	2	3	1	0	1	100	2
P^*⟶B $_{R-work}$	0	4	10	8	7	6	0	100
P^*⟶B $_{R-free}$	-	6	8	10	4	10	2	100

0 100

Table 8. *Comparison of R-work/R-free (rounded to two decimal places) for the models generated from the 52 synthetic HA-NCS data sets. Each row shows the percentage of models that a pipeline variant built with R-work or R-free at least 5% lower than each other pipeline variant.*

Pipeline variant	A	A→P^*	A→B	B	B→P^*	P^*	P^*→A	P^*→B
A $_{R-work}$	0	58	89	89	73	96	2	85
A $_{R-free}$	-	-	-	-	-	-	-	-
A→P^* $_{R-work}$	4	0	73	73	34	91	0	63
A→P^* $_{R-free}$	-	0	28	31	10	33	78	22
A→B $_{R-work}$	2	2	0	10	0	20	0	8
A→B $_{R-free}$	-	19	0	16	3	24	75	12
B $_{R-work}$	2	0	14	0	0	22	0	7
B $_{R-free}$	-	21	16	0	2	20	73	9
B→P^* $_{R-work}$	3	2	63	60	0	54	0	49
B→P^* $_{R-free}$	-	49	56	58	0	55	93	47
P^* $_{R-work}$	1	0	29	28	0	0	0	20
P^* $_{R-free}$	-	21	25	23	2	0	86	16
P^*→A $_{R-work}$	15	67	93	95	82	100	0	92
P^*→A $_{R-free}$	-	2	5	6	2	3	0	4
P^*→B $_{R-work}$	3	1	25	15	3	29	0	0
P^*→B $_{R-free}$	-	28	26	22	5	27	78	0

0 100

Table 9. *Comparison of R-work/R-free (rounded to two decimal places) for the models generated from the 52 synthetic HA-NCS data sets. Each row shows the percentage of models that a pipeline variant built with R-work or R-free between 1% and 4% lower than each other pipeline variant.*

Pipeline variant	A	A→P^*	A→B	B	B→P^*	P^*	P^*→A	P^*→B
A $_{R-work}$	0	29	7	6	18	2	27	9
A $_{R-free}$	-	-	-	-	-	-	-	-
A→P^* $_{R-work}$	5	0	19	18	33	9	2	22
A→P^* $_{R-free}$	-	0	22	18	10	20	17	21
A→B $_{R-work}$	1	5	0	31	8	21	1	22
A→B $_{R-free}$	-	23	0	28	7	21	12	21
B $_{R-work}$	2	6	35	0	2	22	1	28
B $_{R-free}$	-	23	29	0	5	28	14	24
B→P^* $_{R-work}$	4	20	25	33	0	37	2	31
B→P^* $_{R-free}$	-	27	29	32	0	30	3	33
P^* $_{R-work}$	1	0	22	20	3	0	0	20
P^* $_{R-free}$	-	24	23	25	8	0	7	20
P^*→A $_{R-work}$	38	30	6	3	15	0	0	7
P^*→A $_{R-free}$	-	2	6	6	2	3	0	5
P^*→B $_{R-work}$	3	10	35	42	10	24	2	0
P^*→B $_{R-free}$	-	22	33	34	11	27	10	0

0 42

Table 10. *Structure completeness comparison for the models generated from the 52 synthetic MR-NCS data sets. Each row corresponds to a pipeline variant, and shows the percentage (rounded to the nearest integer) of models that the pipeline variant built with higher structure completeness than each of the other pipeline variants.*

Pipeline variant	A	A→P^*	A→B	B	B→P^*	P^*	P^*→A	P^*→B
A	0	2	0	1	0	4	36	2
A→P^*	96	0	8	7	1	23	98	5
A→B	95	91	0	44	33	86	95	30
B	97	91	52	0	33	88	96	35
B→P^*	99	97	64	63	0	96	100	46
P^*	95	72	13	10	2	0	98	7
P^*→A	13	2	0	0	0	0	0	1
P^*→B	97	95	64	61	50	93	97	0

0 100

Table 11. *Structure completeness comparison for the models generated from the 52 synthetic MR-NCS data sets. Each row corresponds to a pipeline variant, and shows the percentage (rounded to the nearest integer) of models that the pipeline variant built with equal structure completeness to each of the other pipeline variants.*

Pipeline variant	A	A→P^*	A→B	B	B→P^*	P^*	P^*→A	P^*→B
A	100	2	5	3	1	1	51	2
A→P^*	2	100	2	2	2	5	1	1
A→B	5	2	100	4	4	1	4	6
B	3	2	4	100	5	1	3	3
B→P^*	1	2	4	5	100	2	0	5
P^*	1	5	1	1	2	100	2	1
P^*→A	51	1	4	3	0	2	100	2
P^*→B	2	1	6	3	5	1	2	100

0 100

Table 12. *Structure completeness comparison for the models generated from the 52 synthetic MR-NCS data sets. Each row corresponds to a pipeline variant, and shows the percentage (rounded to the nearest integer) of models that the pipeline variant built with at least 5% higher structure completeness than each of the other pipeline variants.*

Pipeline variant	A	A→P^*	A→B	B	B→P^*	P^*	P^*→A	P^*→B
A	0	1	0	0	0	3	10	1
A→P^*	78	0	1	1	1	13	81	1
A→B	90	85	0	32	20	84	91	22
B	93	88	38	0	17	84	93	24
B→P^*	97	95	47	46	0	91	98	37
P^*	92	43	7	4	1	0	93	3
P^*→A	3	0	0	0	0	0	0	0
P^*→B	93	91	54	47	41	91	93	0

0 98

Table 13. *Structure completeness comparison for the models generated from the 52 synthetic MR-NCS data sets. Each row corresponds to a pipeline variant, and shows the percentage (rounded to the nearest integer) of models that the pipeline variant built with between 1% and 4% higher structure completeness than each of the other pipeline variants.*

Pipeline variant	A	A→P^*	A→B	B	B→P^*	P^*	P^*→A	P^*→B
A	0	1	0	1	0	1	26	1
A→P^*	18	0	7	6	0	10	16	3
A→B	5	5	0	12	12	2	5	8
B	4	3	14	0	16	4	3	11
B→P^*	2	2	16	17	0	5	2	9
P^*	3	29	6	6	1	0	5	3
P^*→A	10	2	0	0	0	0	0	1
P^*→B	4	4	10	15	8	2	5	0

0 29

Table 14. *Comparison of R-work/R-free (rounded to two decimal places) for the models generated from the 52 synthetic MR-NCS data sets. Each row shows the percentage of models that a pipeline variant built with lower R-work or R-free than each other pipeline variant.*

Pipeline variant	A	A→P^*	A→B	B	B→P^*	P^*	P^*→A	P^*→B
A $_{R-work}$	0	87	96	95	90	98	28	94
A $_{R-free}$	-	-	-	-	-	-	-	-
A→P^* $_{R-work}$	9	0	91	91	70	100	1	87
A→P^* $_{R-free}$	-	0	50	51	22	53	93	43
A→B $_{R-work}$	3	8	0	43	9	38	1	30
A→B $_{R-free}$	-	42	0	42	10	41	86	34
B $_{R-work}$	4	7	44	0	3	46	1	34
B $_{R-free}$	-	45	47	0	5	48	86	38
B→P^* $_{R-work}$	9	21	86	95	0	92	2	81
B→P^* $_{R-free}$	-	75	85	91	0	86	97	82
P^* $_{R-work}$	2	0	55	50	3	0	0	41
P^* $_{R-free}$	-	44	51	45	10	0	93	36
P^*→A $_{R-work}$	57	97	99	99	97	100	0	98
P^*→A $_{R-free}$	-	6	11	11	3	5	0	11
P^*→B $_{R-work}$	6	9	59	55	14	51	2	0
P^*→B $_{R-free}$	-	51	58	54	13	50	88	0

0 100

Table 15. *Comparison of R-work/R-free (rounded to two decimal places) for the models generated from the 52 synthetic MR-NCS data sets. Each row shows the percentage of models that a pipeline variant built with equal R-work or R-free to each other pipeline variant.*

Pipeline variant	A	A→P^*	A→B	B	B→P^*	P^*	P^*→A	P^*→B
A $_{R-work}$	100	5	1	1	1	0	16	0
A $_{R-free}$	-	-	-	-	-	-	-	-
A→P^* $_{R-work}$	5	100	1	2	9	0	2	4
A→P^* $_{R-free}$	-	100	7	4	3	3	1	6
A→B $_{R-work}$	1	1	100	13	6	7	0	11
A→B $_{R-free}$	-	7	100	10	5	8	3	8
B $_{R-work}$	1	2	13	100	2	4	0	11
B $_{R-free}$	-	4	10	100	3	7	3	8
B→P^* $_{R-work}$	1	9	6	2	100	4	1	5
B→P^* $_{R-free}$	-	3	5	3	100	5	0	5
P^* $_{R-work}$	0	0	7	4	4	100	0	9
P^* $_{R-free}$	-	3	8	7	5	100	1	14
P^*→A $_{R-work}$	16	2	0	0	1	0	100	1
P^*→A $_{R-free}$	-	1	3	3	0	1	100	1
P^*→B $_{R-work}$	0	4	11	11	5	9	1	100
P^*→B $_{R-free}$	-	6	8	8	5	14	1	100

0 100

Table 16. *Comparison of R-work/R-free (rounded to two decimal places) for the models generated from the 52 synthetic MR-NCS data sets. Each row shows the percentage of models that a pipeline variant built with R-work or R-free at least 5% lower than each other pipeline variant.*

Pipeline variant	A	A→P^*	A→B	B	B→P^*	P^*	P^*→A	P^*→B
A $_{R-work}$	0	58	88	91	72	96	3	85
A $_{R-free}$	-	-	-	-	-	-	-	-
A→P^* $_{R-work}$	4	0	75	74	32	90	0	64
A→P^* $_{R-free}$	-	0	29	31	8	33	77	23
A→B $_{R-work}$	2	1	0	13	0	19	0	10
A→B $_{R-free}$	-	17	0	14	2	20	75	10
B $_{R-work}$	2	0	16	0	0	18	0	7
B $_{R-free}$	-	22	18	0	1	18	74	8
B→P^* $_{R-work}$	3	4	64	62	0	55	0	51
B→P^* $_{R-free}$	-	50	60	58	0	58	93	51
P^* $_{R-work}$	1	0	26	27	0	0	0	20
P^* $_{R-free}$	-	22	24	23	2	0	86	14
P^*→A $_{R-work}$	12	69	93	95	80	100	0	90
P^*→A $_{R-free}$	-	3	5	5	2	3	0	4
P^*→B $_{R-work}$	3	2	25	16	2	28	0	0
P^*→B $_{R-free}$	-	27	26	23	5	24	80	0

0 100

Table 17. *Comparison of R-work/R-free (rounded to two decimal places) for the models generated from the 52 synthetic MR-NCS data sets. Each row shows the percentage of models that a pipeline variant built with R-work or R-free between 1% and 4% lower than each other pipeline variant.*

Pipeline variant	A	A→P^*	A→B	B	B→P^*	P^*	P^*→A	P^*→B
A $_{R-work}$	0	29	7	4	17	2	25	9
A $_{R-free}$	-	-	-	-	-	-	-	-
A→P^* $_{R-work}$	5	0	16	17	38	9	1	23
A→P^* $_{R-free}$	-	0	22	20	14	21	16	20
A→B $_{R-work}$	1	7	0	30	8	19	1	21
A→B $_{R-free}$	-	25	0	28	8	21	11	25
B $_{R-work}$	2	6	28	0	3	28	1	26
B $_{R-free}$	-	23	29	0	4	30	12	30
B→P^* $_{R-work}$	6	17	22	33	0	37	2	30
B→P^* $_{R-free}$	-	25	25	33	0	28	4	31
P^* $_{R-work}$	1	0	29	23	3	0	0	21
P^* $_{R-free}$	-	21	27	22	8	0	7	22
P^*→A $_{R-work}$	44	28	6	3	17	0	0	7
P^*→A $_{R-free}$	-	3	6	6	2	2	0	7
P^*→B $_{R-work}$	3	8	34	39	12	23	2	0
P^*→B $_{R-free}$	-	24	32	31	9	26	8	0

0 44

Table 18. *Structure completeness comparison for the models generated from the 52 synthetic NO-NCS data sets. Each row corresponds to a pipeline variant, and shows the percentage (rounded to the nearest integer) of models that the pipeline variant built with higher structure completeness than each of the other pipeline variants.*

Pipeline variant	A	A→P^*	A→P	A→B	B	B→P^*	B→P	P^*	P^*→A	P^*→B	P	P→A	P→B
A	0	2	3	0	1	0	0	4	33	2	3	32	0
A→P^*	97	0	40	13	11	2	2	24	98	4	21	98	3
A→P	95	53	0	12	9	3	0	24	96	5	22	96	4
A→B	94	85	84	0	41	28	25	77	95	22	77	95	24
B	95	88	88	53	0	26	22	81	96	31	82	96	27
B→P^*	99	97	97	68	69	0	35	93	100	44	93	100	41
B→P	99	98	98	71	72	51	0	94	100	48	95	100	42
P^*	96	72	70	22	17	5	3	0	98	10	45	97	8
P^*→A	13	2	2	0	0	0	0	0	0	1	0	18	0
P^*→B	97	94	93	72	65	52	48	89	98	0	88	98	44
P	97	72	74	21	16	5	3	42	99	10	0	98	11
P→A	14	1	2	1	1	0	0	2	19	0	2	0	0
P→B	98	95	96	73	69	57	54	90	99	52	88	99	0

0 100

Table 19. *Structure completeness comparison for the models generated from the 52 synthetic NO-NCS data sets. Each row corresponds to a pipeline variant, and shows the percentage (rounded to the nearest integer) of models that the pipeline variant built with equal structure completeness to each of the other pipeline variants.*

Pipeline variant	A	A→P^*	A→P	A→B	B	B→P^*	B→P	P^*	P^*→A	P^*→B	P	P→A	P→B
A	100	2	2	5	3	1	0	0	54	1	0	54	2
A→P^*	2	100	7	2	1	2	0	4	0	2	8	1	2
A→P	2	7	100	3	3	0	2	6	2	2	4	2	0
A→B	5	2	3	100	6	4	4	1	5	5	2	4	2
B	3	1	3	6	100	5	6	2	4	4	2	3	3
B→P^*	1	2	0	4	5	100	14	3	0	3	3	0	2
B→P	0	0	2	4	6	14	100	3	0	3	2	0	4
P^*	0	4	6	1	2	3	3	100	2	2	13	1	2
P^*→A	54	0	2	5	4	0	0	2	100	1	1	63	1
P^*→B	1	2	2	5	4	3	3	2	1	100	2	1	5
P	0	8	4	2	2	3	2	13	1	2	100	0	1
P→A	54	1	2	4	3	0	0	1	63	1	0	100	1
P→B	2	2	0	2	3	2	4	2	1	5	1	1	100

0 100

Table 20. *Structure completeness comparison for the models generated from the 52 synthetic NO-NCS data sets. Each row corresponds to a pipeline variant, and shows the percentage (rounded to the nearest integer) of models that the pipeline variant built with at least 5% higher structure completeness than each of the other pipeline variants.*

Pipeline variant	A	A→P^*	A→P	A→B	B	B→P^*	B→P	P^*	P^*→A	P^*→B	P	P→A	P→B
A	0	1	2	0	0	0	0	3	9	1	3	8	0
A→P^*	74	0	17	4	2	1	0	14	77	1	12	78	1
A→P	86	24	0	7	3	1	0	10	86	2	11	86	2
A→B	86	78	76	0	28	18	16	72	86	16	73	85	14
B	90	83	81	33	0	14	9	77	90	20	76	90	15
B→P^*	97	95	91	53	49	0	10	85	98	34	86	97	28
B→P	98	94	93	55	50	15	0	87	99	36	87	99	28
P^*	94	52	41	14	9	1	0	0	94	5	12	95	5
P^*→A	4	0	2	0	0	0	0	0	0	0	0	2	0
P^*→B	93	88	88	57	50	40	37	86	94	0	84	93	30
P	93	50	43	15	10	2	1	14	95	5	0	94	5
P→A	4	1	1	0	0	0	0	2	4	0	2	0	0
P→B	93	90	91	59	52	44	43	86	94	37	86	93	0

0 99

Table 21. *Structure completeness comparison for the models generated from the 52 synthetic NO-NCS data sets. Each row corresponds to a pipeline variant, and shows the percentage (rounded to the nearest integer) of models that the pipeline variant built with between 1% and 4% higher structure completeness than each of the other pipeline variants.*

Pipeline variant	A	A→P^*	A→P	A→B	B	B→P^*	B→P	P^*	P^*→A	P^*→B	P	P→A	P→B
A	0	1	0	0	1	0	0	1	24	1	0	24	0
A→P^*	22	0	22	9	9	1	1	10	21	3	9	20	2
A→P	9	29	0	6	5	2	0	13	10	3	11	10	2
A→B	9	7	9	0	13	10	9	5	9	7	4	10	11
B	6	5	7	20	0	12	13	4	5	11	7	5	12
B→P^*	2	2	6	14	20	0	26	7	2	10	7	3	12
B→P	1	4	5	17	23	36	0	7	1	12	8	1	14
P^*	2	21	29	9	8	4	3	0	4	4	34	2	3
P^*→A	9	2	0	0	0	0	0	0	0	0	0	16	0
P^*→B	4	6	5	15	15	12	11	3	4	0	4	5	14
P	4	22	31	6	6	3	2	28	3	5	0	4	6
P→A	9	0	2	1	1	0	0	0	15	0	0	0	0
P→B	5	5	5	14	18	13	11	3	5	15	2	6	0

0 36

Table 22. *Comparison of R-work/R-free (rounded to two decimal places) for the models generated from the 52 synthetic NO-NCS data sets. Each row shows the percentage of models that a pipeline variant built with lower R-work or R-free than each other pipeline variant.*

Pipeline variant	A	A→P^*	A→P	A→B	B	B→P^*	B→P	P^*	P^*→A	P^*→B	P	P→A	P→B
A $_{R-work}$	0	87	86	96	95	90	90	97	30	94	97	32	94
A $_{R-free}$	-	-	-	-	-	-	-	-	-	-	-	-	-
A→P^* $_{R-work}$	9	0	31	95	93	74	73	100	1	89	100	2	90
A→P^* $_{R-free}$	-	0	43	59	57	24	28	52	94	50	51	89	48
A→P $_{R-work}$	10	38	0	93	93	76	75	100	2	89	100	2	89
A→P $_{R-free}$	-	43	0	60	61	26	27	52	93	50	50	90	50
A→B $_{R-work}$	3	5	5	0	41	7	8	33	1	24	34	0	24
A→B $_{R-free}$	-	33	33	0	42	8	9	35	79	29	34	80	28
B $_{R-work}$	4	5	5	45	0	2	2	37	1	30	37	1	26
B $_{R-free}$	-	34	35	43	0	5	4	36	82	31	34	82	31
B→P^* $_{R-work}$	10	19	16	90	97	0	35	86	2	82	85	3	83
B→P^* $_{R-free}$	-	72	71	89	93	0	41	81	96	84	83	95	83
B→P $_{R-work}$	9	19	18	90	97	33	0	84	2	83	83	2	83
B→P $_{R-free}$	-	69	70	88	93	43	0	78	96	82	74	95	81
P^* $_{R-work}$	2	0	0	61	57	7	9	0	0	47	33	0	48
P^* $_{R-free}$	-	45	44	59	60	12	17	0	93	47	38	93	48
P^*→A $_{R-work}$	52	97	97	99	99	96	97	100	0	98	100	34	98
P^*→A $_{R-free}$	-	5	6	16	15	4	3	6	0	10	5	43	10
P^*→B $_{R-work}$	6	7	9	65	58	12	13	45	2	0	45	2	44
P^*→B $_{R-free}$	-	45	46	64	61	13	14	43	88	0	43	84	43
P $_{R-work}$	2	0	0	62	57	4	9	31	0	48	0	0	48
P $_{R-free}$	-	45	43	59	61	13	19	44	95	50	0	94	50
P→A $_{R-work}$	53	97	97	99	98	97	97	100	38	98	100	0	99
P→A $_{R-free}$	-	8	7	16	15	4	5	6	41	12	5	0	10
P→B $_{R-work}$	5	8	7	67	63	12	11	46	1	44	45	1	0
P→B $_{R-free}$	-	47	45	63	62	12	12	48	87	50	46	88	0

0 100

Table 23. *Comparison of R-work/R-free (rounded to two decimal places) for the models generated from the 52 synthetic NO-NCS data sets. Each row shows the percentage of models that a pipeline variant built with equal R-work or R-free to each other pipeline variant.*

Pipeline variant	A	A→P^*	A→P	A→B	B	B→P^*	B→P	P^*	P^*→A	P^*→B	P	P→A	P→B
A $_{R-work}$	100	4	3	0	2	1	2	0	17	0	1	15	1
A $_{R-free}$	-	-	-	-	-	-	-	-	-	-	-	-	-
A→P^* $_{R-work}$	4	100	31	0	2	7	8	0	2	4	0	1	3
A→P^* $_{R-free}$	-	100	13	8	9	3	3	3	2	5	4	3	5
A→P $_{R-work}$	3	31	100	2	2	8	7	0	2	2	0	2	4
A→P $_{R-free}$	-	13	100	8	4	3	3	3	2	4	7	2	5
A→B $_{R-work}$	0	0	2	100	14	3	3	5	0	11	3	1	9
A→B $_{R-free}$	-	8	8	100	15	3	3	7	4	7	7	4	9
B $_{R-work}$	2	2	2	14	100	2	1	6	0	12	6	0	10
B $_{R-free}$	-	9	4	15	100	3	3	4	3	8	5	3	7
B→P^* $_{R-work}$	1	7	8	3	2	100	32	7	2	6	11	1	5
B→P^* $_{R-free}$	-	3	3	3	3	100	16	7	0	2	4	1	5
B→P $_{R-work}$	2	8	7	3	1	32	100	7	2	4	8	2	7
B→P $_{R-free}$	-	3	3	3	3	16	100	5	1	4	8	1	7
P^* $_{R-work}$	0	0	0	5	6	7	7	100	0	7	36	0	6
P^* $_{R-free}$	-	3	3	7	4	7	5	100	1	9	19	1	4
P^*→A $_{R-work}$	17	2	2	0	0	2	2	0	100	0	0	28	0
P^*→A $_{R-free}$	-	2	2	4	3	0	1	1	100	2	0	16	3
P^*→B $_{R-work}$	0	4	2	11	12	6	4	7	0	100	7	0	12
P^*→B $_{R-free}$	-	5	4	7	8	2	4	9	2	100	7	3	7
P $_{R-work}$	1	0	0	3	6	11	8	36	0	7	100	0	7
P $_{R-free}$	-	4	7	7	5	4	8	19	0	7	100	0	5
P→A $_{R-work}$	15	1	2	1	0	1	2	0	28	0	0	100	0
P→A $_{R-free}$	-	3	2	4	3	1	1	1	16	3	0	100	2
P→B $_{R-work}$	1	3	4	9	10	5	7	6	0	12	7	0	100
P→B $_{R-free}$	-	5	5	9	7	5	7	4	3	7	5	2	100

0 100

Table 24. *Comparison of R-work/R-free (rounded to two decimal places) for the models generated from the 52 synthetic NO-NCS data sets. Each row shows the percentage of models that a pipeline variant built with R-work or R-free at least 5% lower than each other pipeline variant.*

Pipeline variant	A	A→P^*	A→P	A→B	B	B→P^*	B→P	P^*	P^*→A	P^*→B	P	P→A	P→B
A $_{R-work}$	0	60	58	90	89	74	74	96	3	85	95	2	86
A $_{R-free}$	-	-	-	-	-	-	-	-	-	-	-	-	-
A→P^* $_{R-work}$	4	0	1	81	77	41	41	91	0	68	91	1	68
A→P^* $_{R-free}$	-	0	5	36	37	14	12	34	75	29	31	76	28
A→P $_{R-work}$	4	1	0	81	77	43	43	90	0	67	90	1	71
A→P $_{R-free}$	-	6	0	34	37	12	13	31	77	28	32	79	26
A→B $_{R-work}$	2	1	0	0	9	0	1	16	0	7	16	0	6
A→B $_{R-free}$	-	12	12	0	12	2	1	17	66	10	16	62	8
B $_{R-work}$	2	0	1	13	0	0	0	18	0	5	17	0	6
B $_{R-free}$	-	16	14	16	0	1	1	16	67	9	17	63	9
B→P^* $_{R-work}$	3	3	4	69	66	0	0	45	0	53	45	0	54
B→P^* $_{R-free}$	-	47	49	68	68	0	3	47	92	52	45	92	48
B→P $_{R-work}$	2	2	3	70	66	0	0	45	0	52	48	0	52
B→P $_{R-free}$	-	51	50	69	67	2	0	45	91	52	46	91	46
P^* $_{R-work}$	1	0	0	40	43	0	1	0	0	29	0	0	26
P^* $_{R-free}$	-	24	21	39	34	2	3	0	87	23	2	87	22
P^*→A $_{R-work}$	13	67	69	95	96	83	84	100	0	92	100	3	92
P^*→A $_{R-free}$	-	3	3	5	7	2	2	3	0	4	4	8	4
P^*→B $_{R-work}$	3	1	1	27	18	2	3	22	0	0	22	0	10
P^*→B $_{R-free}$	-	23	22	27	22	5	4	20	74	0	21	74	13
P $_{R-work}$	1	0	0	40	40	0	0	0	0	28	0	0	26
P $_{R-free}$	-	25	25	40	39	3	4	5	89	28	0	88	24
P→A $_{R-work}$	13	69	67	94	96	83	83	100	3	92	100	0	92
P→A $_{R-free}$	-	2	2	5	7	2	2	5	8	6	4	0	5
P→B $_{R-work}$	3	1	2	26	25	1	2	23	0	14	22	0	0
P→B $_{R-free}$	-	22	22	29	29	2	2	22	75	14	20	74	0

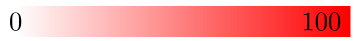

Table 25. *Comparison of R-work/R-free (rounded to two decimal places) for the models generated from the 52 synthetic NO-NCS data sets. Each row shows the percentage of models that a pipeline variant built with R-work or R-free between 1% and 4% lower than each other pipeline variant.*

Pipeline variant	A	A→P^*	A→P	A→B	B	B→P^*	B→P	P^*	P^*→A	P^*→B	P	P→A	P→B
A $_{R-work}$	0	27	28	6	6	15	15	2	28	9	2	30	8
A $_{R-free}$	-	-	-	-	-	-	-	-	-	-	-	-	-
A→P^* $_{R-work}$	5	0	31	14	17	33	32	9	1	21	9	1	22
A→P^* $_{R-free}$	-	0	38	22	20	11	16	18	19	21	20	13	20
A→P $_{R-work}$	7	37	0	12	16	33	31	10	2	21	10	1	18
A→P $_{R-free}$	-	38	0	26	24	14	14	22	16	21	18	12	25
A→B $_{R-work}$	1	4	5	0	32	6	7	18	1	17	19	0	18
A→B $_{R-free}$	-	21	21	0	30	6	7	18	13	19	18	17	20
B $_{R-work}$	2	4	4	31	0	1	2	19	1	25	20	1	21
B $_{R-free}$	-	18	21	28	0	3	3	20	16	21	17	19	22
B→P^* $_{R-work}$	7	16	12	22	31	0	35	41	2	29	40	2	28
B→P^* $_{R-free}$	-	25	22	21	25	0	37	34	4	32	37	2	35
B→P $_{R-work}$	6	17	15	20	30	33	0	39	1	31	35	2	31
B→P $_{R-free}$	-	18	20	20	26	41	0	32	4	30	28	4	35
P^* $_{R-work}$	1	0	0	21	14	7	8	0	0	18	33	0	22
P^* $_{R-free}$	-	21	23	20	25	10	14	0	6	24	36	7	26
P^*→A $_{R-work}$	40	30	28	4	3	13	13	0	0	6	0	31	7
P^*→A $_{R-free}$	-	2	3	12	9	2	2	2	0	6	2	35	6
P^*→B $_{R-work}$	3	6	9	38	40	9	10	23	2	0	22	1	34
P^*→B $_{R-free}$	-	22	24	37	39	8	10	23	13	0	22	11	31
P $_{R-work}$	1	0	0	22	17	4	9	31	0	21	0	0	22
P $_{R-free}$	-	20	18	19	22	10	15	39	5	22	0	7	26
P→A $_{R-work}$	41	28	29	5	3	14	14	0	34	6	0	0	7
P→A $_{R-free}$	-	6	5	11	8	2	2	1	34	6	2	0	6
P→B $_{R-work}$	2	7	5	41	38	11	9	22	1	30	22	1	0
P→B $_{R-free}$	-	26	23	33	32	10	10	26	12	36	26	14	0

0 41

Appendix E
The command line used in running the pipelines

E1. PHENIX AutoBuild

The following command line was used to build data set ID 1O6A (resolution 1.9 Å) and the initial model from Buccaneer.

```
phenix.autobuild \
data=PDBID.mtz \
seq_file=PDBID.fasta \
input_labels=' FP SIGFP PHIB FOM HLA HLB HLC HLD FreeR_flag ' clean_up=True \
(The following three parameters are used when run PHENIX AutoBuild after Parrot )
input_map_file=PDBID.mtz \
map_file_is_density_modified=True \
input_map_labels='FP hltofom.Phi_fom.phi hltofom.Phi_fom.fom'\
model=Buccaneer/PDBID.pdb

autobuild {
  data = "PDBID.mtz"
  model = "Buccaneer/PDBID.pdb"
  seq_file = "PDBID.fasta"
  map_file = Auto
  refinement_file = Auto
  hires_file = Auto
  crystal_info {
    unit_cell = None
    space_group = None
    solvent_fraction = None
    chain_type = *Auto PROTEIN DNA RNA
    resolution = 0
    dmax = 500
    overall_resolution = 0
    sequence = None
  }
  input_files {
    input_labels = FP SIGFP PHIB FOM HLA HLB HLC HLD FreeR_flag
    input_hires_labels = None
    input_map_labels = FP hltofom.Phi_fom.phi hltofom.Phi_fom.fom
    input_refinement_labels = None
    input_ha_file = None
    force_input_ha = False
    include_ha_in_model = True
    cif_def_file_list = None
    input_lig_file_list = None
    keep_input_ligands = True
    keep_input_waters = False
    keep_pdb_atoms = True
    remove_residues_on_special_positions = False
    refine_eff_file_list = None
    map_file_is_density_modified = True
    map_file_fom = None
    use_constant_input_map = False
    use_map_file_as_hklstart = None
    use_map_in_resolve_with_model = False
    identity_from_remark = True
```

```
    input_data_type = None
  }
  aniso {
    remove_aniso = True
    b_iso = None
    max_b_iso = 40
    target_b_ratio = 10
  }
  decision_making {
    acceptable_r = 0.25
    r_switch = 0.4
    semi_acceptable_r = 0.3
    reject_weak = False
    min_weak_z = 0.2
    min_cc_res_rebuild = 0.4
    min_seq_identity_percent = 50
    dist_close = None
    dist_close_overlap = 1.5
    loop_cc_min = 0.4
    group_ca_length = 4
    group_length = 2
    include_molprobity = False
    ok_molp_score = None
    scale_molp_score = None
  }
  density_modification {
    add_classic_denmod = None
    skip_classic_if_worse_fom = True
    skip_ncs_in_add_classic = True
    thorough_denmod = *Auto True False
    hl = False
    mask_type = *histograms probability wang classic
    mask_from_pdb = None
    mask_type_extreme_dm = histograms probability *wang classic
    mask_cycles_extreme_dm = 1
    minor_cycles_extreme_dm = 4
    wang_radius_extreme_dm = 20
    precondition = False
    minimum_ncs_cc = 0.3
    extreme_dm = False
    fom_for_extreme_dm_rebuild = 0.1
    fom_for_extreme_dm = 0.35
    rad_mask_from_pdb = 2
    modify_outside_delta_solvent = 0.05
    modify_outside_model = False
    truncate_ha_sites_in_resolve = *Auto True False
    rad_mask = None
    s_step = None
    res_start = None
    map_dmin_start = None
    map_dmin_incr = 0.25
    use_resolve_fragments = True
    use_resolve_pattern = True
    use_hl_anom_in_denmod = False
    use_hl_anom_in_denmod_with_model = False
    mask_as_mtz = False
    protein_output_mask_file = None
    ncs_output_mask_file = None
    omit_output_mask_file = None
  }
  maps {
    maps_only = False
    n_xyz_list = None
  }
  model_building {
```

```
build_type = *RESOLVE RESOLVE_AND_BUCCANEER
allow_negative_residues = False
highest_resno = None
semet = False
use_met_in_align = *Auto True False
base_model = None
consider_main_chain_list = None
dist_connect_max_helices = None
edit_pdb = True
helices_strands_only = False
resolution_helices_strands = 3.1
helices_strands_start = False
cc_helix_min = None
cc_strand_min = None
loop_lib = False
standard_loops = True
trace_loops = False
refine_trace_loops = True
density_of_points = None
max_density_of_points = None
cutout_model_radius = None
max_cutout_model_radius = 20
padding = 1
max_span = 30
max_overlap = None
min_overlap = None
include_input_model = True
input_compare_file = None
merge_models = False
morph = False
morph_main = False
dist_cut_base = 3
morph_cycles = 2
morph_rad = 7
n_ca_enough_helices = None
delta_phi = 20
offsets_list = 53 7 23
all_maps_in_rebuild = False
ps_in_rebuild = False
use_ncs_in_ps = False
remove_outlier_segments_z_cut = 3
refine = True
refine_final_model_vs_orig_data = True
reference_model = None
resolution_build = None
restart_cycle_after_morph = 5
retrace_before_build = False
reuse_chain_prev_cycle = True
richardson_rotamers = *Auto True False
rms_random_frag = None
rms_random_loop = None
start_chains_list = None
trace_as_lig = False
track_libs = False
two_fofc_denmod_in_rebuild = False
rebuild_from_fragments = False
two_fofc_in_rebuild = False
refine_map_coeff_labels = "2FOFCWT PH2FOFCWT"
filled_2fofc_maps = True
map_phasing = False
use_any_side = True
truncate_missing_side_chains = None
use_cc_in_combine_extend = False
sort_hetatms = False
map_to_object = None
```

```
}
multiple_models {
  combine_only = False
  multiple_models = False
  multiple_models_first = 1
  multiple_models_group_number = 5
  multiple_models_last = 20
  multiple_models_number = 20
  multiple_models_starting = True
  multiple_models_starting_resolution = 4
  place_waters_in_combine = None
}
ncs {
  find_ncs = *Auto True False
  input_ncs_file = None
  ncs_copies = None
  ncs_refine_coord_sigma_from_rmsd = False
  ncs_refine_coord_sigma_from_rmsd_ratio = 1
  no_merge_ncs_copies = False
  optimize_ncs = True
  use_ncs_in_build = True
  ncs_in_refinement = *torsion cartesian None
}
omit {
  composite_omit_type = *None simple_omit refine_omit sa_omit \
                        iterative_build_omit
  n_box_target = None
  n_cycle_image_min = 3
  n_cycle_rebuild_omit = 10
  offset_boundary = 2
  omit_boundary = 2
  omit_box_start = 0
  omit_box_end = 0
  omit_box_pdb_list = None
  omit_chain_list = None
  omit_offset_list = 0 0 0 0 0 0
  omit_on_rebuild = False
  omit_selection = None
  omit_region_specification = *composite_omit omit_around_pdb \
                              omit_selection
  omit_res_start_list = None
  omit_res_end_list = None
}
rebuild_in_place {
  min_seq_identity_percent_rebuild_in_place = 95
  n_cycle_rebuild_in_place = None
  n_rebuild_in_place = 1
  rebuild_chain_list = None
  rebuild_in_place = *Auto True False
  rebuild_near_chain = None
  rebuild_near_dist = 7.5
  rebuild_near_res = None
  rebuild_res_end_list = None
  rebuild_res_start_list = None
  rebuild_side_chains = False
  redo_side_chains = True
  replace_existing = True
  delete_bad_residues_only = False
  touch_up = False
  touch_up_extra_residues = None
  worst_percent_res_rebuild = 2
  smooth_range = None
  smooth_minimum_length = None
}
refinement {
```

```
    refine_b = True
    refine_se_occ = True
    skip_clash_guard = True
    correct_special_position_tolerance = None
    use_mlhl = True
    generate_hl_if_missing = False
    place_waters = True
    refinement_resolution = 0
    ordered_solvent_low_resolution = None
    link_distance_cutoff = 3
    r_free_flags_fraction = 0.1
    r_free_flags_max_free = 2000
    r_free_flags_use_lattice_symmetry = True
    r_free_flags_lattice_symmetry_max_delta = 5
    allow_overlapping = None
    fix_ligand_occupancy = None
    remove_outlier_segments = True
    twin_law = None
    max_occ = None
    refine_before_rebuild = True
    refine_with_ncs = True
    refine_xyz = True
    s_annealing = False
    skip_hexdigest = False
    use_hl_anom_in_refinement = False
    use_hl_if_present = True
  }
  thoroughness {
    build_outside = True
    connect = True
    extensive_build = False
    fit_loops = True
    insert_helices = True
    n_cycle_build = None
    n_cycle_build_max = 6
    n_cycle_build_min = 1
    n_cycle_rebuild_max = 15
    n_cycle_rebuild_min = 1
    n_mini = 10
    n_random_frag = 0
    n_random_loop = 3
    n_try_rebuild = 2
    ncycle_refine = 3
    number_of_models = None
    number_of_parallel_models = 0
    skip_combine_extend = False
    fully_skip_combine_extend = False
    thorough_loop_fit = True
  }
  general {
    coot_name = "coot"
    i_ran_seed = 72432
    raise_sorry = False
    background = True
    check_wait_time = 1
    max_wait_time = 1
    wait_between_submit_time = 1
    cache_resolve_libs = True
    resolve_size = "12"
    check_run_command = False
    run_command = "sh "
    queue_commands = None
    condor_universe = "vanilla"
    add_double_quotes_in_condor = True
    condor = None
```

```
  last_process_is_local = True
  skip_r_factor = False
  test_flag_value = Auto
  skip_xtriage = False
  base_path = None
  temp_dir = None
  clean_up = True
  print_citations = True
  solution_output_pickle_file = None
  job_title = None
  top_output_dir = None
  wizard_directory_number = None
  verbose = False
  extra_verbose = False
  debug = False
  require_nonzero = True
  remove_path_word_list = None
  fill = False
  res_fill = None
  check_only = False
  keep_files = "overall_best*" "AutoBuild_run_*.log"
  after_autosol = False
  nbatch = 3
  nproc = 1
  quick = False
  resolve_command_list = None
  resolve_pattern_command_list = None
  ignore_errors_in_subprocess = False
  send_notification = False
  notify_email = None
}
special_keywords {
  write_run_directory_to_file = None
}
run_control {
  coot = None
  ignore_blanks = None
  stop = None
  display_facts = None
  display_summary = None
  carry_on = None
  run = None
  copy_run = None
  display_runs = None
  delete_runs = None
  display_labels = None
  dry_run = False
  params_only = False
  display_all = False
}
non_user_parameters {
  gui_output_dir = None
  background_map = None
  boundary_background_map = None
  extend_try_list = True
  force_combine_extend = False
  model_list = None
  oasis_cnos = None
  offset_boundary_background_map = None
  skip_refine = False
  sg = None
  input_data_file = None
  input_map_file = "PDBID.mtz"
  input_refinement_file = Auto
  input_pdb_file = None
```

```
    input_seq_file = Auto
    super_quick = None
    require_test_set = False
  }
}
```

E2. ARP/wARP

The following command line was used to build data set ID 2ASH (resolution 2.1 Å) and the initial model from PHENIX Autobuild without Parrot.

```
set albe = 0
set arpipc =
set arpwarpdir = temp_tracing
set bcut1 = 2.0
set bcut2 = 2.0
set bcut3 = 2.0
set CCP4I_DEFFILE = UNDEFINED
set cell = '170.109 99.745 124.866 90.000 123.929 90.000'
set cgr = 1
set compareto =
set damp = '1.0 1.0'
set datafile = PDBID.mtz
set dipcut1 = 0.035
set dipcut2 = 0.010
set dipvali = 1
set emmode = 0
set fakedata = '0 0 0'
set fbest =
set flatten = 0
set fom = hltofom.Phi_fom.fom
set fp = FP
set freebuild = 0
set freelabin = 'FREE=FreeR_flag'
set freeloops = 0
set fsig = 3.2
set heavyin =
set hmainpostfit = 1
set is_semet = 0
set JOB_ID = PDBID
set keepdata = SOFTWARE_DEVELOPERS
set keepjunk = 0
set loops = 1
set modeccp4i = WARPNTRACEMODEL
set modelin = /PHENIXAutobuild/PDBID.pdb
set models = 1
set multit = 5
set ncsextension = 1
set ncsrestraints = 1
set ncsr_local = 1
set nnuc = 0
set parfile = PDBID/arp_warp_classic.par
set phaselabin =
set phaseref =
set phibest = hltofom.Phi_fom.phi
set PROJECT = COMMAND_LINE_SUBMISSION
set protsize = 0
set rand1 = 0
set rand2 = 0
set rand3 = 0
set randshift1 = 0.5
```

```
set randshift2 = 0.5
set randshift3 = 0.5
set randtimes = 0
set refmax = MLKF
set remote = 0
set remoteemail =
set resol = '103.605 2.100'
set restraints = 1
set restrcyc = 50
set restrref = 5
set ridgerestraints = 0
set rrcyc = 1
set rsig = 1.0
set sad = 0
set sadcard =
set scaleopt = 'SIMPLE LSSC ANIS'
set scalml = 'SCAL MLSC WORK'
set seqin = PDBID.fasta
set side = 1
set sidemethod = SEQQY
set sigfp = SIGFP
set skip = 0
set solvent = 1
set solventc = 1.0000
set sym = 5
set twin = 0
set upmore = 1
set version = 8.0
set warpbin = /ccp4/7.0.066/arp_warp_8.0/bin/bin-x86_64-Linux
set weightv =
set wilsonb = 44.02
set wmat = AUTO
set WORKDIR = /PDBID
set xyzlim = '0.00000 0.50000 0.00000 1.00000 0.00000 0.50000'
```

E3. Buccaneer

The following command line was used to build data set ID 1O6A (resolution 1.9 Å) and the initial model from PHENIX AutoBuild.

```
mtzin PDBID.mtz
seqin PDBID.fasta
colin-fo FP,SIGFP
colin-hl parrot.ABCD.A,parrot.ABCD.B,parrot.ABCD.C,parrot.ABCD.D
colin-free FreeR_flag
buccaneer-anisotropy-correction
buccaneer-fast
buccaneer-keyword verbose 5
cycles 5
pdbin PHENIXAutoBuild/PDBID.pdb
-------------------------- DEFAULT PARAMETERS --------------------------
title buccaneer auto-build
pdbout buccaneer.pdb
buccaneer-new-residue-name UNK
buccaneer-resolution 2.0
buccaneer-1st-cycles 3
buccaneer-1st-correlation-mode false
buccaneer-1st-sequence-reliability 0.95
buccaneer-nth-cycles 2
buccaneer-nth-correlation-mode true
buccaneer-nth-sequence-reliability 0.95
```

```
refmac-twin false
refmac-mlhl true
prefix buccaneer/
```
